# Body mass and geographic distribution determined the evolution of the wing flight-feather molt strategy in the Neornithes lineage

**DOI:** 10.1038/s41598-021-00964-6

**Published:** 2021-11-03

**Authors:** Yosef Kiat, Alex Slavenko, Nir Sapir

**Affiliations:** 1 Department of Evolutionary and Environmental Biology & Institute of Evolution, University of Haifa, 3498838 Haifa, Israel; 2 School of Biosciences, University of Sheffield, Sheffield, UK

**Keywords:** Biogeography, Evolutionary ecology, Phylogenetics

## Abstract

The evolutionary history of many organisms is characterized by major changes in morphology and distribution. Specifically, alterations of body mass and geographic distribution may profoundly influence organismal life-history traits. Here, we reconstructed the evolutionary history of flight-feather molt strategy using data from 1,808 Neornithes species. Our analysis suggests that the ancestral molt strategy of first-year birds was partial or entirely absent, and that complete wing flight-feather molt in first-year birds first evolved in the late Eocene and Oligocene (25–40 Ma), at least 30 Myr after birds first evolved. Complete flight-feather molt occurred mainly at equatorial latitudes and in relatively low body mass species, following a diversification of body mass within the lineage. We conclude that both body mass and geographic distribution shaped the evolution of molt strategies and propose that the evolutionary transition towards complete juvenile molt in the Neornithes is a novel, relatively late adaptation.

## Introduction

Modern birds (Neornithes) constitute one of the most diverse groups of terrestrial vertebrates in terms of species richness and geographic distribution. Recent advances in our knowledge of phylogenetic relationships and biogeography of Neornithes provide a better understanding of the diversification processes of this lineage^1–3^. Despite this progress, it is still unclear how distributional and morphological factors affect the evolution of basic organismal life-history and annual-routine processes, including feather molt, an important process in the avian yearly cycle.

Wing flight-feather renewal is essential for maintaining plumage utility because feathers accumulate abrasions, and accordingly, their functionality deteriorates with time^4^. Consequently, adult birds molt all of their wings’ flight-feathers periodically, and most species molt at least once per year, presumably because a longer time interval between consecutive molts is disadvantageous. Yet, first-year birds exhibit high interspecific variation in their molt strategies^4^. In many species, birds undertake their first complete wing-feather molt during the first year of their life while in other species, this first complete wing-feather molt takes place later in life and instead they either partially molt or do not molt at all in their first year^5,6^. Notably, feather molt strategy, which includes the extent, timing, duration and sequence of the molt (as well as differences in these characteristics across age groups), is an important life-history process in the avian annual cycle, with important consequences for plumage attractiveness and camouflage as well as flight aerodynamics and thermoregulation^7–10^.

Both geographic distribution and body size can substantially alter various ecological, physiological and phenological processes and consequently may affect various life-history traits^11–17^. For example, feather molt strategy in birds is affected by body mass and breeding latitude and longitude^18–21^. The amount of food required for molt increases with bird body mass, in accordance with the isometric relationship between feather mass and body size^7,22^. Moreover, the physiology of feather development sets an upper limit on the speed at which birds can grow individual feathers, resulting in a negative allometry of the primary feather growth rate in relation to body mass^7,23,24^. Consequently, heavier species require more time to complete their molt due to their larger feathers^4,20,23^.

In addition to bird size, geographic distribution may also affect the evolution of molt strategies because environmental conditions that vary across geographic ranges have direct physiological effects on birds. Environmental conditions, including seasonality and ecological interactions, may limit the availability of resources, such as food, that are essential for molting and might also restrict the time available for utilizing these resources. Consequently, molt duration, molt extent and the timing of molt within the annual cycle are strongly affected by the species’ spatio-temporal distribution, including its breeding and over-wintering latitudes and the length of its migration^8,25,26^.

Unlike adult birds, the relatively low foraging success and survival of first-year birds^27,28^ may prevent them from taking full advantage of the time and resources available for molt during the yearly cycle. In addition, juveniles fledge with fresh plumage that may enable them to forgo replacing part or all of their plumage during their first year of life^21^. Due to these factors, geographic distribution and body size could critically shape the evolution of molt in the early stages of life^29,30^. Empirical evidence^30,31^ suggest that two strategies have evolved to attain durable wings’ flight-feathers in first-year birds. The first strategy involves growing durable flight-feathers during development within the nest, or outside the nest among nidifugous species. Due to the high amount of resources that are required to create durable feathers, this strategy involves a longer nestling period over which these resources are provided to the young by their parents^32^, or a longer flightless period among nidifugous species. The second strategy involves growing relatively poor quality flight-feathers in the nest and replacing them with durable feathers soon after fledging, or after flight capability has been acquired among nidifugous species^30,31^.

The occurrence of the latter strategy, which involves the molt of all the wings’ flight-feathers (hereafter 'complete molt'), largely depends on the time available for molting the juvenile feathers during the first year of the bird's life^4,8,21^. As such, the occurrence of complete molt may be influenced by the time and resources available, both largely affected by the bird’s species distribution and body mass. Furthermore, a complete molt during the bird’s first year of life is probably the optimal strategy under favorable conditions, when food is abundant, the bird has no time constraints, and the development of the durable plumage occurs when the bird is full-grown and predation risk is lower^30^.

Here we study the physiological and environmental correlates as well as the evolutionary history of the most important component of this second strategy, comprised of relatively poor quality juvenile feathers and a complete molt during first year of the bird’s life. Towards this goal, we apply an ancestral trait reconstruction analysis in the Neornithes lineage to decipher the evolution of molt strategies in first-year birds. We also examine how the evolution of molt strategies is correlated with the evolution of body mass in modern birds, while also considering their geographic distribution. We predict that complete molt during the first year of life evolved among species who molt in localities where the time available for molt is longer (*e.g.*, close to the equator). Similarly, we predict that this strategy evolved among species with a lower body mass and hence a smaller feather surface area, due to the reduced time and resources needed in molt in these species.

## Materials and methods

### Species characteristics classification

We classified each bird species in this study by its molt strategy during the first year of life, based on information published in the literature (Supplementary Note 1), as either 'complete molt', 'partial molt' or 'absent molt' (Supplementary Table 1). We defined the molt strategy as 'complete molt' when at least most of the individuals of a species were found to molt all of their secondary and primary wings’ feathers before the end of their first year of life, either at the breeding areas (*e.g.*, *Sturnus vulgaris*) or at the over-wintering grounds, as in many migratory species (*e.g.*, *Hydroprogne caspia*). The 'partial molt' strategy was recorded in species in which the molt cycle of most individuals during the first year includes the wings’ flight-feathers but no complete molt of these feathers took place, such that the birds molted only part of their wings’ flight-feathers (*e.g.*, *Lanius senator*). The 'absent molt' strategy was recorded in species in which the molt cycle during the first year did not include molt of the wings’ flight-feathers at all (*e.g.*, *Accipiter gentilis*).

We collected species-specific mean body mass data from published literature^33,34^. To quantify the duration of the time available for molt, we developed an index which provides a basis for between-species comparison. Using published distribution maps produced by Birds of the World (Cornell Laboratory of Ornithology) and by BirdLife International and NatureServe^35,36^, we calculated the mid-distribution latitude of each species as an absolute value of the distance of each species’ mid-distribution latitude from the equator. Species-specific distribution ranges were considered as the area used by the birds during at least part of the yearly cycle, including periods of breeding, migration, or over-wintering. We used this wide definition due to the possibility that the molt took place anywhere within the species’ distribution range, including the breeding area, stop-over sites and over-wintering areas^4,8,10^. The index we used was designed to provide a proximate estimate of the time available for molt in both resident and long-distance migratory species across the entire geographic range of the species, by considering the timing of the molt within the annual cycle. In this index, a lower value represents a longer duration available for molting and a higher value represents a shorter duration available for molting. For example, resident species at high latitudes will be characterized by a high value since their molt takes place in the breeding areas and in a highly seasonal environment (far from the equator). A long-distance migratory species that breeds in high latitudes of the northern hemisphere and molts during the austral summer in the southern hemisphere will have a low value since its molt period is longer, as it takes place during a peak of resources (summer in the southern hemisphere during which the bird does not breed), similar to a tropical species^8^ (see examples of this index in Supplementary Table 2). For each bird species, we followed the taxonomy that was published by Birds of the World (Cornell Laboratory of Ornithology^35^; Supplementary Table 1).

### Phylogenetic analysis

**Figure 1:**
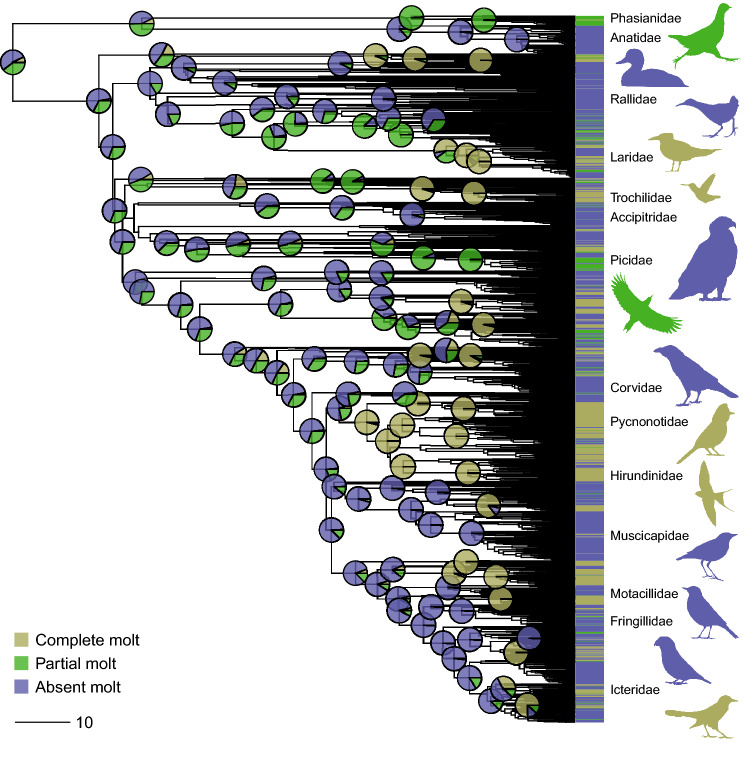
The evolutionary history of molt strategies during the first year of life among modern birds (Neornithes). Ancestral trait reconstruction analysis (1,808 species; continuous-time Markov chain, all rates different model) using a comprehensive phylogeny of modern birds^2,37^. Pie charts at the nodes denote the posterior probabilities for each of three molt strategies. Our results indicated that the ancestral molt during the first year of life for all modern birds is probably partial or absent molt and that the transition to complete wing flight-feather molt occurred independently several times in the history of this group, mainly among passerines (order Passeriformes). The scale (bottom left) represents 10 Myr.

The phylogenetic tree (Fig. 1) was obtained from an analysis of global bird diversity^2,37^, using a random sample of 1,000 trees that were downloaded from the BirdTree project using the Ericson backbone (Ericson all species: a set of 10,000 trees with 9,993 OTUs each; BirdTree.org^38^). This tree is the most detailed time-calibrated phylogeny that is currently available at the species level. The consensus tree was built using BEAST TreeAnnotator version 1.8.4 (common ancestor heights). In order to study the evolution of molt strategies during the birds’ first year of life (complete, partial or absent molt), as well as to estimate and visualize the ancestral molt strategy, we used an ancestral trait reconstruction analysis under a continuous-time Markov chain model in the R package 'phytools' (version 0.6–99; Phylogenetic Tools for Comparative Biology)^39^. We explored three models of discrete character evolution: (I) an equal transition rates model, (II) a symmetrical transition rates model and (III) an all rates different model. Then, we selected the best model based on the Akaike Information Criterion, modified for small sample sizes (AICc)^40^. We selected a specific model only if it attained ΔAICc > 2.00 compared to the other models. In addition, we estimated the Maximum Likelihood ancestral states and the evolution of bird body mass each as a continuous character evolving under a Brownian Motion model.

The phylogenetic analysis included 1,808 bird species belonging to 146 families and 27 orders in the class Neornithes (Supplementary Table 1). The phylogenetic tree included 1,629 species (90.1%) with genetic data and 179 species (9.9%) that were placed in the tree under birth–death polytomy resolution (BDPR). Due to the uncertainty involved with the BDPR method when reconstructing trait evolution^41^, we repeated the ancestral trait reconstruction analysis without these 179 species using Ericson sequenced species (a set of 10,000 trees with 6,670 OTUs each; BirdTree.org^38^; Supplementary Fig. 1).

Despite recent progress in resolving the phylogenetic relationships of modern birds, there is still no consensus regarding the higher-order topology of this group^42^. Due to this uncertainty, we repeated the ancestral trait reconstruction analysis using the phylogeny published by Prum et al.^43^ at the family level to asses if differences in the placement of higher-level taxa affected the results of the ancestral molt strategy reconstructed for modern birds. The two phylogenies we used differed mostly in the positions of a few key groups such as Grebes, Cranes, Waders, Hummingbirds, Swifts and Nightjars^42^. Therefore, we created two family-level phylogenies that included the 84 families sampled in this study, one based on the Prum et al.^43^ phylogeny (by pruning it to include only representatives of families for which we had data) and one based on the Jetz et al.^37^ phylogeny (by pruning it to include only one species per family for which we had data). We assigned a molt strategy for each family based on the reconstructed molt strategy with the highest posterior probability for the most recent common ancestor of each family in our species-level analyses described above. Overall, we had 14 families with posterior probability values supporting an ancestral complete molt, 13 families with posterior probability values supporting an ancestral partial molt and 57 families with values supporting an ancestral absent molt. Then, we performed ancestral trait reconstruction analysis as described above on these two phylogenies and compared the results between the two trees.

### Statistical analysis

We used phylogenetic logistic regressions^44^ (using the R package 'phylolm'^45^) to explore the effects of species-specific mid-distribution latitude (independent continuous factor) and mean body mass (independent continuous factor) on species-specific molt strategies during the first year of life (dependent categorical variable; 'complete molt' or 'absent molt'; n = 1,591 species): a model which fits a logistic regression of a discrete trait on continuous variables while the discrete traits change along the phylogeny according to a two-state stochastic process^46^. We fitted two univariate models (one with mass as an independent variable, and one with latitude), where molt strategy was coded as a binary dependent trait ('complete molt' arbitrarily assigned a value of 1 and 'absent molt' arbitrarily assigned a value of 0), using the species-level phylogeny to account for phylogenetic connectedness between species. Partial molt species (n = 217) were removed from the sample in this analysis due to the inability in determining their exact molt extent in the category based on the information given in the literature as this information is rarely provided. Three out of 27 orders in our sample are represented by the two molt strategies, complete and absent, and have sample sizes that are sufficiently large (> 20 species) in each category to allow order-level analysis. These order-specific phylogenetic logistic regressions were undertaken for the orders Caprimulgiformes, Charadriiformes and Passeriformes. Analyses (two-tailed, critical α = 0.05) were performed using R (version 4.1.1; R Development Core Team 2021).

## Results

In order to uncover the ecological and environmental conditions that affected the evolution of flight-feather molt strategies in first-year birds, we collected wing molt data from a total of 1,808 bird species. These included 532 species (29.4%) with a complete wing flight-feather molt; the remaining species undertake partial molt (n = 217, 12.0%) or completely avoid molting within their first year of life (n = 1,059, 58.6%; hereafter 'partial molt' or 'absent molt', respectively; Supplementary Table 1).

**Figure 2:**
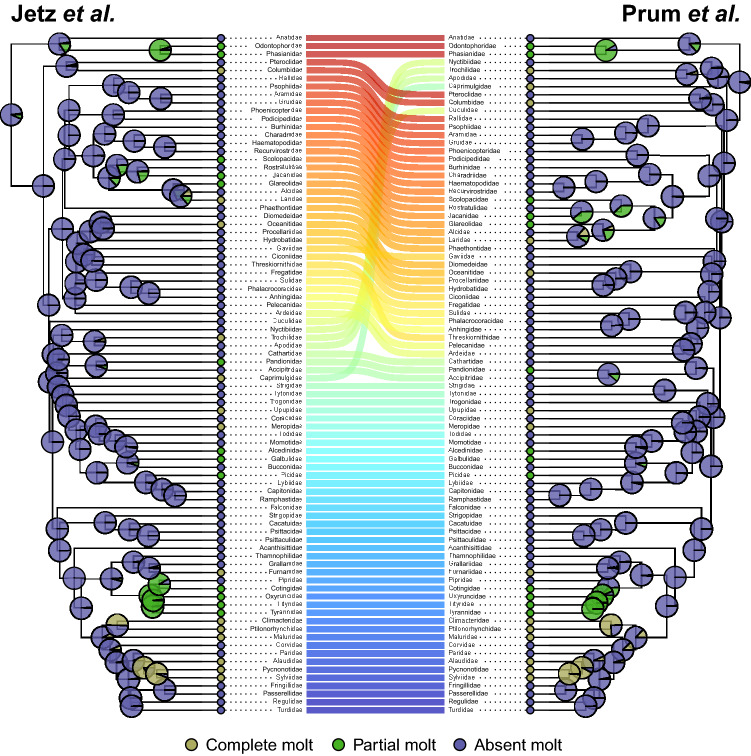
Ancestral trait reconstruction analysis (continuous-time Markov chain) among 84 modern birds families using two phylogenetic trees published by Jetz et al.^37^ (left) and by Prum et al.^43^ (right). Pie charts at the nodes denote the posterior probabilities for each of three molt strategies. Lines in the center connect the same branches on the two different trees, with the color gradient reflecting the order on the left tree, from top (red) to bottom (blue).

An ancestral trait reconstruction analysis of these 1,808 species, set in the framework of a comprehensive phylogeny of modern birds^2,37^, indicated that the most probable ancestral molt strategy among yearling modern birds is absent (posterior probability = 53.3%) or, slightly less likely, partial molt (posterior probability = 39.6) but probably not complete molt (posterior probability = 7.1%; continuous-time Markov chain, all rates different model – see *Methods*; ΔAICc > 43.52; Supplementary Table 3). Notably, among passerines (order Passeriformes; n = 1,130 species), the largest and most diverse order of birds, the posterior probability for an ancestral complete molt was much lower (1.7%; Fig. 1). In addition, the reconstructed phylogenetic tree of these three molt strategies indicated that complete molt of the wing's flight-feathers independently evolved several times in the evolutionary history of the Neornithes lineage, mainly among passerines. Furthermore, the two family-level ancestral trait reconstruction analyses using the phylogenetic trees by Prum et al.^43^ and by Jetz et al.^37^, produced very similar results. The posterior probability of complete molt among yearling modern birds as an ancestral molt strategy in these family trees is 0.8% and 1.9%, respectively (Fig. 2).

**Figure 3:**
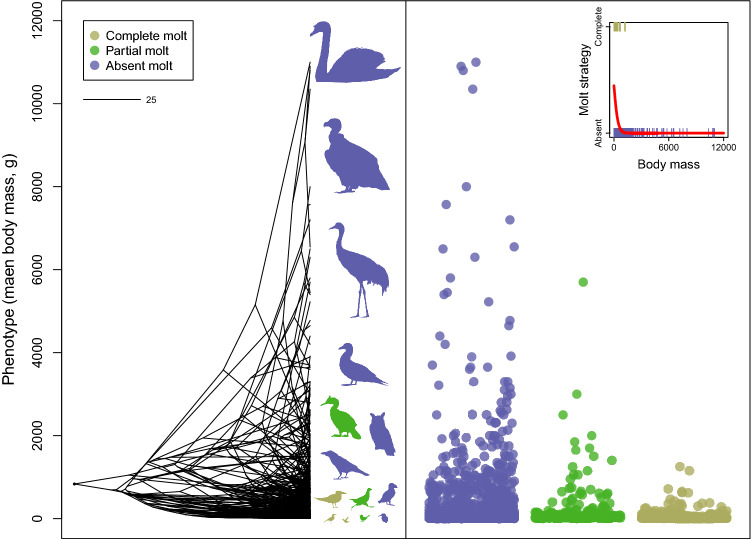
The evolutionary history of bird body mass in modern birds (Neornithes) in relation to molt strategy (complete, partial or absent molt) during the first year of the bird's life. Ancestral trait reconstruction analysis indicated that the mean body mass of the modern bird’s ancestor was slightly less than 1,000 g. Body mass diversification, mainly the declining body mass of later taxa during the evolutionary history of modern birds, took place in parallel to the evolution of complete wing flight-feather molt in yearling birds. The scale (top left) represents 25 Myr. The boxed inset (top right) displays the fitted phylogenetic logistic regression in red (complete *vs* absent molt; *P* < 0.001, n = 1,591 species).

**Figure 4:**
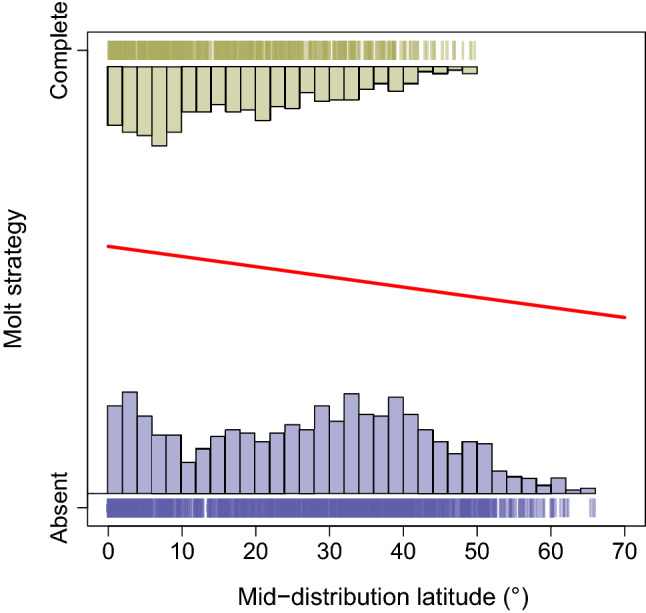
The molt strategy during the first year of the bird's life (complete *vs* absent molt) in relation to the mid-distribution latitude (°) of modern birds (Neornithes; n = 1,591 species). Our results indicated that complete wing flight-feather molt is more common in lower latitudes than in higher ones (*P* < 0.001; fitted phylogenetic logistic regression in red).

Additionally, our analysis indicated that the evolution of molt strategies during a bird’s first year of life was strongly affected by body mass and geographic distribution (*P* < 0.001; phylogenetic logistic regression; Table 1). Specifically, low body mass and overall low latitude distribution promoted the transition from partial or absent molt to a complete molt of the wings’ flight-feathers. This strategy rarely evolved among high body mass species (Fig. 3) or in species distributed away from the equatorial region, in temperate and polar regions (Fig. 4). In contrast, the order-specific analyses found that molt strategies during a bird’s first year of life was not affected by body mass among the three tested orders (Caprimulgiformes, Charadriiformes and Passeriformes, separately). Yet, latitude affected the evolution of molt strategies among two orders, Charadriiformes and Passeriformes, and not among Caprimulgiformes (Supplementary Table 4).

**Table 1:** Phylogenetic logistic regression parameter estimates for the effects of body mass (g) and latitude (°) on molt strategy (0 = absent molt, 1 = complete molt) during the first year of life of 1,591 bird species.

Parameter	*α*	Estimate (± SE)	*Z*-value	*P*-value
**Phylogenetic logistic regression with body mass as independent factor**				
Body mass	9.66E−03	− 4.25e–03 ± 6.90e−04	− 6.17	< 0.001
**Phylogenetic logistic regression with latitude as independent factor**				
Latitude	9.66E−03	− 8.96e−03 ± 2.55e−03	− 3.51	< 0.001

The results of the ancestral trait reconstruction analysis of bird body mass suggest that the mean body mass of the modern bird’s ancestor was slightly less than 1,000 g (Fig. 3). The mean body mass of species that are characterized by a complete molt during their first year of life is 50.0 g (± 106.1 g standard deviation, range 2.5 – 1,250 g; n = 532 species). In contrast, the mean body mass of species that are characterized by either a partial or absent molt during their first year of life is 387.5 g (± 1,016.9 g standard deviation, range 4.5 – 11,000 g; n = 1,276 species; Fig. 3). Furthermore, our results indicate that complete molt is more common near the equator. The mean mid-distribution latitude (absolute value) of species that are characterized by a complete molt during their first year of life is 17.4° (± 12.1° standard deviation; n = 532 species). In contrast, the mean mid-distribution latitude (absolute value) of species that are characterized by either partial or absent molt during their first year of life is 26.8° (± 16.0° standard deviation; n = 1,276 species; Fig. 4).

## Discussion

Our analysis indicated that the evolution of molt extent, an important feature of the molt strategy during the first year of life, took place in parallel with a substantial increase in the variability of bird body mass. Specifically, complete molt of flight-feathers evolved in modern birds after a considerable decrease in body mass and is rarely found among relatively heavy birds. Moreover, this strategy evolved mainly in tropical environments such as those currently located in equatorial regions where molt can take place over extended periods during the annual cycle. These two important factors, body mass and geographic distribution, vary within modern birds and influence the extent of wing feather molt in the early stage of a bird's life. These factors may have also shaped the evolution of other important life-history traits and annual-routine processes such as reproduction and migration^47–49^.

An important selection factor that affects the duration of the nestling period, in which the nestlings are vulnerable, is the risk of nest predation. A short nestling period, or flightless period among nidifugous species, which may reduce predation risk, results in the growth of poor quality feathers of the nestlings or chicks^32^. Yet, natural selection may also act against growing poor quality plumage in the early stage of the bird's life since the lower performance of this plumage could lower survival^31^. As a result, the growth of poor quality feathers by juveniles takes place only if they are later replaced by durable, higher quality feathers after fledging^30,31^. Several internal and external factors, such as solar exposure, climate, migration distance, body size, plumage coloration and sexual dichromatism level (indicative of sexual selection level), hatching date and environmental conditions related to geographic distribution, are known to influence the extent of wing feather molt in the early stage of a bird's life^4,21,29,30,50–55^. This is presumably due to their effects on the time available for this process to take place^30,51^, as well as their influence on the need to undertake an extensively molt^29^. Hence, the shortening of the risky nestling period by the growth of poor quality feathers by juveniles is possible only in those species in which juveniles have a long enough time during the first year of their life to undertake a complete molt during which they grow more durable plumage. Other factors that likely affect the durability of the juvenile feathers are low nest predation risks and high-protein nutrition provisioned for the nestlings.

Our results indicate that the evolution of complete molt during the first year of life occurred in parallel with body mass diversification and that complete molt is associated with a decrease in bird body mass (Fig. 3). This is presumably because low body mass enables molting more feathers in a given time window^20,23^. Moreover, the complete molt during the first year of life likely evolved mainly in low latitudes where conditions are usually favorable during most of the year, and hence molt can take place over an extended period throughout the annual cycle, compared to temperate or polar areas where there are greater constraints on molting due to the onset of winter conditions and associated reduction in food resources (Fig. 4). We note that none of the species exhibiting complete molt of flight-feathers had a mid-latitude distribution higher than 50°. In contrast, the order-specific analyses did not provide as clear a view about the evolution of molt strategies among the entire modern birds clade, likely because the phylogenetic signal at the order level affecting body mass, distribution and molt. The variation in the examined traits within each order is low, probably reflecting the strong phylogenetic dependency within the order. Therefore, the relationship and the influence of body mass or geographic distribution on bird molt strategy may not exist, or is substantially weaker, within orders, reinforcing the importance of considering phylogenetic scale and non-stationarity in studying macroevolutionary patterns^56,57^. For example, Passeriformes are almost all small compared to the size range of modern birds and therefore cannot be used to study how size variation may influence molt strategy.

Among species that breed in temperate or polar zones, migration to the tropics provides first-year birds a long period to complete the molt of their wings’ flight-feathers^8^, which is not possible for temperate and polar residents or short-distance migrants. Therefore, among species that breed in temperate or polar zones, the growth of poorer quality feathers by nestlings to be later replaced by more durable feathers, and the related shortening of the risky nestling period, seem to be possible mainly in species that undertake long-distance migration. We accordingly suggest that the evolution of the complete molt strategy in high-latitude species could be tightly linked to the evolution of long-distance migration to latitudes where conditions facilitate feather molt over an extended period. These long-distance trans-equatorial migrants benefit from living in two summers throughout a single annual cycle and thus presumably are experiencing abundant food resources. These birds likely need to undergo complete molt also due to the increased annual solar exposure^50^. Therefore, our work unveils the evolution of the relationships between bird body mass, species biogeography and major annual-routine processes (breeding, migration and feather molt) in the Neornithes clade.

Our results indicate that the mean body mass of modern birds’ ancestor was slightly less than 1 kg, which is compatible with known body sizes of many Mesozoic birds (Avialan), for example, *Archaeopteryx*, *Sapeornis*, *Jeholornis*, and *Confuciusornis*, compared to the larger body size of many earlier Pennaraptora species^58,59^. The diversification of bird body mass within the modern birds (Fig. 3), which included a prominent decline of body mass in several taxa^60–62^, is probably an outcome of selection forces related to flight performance^63,64^. Nevertheless, this decline of body mass apparently also had implications for the evolution of molt strategies, which likely first evolved long before the appearance of modern birds^9^. Due to the larger flight-surface and longer molt duration that are associated with high body mass, a complete molt in first-year birds remains almost impossible in relatively heavy bird species. For example, in our sample, of species with body mass > 1,000 g, yearlings of only two species (*Phasianus colchicus* and *Stercorarius maccormicki*) undergo complete molt compared to 126 species characterized by partial or absent molt. Complete molt evolved mainly among passerines (414 out of 1,130 Passeriformes species in our sample; Fig. 1), a clade characterized by relatively low body mass. Yet, it is noteworthy that complete molt of first-year birds is a common and ancestral trait in non-passerine clades characterized by low body mass, primarily if they also have tropic or temperate-tropic distribution. For example, among Hummingbirds (family Trochilidae), the clade with the lowest body mass in birds, 85.0% of species are characterized by this strategy (mean body mass = 5.1 g; n = 40 species in our sample). Nonetheless, this strategy is also common among Doves (family Columbidae) which is probably the earliest clade in which complete molt during the first year of life evolved (Fig. 1). Two more interesting clades are Bee-eaters (family Meropidae) and Terns (subfamily Sterninae), in which complete molt of yearlings is common. These two clades are widely distributed in the tropics or undertake long-distance migration into the tropics or to the opposite hemisphere during the winter at their breeding areas. Presumably, this provides the birds with ample food resources and time to complete their molt^8,26,51^.

Among passerines, the Sylvioidea superfamily is an interesting clade due to the widespread occurrence of complete molt of the wing's flight-feathers during the first year of life. This clade, which includes the Swallows, Larks, Old World Warblers and Babblers, and Bulbuls (~ 13% of all extant bird species), has an Afrotropical ancestral distribution^3^. In our sample, 82.8% of the Sylvioidea species were characterized by a complete molt during their first year of life (n = 203 species). Our ancestral trait reconstruction analysis (Fig. 1) indicated that complete molt during the first year of life evolved in Sylvioidea during the Oligocene (~ 30 Ma), while the geographic distribution of this clade was likely restricted to the Afrotropical zone^3^. Nowadays, many species of Sylvioidea that breed in the temperate and polar zones of the Palearctic, Nearctic, Neotropic, and Afrotropic, undertake their molt at or near the tropics, their ancestral molting area, where prevailing environmental conditions are usually favorable. Many of these species undertake long-distance migration to the tropics, which allows them to molt their flight-feathers during the non-breeding period. This strategy is common among most Swallows (family Hirundinidae), many Reed-Warblers (family Acrocephalidae), Grass-Warblers (family Locustellidae), and several species of other Sylvioidea families (*e.g.*, Sylviidae and Phylloscopidae).

The phylogenetic analysis in this study includes a large sample of modern birds. We included an extensive and comprehensive sample of all the species for which we were able to obtain data. This work is one of the largest phylogenetic analyzes published to date, which includes ~ 17% of all extant bird species, and encompasses 146 families (58.6%) and 27 orders (65.9%) of extant modern birds. Therefore, although our sample is incomplete, it is still representative of the diversity of modern birds, as well as their molt strategies. Yet, data on molt strategy is still sorely lacking for many bird species and clades, and future phylogenetic analyzes may benefit from increased sampling, which would require a strong foundation of fieldwork.

In this work we reconstructed the evolution of flight-feather molt strategy during the first year of life among modern birds and found that partial or absent molt is the ancestral molt strategy. Our phylogenetic analysis suggests that body size diversification, and specifically the evolution of small body size, as well as low latitude geographic distribution, are important internal and external factors that affected the evolution of complete flight-feather molt among species in the Neornithes lineage. These findings have multiple implications for the evolution of other life-history processes, biogeography and bird reproductive ecology.

## Supplementary Information

Supplementary Information.

## Data Availability

The phylogenetic trees generated and used in this study are available at OSF: https://osf.io/s8rn2/.

## References

[1] Jarvis ED (2014). Whole-genome analyses resolve early branches in the tree of life of modern birds. Science.

[2] Jetz W, Thomas GH, Joy JB, Hartmann K, Mooers AO (2012). The global diversity of birds in space and time. Nature.

[3] Claramunt S, Cracraft J (2015). A new time tree reveals Earth history’s imprint on the evolution of modern birds. Sci. Adv..

[4] Jenni, L. & Winkler, R. *Moult and Ageing of European Passerines*. (Bloomsbury Publishing, 2020).

[5] Ginn, H. B. & Melville, D. S. *Moult in Birds (BTO guide)*. (British Trust for Ornithology, 1983).

[6] Stresemann, E. & Stresemann, V. *Die Mauser der Vögel*. (Friedländer, 1966).

[7] Jenni, L. & Winkler, R. *The Biology of Moult in Birds*. (Bloomsbury Publishing, 2020).

[8] Kiat Y, Izhaki I, Sapir N (2019). The effects of long-distance migration on the evolution of moult strategies in Western-Palearctic passerines. Biol. Rev..

[9] Kiat Y (2020). Sequential molt in a feathered dinosaur and implications for early paravian ecology and locomotion. Curr. Biol..

[10] Pyle, P. *Identification guide to North American birds: A Compendium of Information on Identifying, Ageing, and Sexing ‘Near-Passerines’ and Passerines in the Hand*. (Slate Creek Press, 1997).

[11] Berlow EL, Brose U, Martinez ND (2008). The, “Goldilocks factor” in food webs. Proc. Natl. Acad. Sci..

[12] Dale J, Dey CJ, Delhey K, Kempenaers B, Valcu M (2015). The effects of life history and sexual selection on male and female plumage colouration. Nature.

[13] Kleiber M (1932). Body size and metabolism. Hilgardia.

[14] McKinnon L (2010). Lower predation risk for migratory birds at high latitudes. Science.

[15] Meiri S, Dayan T, Simberloff D (2005). Biogeographical patterns in the Western Palearctic: the fasting-endurance hypothesis and the status of Murphy’s rule. J. Biogeogr..

[16] Millar JS, Hickling GJ (1990). Fasting endurance and the evolution of mammalian body size. Funct. Ecol..

[17] Peters, R. H. & Peters, R. H. *The Ecological Implications of Body Size*. vol. 2 (Cambridge University Press, 1986).

[18] Pérez-Granados C (2020). Time available for moulting shapes inter- and intra-specific variability in post-juvenile moult extent in wheatears (genus *Oenanthe*). J. Ornithol..

[19] Hemborg C, Sanz J, Lundberg A (2001). Effects of latitude on the trade-off between reproduction and moult: a long-term study with Pied Flycatcher. Oecologia.

[20] de la Hera, I., Díaz, J. a., Pérez-Tris, J. & Tellería, J. L. A comparative study of migratory behaviour and body mass as determinants of moult duration in passerines. *J. Avian Biol.* **40**, 461–465 (2009).

[21] Kiat Y, Sapir N (2017). Age-dependent modulation of songbird summer feather moult by temporal and functional constraints. Am. Nat..

[22] Møller AP (2015). The allometry of number of feathers in birds changes seasonally. Avian Res..

[23] Rohwer S, Ricklefs RE, Rohwer VG, Copple MM (2009). Allometry of the duration of flight feather molt in birds. PLoS Biol..

[24] Rohwer VG, Rohwer S (2013). How do birds adjust the time required to replace their flight feathers?. Auk.

[25] Barta Z (2006). Annual routines of non-migratory birds: optimal moult strategies. Oikos.

[26] Barta, Z. *et al.* Optimal moult strategies in migratory birds. *Philos. Trans. R. Soc. London B Biol. Sci.* **363**, 211–229 (2008). 10.1098/rstb.2007.2136 PMC2606747 17681914

[27] Wunderle JM (1991). Age-specific foraging proficiency in birds. Curr. Ornithol..

[28] Marchetti K, Price T (1989). Differences in the foraging of juvenile and adult birds: the importance of developmental constraints. Biol. Rev..

[29] Delhey K (2020). Partial or complete? The evolution of post-juvenile moult strategies in passerine birds. J. Anim. Ecol..

[30] Kiat Y, Izhaki I (2016). Why renew fresh feathers? Advantages and conditions for the evolution of complete post-juvenile moult. J. Avian Biol..

[31] Kiat Y, Sapir N (2018). Life-history trade-offs result in evolutionary optimization of feather quality. Biol. J. Linn. Soc..

[32] Callan LM, La Sorte FA, Martin TE, Rohwer VG (2019). Higher nest predation favors rapid fledging at the cost of plumage quality in nestling birds. Am. Nat..

[33] Del Hoyo J, Elliott A, Sargatal J, Christie DA, de Juana E (2019). Handbook of the Birds of the World Alive.

[34] Dunning Jr, J. B. *CRC Handbook of Avian Body Masses*. (CRC Press, 2007).

[35] Billerman, S. M., Keeney, B. K., Rodewald, P. G. & Schulenberg, T. S. *Birds of the World*. (Cornell Laboratory of Ornithology, 2020).

[36] Bird species distribution maps of the world. *BirdLife International* (2019).

[37] Jetz W (2014). Global distribution and conservation of evolutionary distinctness in birds. Curr. Biol..

[38] Rubolini, D., Liker, A., Garamszegi, L. Z., Møller, A. P. & Saino, N. Using the BirdTree.org website to obtain robust phylogenies for avian comparative studies: a primer. *Curr. Zool.* **61**, 959–965 (2015). 10.1093/czoolo/61.6.959 PMC7098689 32256531

[39] Revell LJ (2012). phytools: an R package for phylogenetic comparative biology (and other things). Methods Ecol. Evol..

[40] Akaike H (1987). Factor analysis and AIC. Psychometrika.

[41] Rabosky DL (2015). No substitute for real data: a cautionary note on the use of phylogenies from birth–death polytomy resolvers for downstream comparative analyses. Evolution.

[42] Thomas GH (2015). An avian explosion. Nature.

[43] Prum RO (2015). A comprehensive phylogeny of birds (Aves) using targeted next-generation DNA sequencing. Nature.

[44] Ives AR, Garland T (2010). Phylogenetic logistic regression for binary dependent variables. Syst. Biol..

[45] Tung Ho, L. si & Ané, C. A linear-time algorithm for Gaussian and non-Gaussian trait evolution models. *Syst. Biol.* **63**, 397–408 (2014). 10.1093/sysbio/syu005 24500037

[46] Felsenstein J (2012). A comparative method for both discrete and continuous characters using the threshold model. Am. Nat..

[47] Cody, M. L. A general theory of clutch size. *Evolution* 174–184 (1966). 10.1111/j.1558-5646.1966.tb03353.x 28563630

[48] Newton, I. *The Migration Ecology of Birds*. (Academic Press, 2010).

[49] Newton, I. *Speciation and Biogeography of Birds*. (Academic Press, 2003).

[50] Terrill RS, Seeholzer GF, Wolfe JD (2020). Evolution of breeding plumages in birds: a multiple-step pathway to seasonal dichromatism in New World warblers (Aves: Parulidae). Ecol. Evol..

[51] Fogden MPL (1972). The seasonality and population dynamics of equatorial forest birds in Sarawak. Ibis.

[52] Kiat Y, Davaasuren B, Erdenechimeg T, Troupin D, Sapir N (2020). Large-scale longitudinal climate gradient across the Palearctic region affects passerine feather moult extent. Ecography.

[53] Kiat Y, Vortman Y, Sapir N (2019). Feather moult and bird appearance are correlated with global warming over the last 200 years. Nat. Commun..

[54] Bojarinova JG, Lehikoinen E, Eeva T (1999). Dependence of postjuvenile moult on hatching date, condition and sex in the Great Tit. J. Avian Biol..

[55] Ryzhanovsky VN (2017). Subspecies-specific features of molt in the Common Chiffchaff (*Phylloscopus collybita*) from Europe and Western Siberia. Russ. J. Ecol..

[56] Slavenko A (2019). Global patterns of body size evolution in squamate reptiles are not driven by climate. Glob. Ecol. Biogeogr..

[57] Graham CH, Storch D, Machac A (2018). Phylogenetic scale in ecology and evolution. Glob. Ecol. Biogeogr..

[58] Hone DWE, Dyke GJ, Haden M, Benton MJ (2008). Body size evolution in Mesozoic birds. J. Evol. Biol..

[59] Xu X (2014). An integrative approach to understanding bird origins. Science.

[60] Berv JS, Field DJ (2018). Genomic signature of an avian Lilliput effect across the K-Pg extinction. Syst. Biol..

[61] Dececchi TA, Larsson HCE (2013). Body and limb size dissociation at the origin of birds: uncoupling allometric constraints across a macroevolutionary transition. Evolution.

[62] Puttick MN, Thomas GH, Benton MJ (2014). High rates of evolution preceded the origin of birds. Evolution.

[63] Vizcaíno SF, Fariña RA (1999). On the flight capabilities and distribution of the giant Miocene bird Argentavis magnificens (Teratornithidae). Lethaia.

[64] McNeill Alexander R (1998). All-time giants: the largest animals and their problems. Palaeontology.

