# Supplementary material for: Body mass and geographic distribution determined the evolution of the wing flight-feather molt strategy in the Neornithes lineage
Source: Sci Rep. 2021 Nov 3;11:21573. doi: 10.1038/s41598-021-00964-6 (PMC8566465; doi:10.1038/s41598-021-00964-6)
Supplement: Supplementary file 1 — Supplementary Information. [file 41598_2021_964_MOESM1_ESM.pdf]

Body mass and geographic distribution determined the evolution of the wing flight-feather molt strategy in the Neornithes lineage

Supplementary Information

Authors: Yosef Kiat, Alex Slavenko and Nir Sapir

Supplementary Table 1. List of bird species that were included in the phylogenetic tree (Figure 1). This list includes information about the molt strategy during the first year of life (complete, partial or absent molt), mean body mass (g), and mid-distribution latitude (absolute value; °). The reference list for the molt strategy data is provided in Supplementary Note 1.

	Species	Familiy	Tree	Molt		Mean body mass (g)	Mid-distribution latitude (absolute value; °)
1	*Cyrtonyx montezumae*	Odontophoridae	Genetic-based	Partial	[9]	185	26.437
2	*Oreortyx pictus*	Odontophoridae	Genetic-based	Partial	[9]	235	38.633
3	*Colinus virginianus*	Odontophoridae	Genetic-based	Partial	[9]	180	30.102
4	*Callipepla squamata*	Odontophoridae	Genetic-based	Partial	[9]	185	43.456
5	*Callipepla gambelii*	Odontophoridae	Genetic-based	Partial	[9]	170	32.304
6	*Callipepla californica*	Odontophoridae	Genetic-based	Partial	[9]	185	37.320
7	*Synoicus ypsilophorus*	Phasianidae	Genetic-based	Partial	[71]	105	22.597
8	*Alectoris rufa*	Phasianidae	Genetic-based	Partial	[36]	490	43.280
9	*Perdix perdix*	Phasianidae	Genetic-based	Partial	[36]	450	50.538
10	*Phasianus colchicus*	Phasianidae	Genetic-based	Complete	[36]	1150	36.723
11	*Chrysolophus pictus*	Phasianidae	Genetic-based	Complete	[36]	625	29.382
12	*Meleagris gallopavo*	Phasianidae	Genetic-based	Partial	[131]	5700	33.772
13	*Bonasa umbellus*	Phasianidae	Genetic-based	Partial	[95]	575	51.235
14	*Lagopus lagopus*	Phasianidae	Genetic-based	Partial	[36]	650	61.157
15	*Lagopus muta*	Phasianidae	Genetic-based	Partial	[36]	610	58.845
16	*Falcipennis canadensis*	Phasianidae	Genetic-based	Partial	[95]	340	56.143
17	*Tetrao tetrix*	Phasianidae	Genetic-based	Partial	[36]	1050	55.057
18	*Tetrao urogallus*	Phasianidae	Genetic-based	Partial	[36]	3000	55.568
19	*Centrocercus minimus*	Phasianidae	Genetic-based	Partial	[95]	1650	38.431
20	*Centrocercus urophasianus*	Phasianidae	Genetic-based	Partial	[95]	1850	42.738
21	*Dendragapus fuliginosus*	Phasianidae	Birth-death polytomy resolution	Partial	[95]	1050	46.516
22	*Dendragapus obscurus*	Phasianidae	Genetic-based	Partial	[8]	1245	49.493
23	*Tympanuchus pallidicinctus*	Phasianidae	Genetic-based	Partial	[95]	750	34.511
24	*Tympanuchus cupido*	Phasianidae	Genetic-based	Partial	[95]	950	39.942
25	*Tympanuchus phasianellus*	Phasianidae	Genetic-based	Partial	[95]	730	52.706
26	*Dendrocygna bicolor*	Anatidae	Genetic-based	Absent	[9]	760	4.399
27	*Dendrocygna autumnalis*	Anatidae	Genetic-based	Absent	[9]	800	1.853
28	*Cereopsis novaehollandiae*	Anatidae	Genetic-based	Absent	[71]	3700	37.770
29	*Cygnus olor*	Anatidae	Genetic-based	Absent	[5]	10800	44.305
30	*Cygnus cygnus*	Anatidae	Genetic-based	Absent	[5]	10350	50.538
31	*Cygnus buccinator*	Anatidae	Genetic-based	Absent	[95]	10900	47.814
32	*Cygnus columbianus*	Anatidae	Genetic-based	Absent	[5]	6550	46.510
33	*Branta bernicla*	Anatidae	Genetic-based	Absent	[93]	1350	51.270
34	*Branta ruficollis*	Anatidae	Genetic-based	Absent	[5]	1250	57.485
35	*Branta canadensis*	Anatidae	Genetic-based	Absent	[5]	3917	48.918
36	*Branta hutchinsii*	Anatidae	Genetic-based	Absent	[9]	3150	45.341
37	*Branta leucopsis*	Anatidae	Genetic-based	Absent	[5]	1750	65.362
38	*Anser canagicus*	Anatidae	Genetic-based	Absent	[93]	2130	60.231
39	*Anser caerulescens*	Anatidae	Genetic-based	Absent	[5]	2625	50.237
40	*Anser rossii*	Anatidae	Genetic-based	Absent	[93]	1500	49.055
41	*Anser anser*	Anatidae	Genetic-based	Absent	[5]	3300	44.675
42	*Anser brachyrhynchus*	Anatidae	Genetic-based	Absent	[5]	2500	65.726
43	*Anser erythropus*	Anatidae	Genetic-based	Absent	[5]	2100	47.876
44	*Anser fabalis*	Anatidae	Genetic-based	Absent	[5]	2800	49.131
45	*Anser albifrons*	Anatidae	Genetic-based	Absent	[5]	2600	45.397
46	*Oxyura jamaicensis*	Anatidae	Genetic-based	Absent	[93]	620	38.966
47	*Nomonyx dominicus*	Anatidae	Genetic-based	Absent	[95]	360	3.012
48	*Spatula querquedula*	Anatidae	Genetic-based	Absent	[5]	400	23.494
49	*Spatula clypeata*	Anatidae	Genetic-based	Absent	[5]	630	33.956
50	*Spatula rhynchotis*	Anatidae	Genetic-based	Absent	[71]	680	32.578
51	*Spatula discors*	Anatidae	Genetic-based	Absent	[5]	400	27.915
52	*Spatula cyanoptera*	Anatidae	Genetic-based	Absent	[93]	385	1.940
53	*Mareca strepera*	Anatidae	Genetic-based	Absent	[5]	750	28.278
54	*Mareca americana*	Anatidae	Genetic-based	Absent	[93]	725	37.033
55	*Mareca penelope*	Anatidae	Genetic-based	Absent	[5]	725	34.925
56	*Anas platyrhynchos*	Anatidae	Genetic-based	Absent	[36]	1150	40.814
57	*Anas rubripes*	Anatidae	Genetic-based	Absent	[93]	1150	42.392
58	*Anas fulvigula*	Anatidae	Genetic-based	Absent	[93]	930	26.777
59	*Anas acuta*	Anatidae	Genetic-based	Absent	[5]	830	34.979

60	*Anas crecca*	Anatidae	Genetic-based	Absent	5	325	35.585
61	*Anas castanea*	Anatidae	Genetic-based	Absent	71	660	29.673
62	*Anas gracilis*	Anatidae	Genetic-based	Absent	71	500	22.049
63	*Hymenolaimus malacorhynchos*	Anatidae	Genetic-based	Absent	71	860	41.837
64	*Chenonetta jubata*	Anatidae	Genetic-based	Absent	71	810	28.245
65	*Netta rufina*	Anatidae	Genetic-based	Absent	5	1150	33.407
66	*Aythya australis*	Anatidae	Genetic-based	Absent	71	800	27.188
67	*Aythya nyroca*	Anatidae	Genetic-based	Absent	5	600	32.310
68	*Aythya fuligula*	Anatidae	Genetic-based	Absent	5	780	35.198
69	*Aythya marila*	Anatidae	Genetic-based	Absent	5	1050	48.205
70	*Aythya affinis*	Anatidae	Genetic-based	Absent	95	710	38.186
71	*Aythya valisineria*	Anatidae	Genetic-based	Absent	95	1280	41.854
72	*Aythya ferina*	Anatidae	Genetic-based	Absent	5	850	33.262
73	*Aythya collaris*	Anatidae	Genetic-based	Absent	95	710	37.743
74	*Aythya americana*	Anatidae	Genetic-based	Absent	95	980	39.887
75	*Aix galericulata*	Anatidae	Genetic-based	Absent	36	575	39.755
76	*Aix sponsa*	Anatidae	Genetic-based	Absent	36	688	37.524
77	*Alopochen aegyptiaca*	Anatidae	Genetic-based	Absent	5	1950	3.889
78	*Tadorna tadorna*	Anatidae	Genetic-based	Absent	5	1050	45.846
79	*Tadorna variegata*	Anatidae	Genetic-based	Absent	71	1500	40.893
80	*Tadorna ferruginea*	Anatidae	Genetic-based	Absent	5	1300	31.604
81	*Tadorna tadornoides*	Anatidae	Genetic-based	Absent	71	1400	34.844
82	*Clangula hyemalis*	Anatidae	Genetic-based	Absent	5	725	51.569
83	*Histrionicus histrionicus*	Anatidae	Genetic-based	Absent	95	560	50.851
84	*Polysticta stelleri*	Anatidae	Genetic-based	Absent	95	870	59.991
85	*Somateria fischeri*	Anatidae	Genetic-based	Absent	95	1360	65.939
86	*Somateria spectabilis*	Anatidae	Genetic-based	Absent	5	1600	60.255
87	*Somateria mollissima*	Anatidae	Genetic-based	Absent	5	2050	58.932
88	*Melanitta nigra*	Anatidae	Genetic-based	Absent	5	1130	49.635
89	*Melanitta fusca*	Anatidae	Genetic-based	Absent	5	1580	54.593
90	*Melanitta perspicillata*	Anatidae	Genetic-based	Absent	5	935	48.587
91	*Bucephala albeola*	Anatidae	Genetic-based	Absent	95	390	44.065
92	*Bucephala islandica*	Anatidae	Genetic-based	Absent	95	830	51.531
93	*Bucephala clangula*	Anatidae	Genetic-based	Absent	5	980	46.225
94	*Mergellus albellus*	Anatidae	Genetic-based	Absent	5	660	46.783
95	*Lophodytes cucullatus*	Anatidae	Genetic-based	Absent	95	600	41.978
96	*Mergus merganser*	Anatidae	Genetic-based	Absent	95	1500	46.851
97	*Mergus serrator*	Anatidae	Genetic-based	Absent	5	1075	46.682
98	*Pterocles orientalis*	Pteroclidae	Genetic-based	Absent	3	430	34.898
99	*Columbina inca*	Columbidae	Genetic-based	Complete	89	44	22.783
100	*Columbina passerina*	Columbidae	Genetic-based	Complete	102	33	12.577
101	*Alopecoenas stairi*	Columbidae	Birth-death polytomy resolution	Partial	99	170	17.072
102	*Alopecoenas xanthonurus*	Columbidae	Birth-death polytomy resolution	Partial	100	120	14.991
103	*Turtur tympanistria*	Columbidae	Birth-death polytomy resolution	Complete	24	66	12.258
104	*Oena capensis*	Columbidae	Genetic-based	Complete	141	41	1.742
105	*Ptilinopus roseicapilla*	Columbidae	Birth-death polytomy resolution	Complete	100	92	14.699
106	*Hemiphaga novaeseelandiae*	Columbidae	Genetic-based	Complete	71	630	40.933
107	*Ducula pacifica*	Columbidae	Genetic-based	Partial	99	395	-12.078
108	*Leptotila cassini*	Columbidae	Genetic-based	Partial	134	155	11.608
109	*Geotrygon montana*	Columbidae	Genetic-based	Partial	56	115	3.844
110	*Zenaida asiatica*	Columbidae	Genetic-based	Complete	89	150	22.752
111	*Zenaida macroura*	Columbidae	Genetic-based	Complete	11	125	30.990
112	*Patagioenas fasciata*	Columbidae	Genetic-based	Complete	89	345	32.198
113	*Streptopelia chinensis*	Columbidae	Genetic-based	Complete	71	160	1.442
114	*Streptopelia senegalensis*	Columbidae	Genetic-based	Complete	113	114	8.915
115	*Streptopelia turtur*	Columbidae	Genetic-based	Complete	36	135	34.968
116	*Streptopelia capicola*	Columbidae	Genetic-based	Complete	113	150	9.305
117	*Streptopelia decaocto*	Columbidae	Genetic-based	Complete	52	180	17.849
118	*Streptopelia semitorquata*	Columbidae	Genetic-based	Complete	113	235	8.060
119	*Ixobrychus minutus*	Ardeidae	Genetic-based	Absent	82	105	12.641
120	*Ixobrychus exilis*	Ardeidae	Genetic-based	Absent	82	71	10.631
121	*Botaurus lentiginosus*	Ardeidae	Genetic-based	Absent	9	750	36.471
122	*Botaurus poiciloptilus*	Ardeidae	Birth-death polytomy resolution	Absent	71	1150	33.327
123	*Nycticorax nycticorax*	Ardeidae	Genetic-based	Absent	112	690	0.780
124	*Nyctanassa violacea*	Ardeidae	Genetic-based	Absent	9	680	7.444
125	*Egretta garzetta*	Ardeidae	Genetic-based	Absent	5	495	5.060
126	*Egretta thula*	Ardeidae	Genetic-based	Absent	9	370	2.885
127	*Egretta caerulea*	Ardeidae	Genetic-based	Absent	9	350	7.511

128	*Egretta rufescens*	Ardeidae	Genetic-based	Absent	[9]	615	20.745
129	*Egretta tricolor*	Ardeidae	Genetic-based	Absent	[9]	350	14.003
130	*Butorides virescens*	Ardeidae	Genetic-based	Absent	[9]	230	24.952
131	*Ardea alba*	Ardeidae	Genetic-based	Absent	[9]	875	2.276
132	*Bubulcus ibis*	Ardeidae	Genetic-based	Absent	[9]	380	1.155
133	*Ardea pacifica*	Ardeidae	Genetic-based	Absent	[71]	900	25.486
134	*Ardea cinerea*	Ardeidae	Genetic-based	Absent	[5]	1550	17.797
135	*Ardea herodias*	Ardeidae	Genetic-based	Absent	[9]	2300	28.327
136	*Pelecanus occidentalis*	Pelecanidae	Genetic-based	Absent	[9]	3000	18.422
137	*Pelecanus erythrorhynchos*	Pelecanidae	Genetic-based	Absent	[95]	7200	34.892
138	*Fregata magnificens*	Fregatidae	Genetic-based	Absent	[95]	1500	0.499
139	*Morus bassanus*	Sulidae	Genetic-based	Absent	[95]	2950	45.163
140	*Sula sula*	Sulidae	Genetic-based	Absent	[95]	950	0.995
141	*Sula leucogaster*	Sulidae	Genetic-based	Absent	[95]	1250	5.955
142	*Sula nebouxii*	Sulidae	Genetic-based	Absent	[95]	1550	6.593
143	*Sula dactylatra*	Sulidae	Genetic-based	Absent	[95]	1750	2.534
144	*Anhinga anhinga*	Anhingidae	Genetic-based	Absent	[9]	1350	0.554
145	*Phalacrocorax carunculatus*	Ardeidae	Birth-death polytomy resolution	Absent	[71]	2550	40.945
146	*Phalacrocorax punctatus*	Ardeidae	Genetic-based	Absent	[71]	1200	41.577
147	*Phalacrocorax atriceps*	Phalacrocoracidae	Genetic-based	Partial	[101]	2500	52.274
148	*Phalacrocorax auritus*	Phalacrocoracidae	Genetic-based	Absent	[95]	1900	38.280
149	*Phalacrocorax brasilianus*	Phalacrocoracidae	Genetic-based	Absent	[95]	1300	-13.075
150	*Phalacrocorax aristotelis*	Phalacrocoracidae	Genetic-based	Absent	[5]	1780	49.686
151	*Phalacrocorax carbo*	Phalacrocoracidae	Genetic-based	Absent	[95]	2310	14.561
152	*Phalacrocorax penicillatus*	Phalacrocoracidae	Genetic-based	Absent	[95]	2300	41.124
153	*Phalacrocorax urile*	Phalacrocoracidae	Genetic-based	Absent	[95]	2000	55.754
154	*Phalacrocorax pelagicus*	Phalacrocoracidae	Genetic-based	Absent	[95]	1880	46.989
155	*Gavia stellata*	Gaviidae	Genetic-based	Absent	[5]	1725	52.174
156	*Gavia arctica*	Gaviidae	Genetic-based	Absent	[5]	2350	51.663
157	*Gavia pacifica*	Gaviidae	Genetic-based	Absent	[95]	2000	50.377
158	*Gavia adamsii*	Gaviidae	Genetic-based	Absent	[5]	5225	55.788
159	*Gavia immer*	Gaviidae	Genetic-based	Absent	[5]	3650	51.398
160	*Mycteria americana*	Ciconiidae	Genetic-based	Absent	[95]	2500	1.225
161	*Eudocimus albus*	Threskiornithidae	Genetic-based	Absent	[9]	900	15.655
162	*Platalea ajaja*	Threskiornithidae	Genetic-based	Absent	[95]	1500	4.865
163	*Plegadis falcinellus*	Threskiornithidae	Genetic-based	Partial	[9]	650	3.210
164	*Plegadis chihi*	Threskiornithidae	Genetic-based	Absent	[9]	615	2.796
165	*Oceanodroma castro*	Hydrobatidae	Genetic-based	Absent	[95]	45	12.299
166	*Oceanodroma leucorhoa*	Hydrobatidae	Genetic-based	Absent	[5]	46	14.313
167	*Oceanodroma homochroa*	Hydrobatidae	Genetic-based	Absent	[9]	40	33.905
168	*Oceanodroma markhami*	Hydrobatidae	Genetic-based	Absent	[9]	53	6.466
169	*Oceanodroma melania*	Hydrobatidae	Birth-death polytomy resolution	Absent	[9]	60	15.930
170	*Oceanodroma furcata*	Hydrobatidae	Genetic-based	Absent	[95]	55	46.873
171	*Hydrobates pelagicus*	Hydrobatidae	Genetic-based	Absent	[5]	26.5	13.699
172	*Phoebastria albatrus*	Diomedeidae	Genetic-based	Absent	[95]	6300	39.139
173	*Phoebastria nigripes*	Diomedeidae	Genetic-based	Absent	[65]	3300	0.027
174	*Phoebastria immutabilis*	Diomedeidae	Genetic-based	Absent	[65]	2500	39.003
175	*Thalassarche salvini*	Diomedeidae	Genetic-based	Absent	[71]	3900	32.674
176	*Thalassarche melanophris*	Diomedeidae	Genetic-based	Absent	[85]	3650	39.505
177	*Thalassarche chrysostoma*	Diomedeidae	Genetic-based	Absent	[85]	3600	51.482
178	*Oceanites oceanicus*	Oceanitidae	Genetic-based	Complete	[95]	30	12.489
179	*Thalassoica antarctica*	Procellariidae	Genetic-based	Absent	[71]	600	54.492
180	*Fulmarus glacialis*	Procellariidae	Genetic-based	Absent	[5]	780	60.655
181	*Pterodroma hasitata*	Procellariidae	Genetic-based	Absent	[95]	460	24.581
182	*Pterodroma ultima*	Procellariidae	Birth-death polytomy resolution	Absent	[95]	385	6.205
183	*Pterodroma cookii*	Procellariidae	Genetic-based	Absent	[95]	210	0.891
184	*Pterodroma inexpectata*	Procellariidae	Genetic-based	Absent	[95]	345	9.833
185	*Calonectris diomedea*	Procellariidae	Genetic-based	Absent	[5]	640	7.214
186	*Puffinus gavia*	Procellariidae	Genetic-based	Complete	[71]	365	38.780
187	*Puffinus lherminieri*	Procellariidae	Genetic-based	Absent	[95]	215	25.227
188	*Puffinus opisthomelas*	Procellariidae	Genetic-based	Absent	[95]	410	27.943
189	*Puffinus puffinus*	Procellariidae	Genetic-based	Absent	[5]	460	0.196
190	*Ardenna pacifica*	Procellariidae	Genetic-based	Absent	[71]	435	1.567
191	*Ardenna bulleri*	Procellariidae	Genetic-based	Absent	[95]	390	0.715
192	*Ardenna grisea*	Procellariidae	Genetic-based	Absent	[95]	820	0.782
193	*Ardenna gravis*	Procellariidae	Genetic-based	Absent	[95]	830	4.810
194	*Ardenna tenuirostris*	Procellariidae	Genetic-based	Absent	[95]	700	2.849
195	*Ardenna creatopus*	Procellariidae	Genetic-based	Absent	[95]	730	10.446

196	*Ardenna carneipes*	Procellariidae	Genetic-based	Absent	[95]	650	7.169
197	*Phaethon aethereus*	Phaethontidae	Genetic-based	Absent	[95]	700	6.695
198	*Phaethon lepturus*	Phaethontidae	Genetic-based	Absent	[95]	330	3.219
199	*Phaethon rubricauda*	Phaethontidae	Genetic-based	Absent	[9]	715	3.714
200	*Psophia crepitans*	Psophiidae	Genetic-based	Absent	[56]	1150	2.594
201	*Aramus guarauna*	Aramidae	Genetic-based	Absent	[9]	1150	4.038
202	*Antigone canadensis*	Gruidae	Genetic-based	Absent	[95]	4200	48.541
203	*Grus grus*	Gruidae	Genetic-based	Absent	[55]	5400	39.302
204	*Grus americana*	Gruidae	Genetic-based	Absent	[95]	6500	43.230
205	*Coturnicops noveboracensis*	Rallidae	Genetic-based	Absent	[9]	55	40.702
206	*Laterallus jamaicensis*	Rallidae	Genetic-based	Absent	[9]	30	0.384
207	*Laterallus spilonota*	Rallidae	Birth-death polytomy resolution	Absent	[9]	40	0.353
208	*Porphyrio martinica*	Rallidae	Genetic-based	Absent	[95]	210	0.571
209	*Porphyrio porphyrio*	Rallidae	Genetic-based	Absent	[9]	660	0.417
210	*Porphyrio hochstetteri*	Rallidae	Genetic-based	Absent	[71]	2500	45.485
211	*Crex crex*	Rallidae	Genetic-based	Absent	[5]	155	16.144
212	*Gallirallus philippensis*	Rallidae	Genetic-based	Absent	[71]	170	14.023
213	*Gallirallus australis*	Rallidae	Genetic-based	Absent	[71]	1050	41.283
214	*Rallus elegans*	Rallidae	Genetic-based	Absent	[9]	335	32.487
215	*Rallus longirostris*	Rallidae	Genetic-based	Absent	[9]	300	8.063
216	*Rallus aquaticus*	Rallidae	Genetic-based	Absent	[5]	115	45.200
217	*Rallus limicola*	Rallidae	Genetic-based	Absent	[9]	85	19.007
218	*Zapornia pusilla*	Rallidae	Genetic-based	Absent	[5]	34	4.541
219	*Gallinula tenebrosa*	Rallidae	Birth-death polytomy resolution	Absent	[71]	525	20.953
220	*Zapornia tabuensis*	Rallidae	Genetic-based	Absent	[71]	45	16.141
221	*Porzana carolina*	Rallidae	Genetic-based	Absent	[9]	85	26.789
222	*Porzana porzana*	Rallidae	Genetic-based	Absent	[5]	87.5	18.394
223	*Tribonyx ventralis*	Rallidae	Birth-death polytomy resolution	Absent	[71]	400	26.884
224	*Gallinula chloropus*	Rallidae	Genetic-based	Absent	[5]	285	14.793
225	*Fulica atra*	Rallidae	Genetic-based	Absent	[5]	660	9.869
226	*Fulica americana*	Rallidae	Genetic-based	Absent	[9]	650	33.733
227	*Phoenicopterus ruber*	Phoenicopteridae	Genetic-based	Absent	[95]	3100	16.642
228	*Podilymbus podiceps*	Podicipedidae	Genetic-based	Absent	[9]	415	5.334
229	*Tachybaptus ruficollis*	Podicipedidae	Genetic-based	Absent	[5]	185	13.003
230	*Aechmophorus clarkii*	Podicipedidae	Genetic-based	Absent	[95]	1100	39.144
231	*Aechmophorus occidentalis*	Podicipedidae	Genetic-based	Absent	[9]	1300	38.181
232	*Podiceps nigricollis*	Podicipedidae	Genetic-based	Absent	[5]	360	13.580
233	*Podiceps grisegena*	Podicipedidae	Genetic-based	Absent	[5]	950	47.365
234	*Podiceps cristatus*	Podicipedidae	Genetic-based	Absent	[5]	1000	10.043
235	*Podiceps auritus*	Podicipedidae	Genetic-based	Absent	[5]	385	45.496
236	*Burhinus oedicnemus*	Burhinidae	Genetic-based	Absent	[5]	410	28.809
237	*Pluvialis fulva*	Charadriidae	Genetic-based	Absent	[57]	165	15.570
238	*Pluvialis squatarola*	Charadriidae	Genetic-based	Absent	[109]	280	12.740
239	*Pluvialis dominica*	Charadriidae	Genetic-based	Complete	[95]	155	10.977
240	*Haematopus bachmani*	Haematopodidae	Genetic-based	Absent	[9]	550	42.802
241	*Haematopus palliatus*	Haematopodidae	Genetic-based	Absent	[9]	550	3.959
242	*Haematopus ostralegus*	Haematopodidae	Genetic-based	Absent	[5]	590	33.006
243	*Haematopus unicolor*	Haematopodidae	Genetic-based	Absent	[71]	720	40.855
244	*Haematopus finschi*	Haematopodidae	Genetic-based	Absent	[71]	550	40.565
245	*Himantopus novaezelandiae*	Recurvirostridae	Genetic-based	Absent	[71]	220	40.313
246	*Himantopus himantopus*	Recurvirostridae	Genetic-based	Absent	[71]	190	7.160
247	*Himantopus mexicanus*	Recurvirostridae	Genetic-based	Absent	[9]	170	24.916
248	*Recurvirostra novaehollandiae*	Recurvirostridae	Birth-death polytomy resolution	Absent	[71]	310	25.519
249	*Recurvirostra americana*	Recurvirostridae	Genetic-based	Absent	[9]	320	34.144
250	*Recurvirostra avosetta*	Recurvirostridae	Genetic-based	Absent	[5]	300	12.150
251	*Charadrius morinellus*	Charadriidae	Genetic-based	Partial	[95]	110	52.420
252	*Thinornis novaeseelandiae*	Charadriidae	Genetic-based	Absent	[71]	60	44.308
253	*Elseyornis melanops*	Charadriidae	Genetic-based	Absent	[71]	33	28.722
254	*Charadrius dubius*	Charadriidae	Genetic-based	Absent	[5]	40	17.697
255	*Charadrius hiaticula*	Charadriidae	Genetic-based	Partial	[95]	60	22.873
256	*Charadrius melodus*	Charadriidae	Genetic-based	Absent	[9]	55	38.572
257	*Charadrius semipalmatus*	Charadriidae	Genetic-based	Partial	[95]	50	12.873
258	*Charadrius vociferus*	Charadriidae	Genetic-based	Absent	[9]	100	25.124
259	*Vanellus miles*	Charadriidae	Genetic-based	Absent	[71]	360	16.777
260	*Vanellus vanellus*	Charadriidae	Genetic-based	Absent	[5]	229	42.162
261	*Anarhynchus frontalis*	Charadriidae	Genetic-based	Absent	[71]	55	39.882
262	*Charadrius obscurus*	Charadriidae	Birth-death polytomy resolution	Absent	[71]	155	46.707
263	*Charadrius bicinctus*	Charadriidae	Genetic-based	Absent	[71]	60	33.743

264	*Charadrius ruficapillus*	Charadriidae	Genetic-based	Absent	[71]	40	25.947
265	*Charadrius wilsonia*	Charadriidae	Genetic-based	Partial	[9]	60	11.770
266	*Charadrius nivosus*	Charadriidae	Genetic-based	Absent	[95]	42	4.786
267	*Charadrius montanus*	Charadriidae	Genetic-based	Absent	[9]	100	36.167
268	*Charadrius veredus*	Charadriidae	Genetic-based	Partial	[71]	95	11.038
269	*Charadrius leschenaultii*	Charadriidae	Genetic-based	Partial	[71]	90	6.658
270	*Charadrius mongolus*	Charadriidae	Genetic-based	Partial	[6]	70	11.044
271	*Rostratula benghalensis*	Rostratulidae	Genetic-based	Absent	[71]	130	3.266
272	*Jacana spinosa*	Jacanidae	Genetic-based	Partial	[95]	115	16.422
273	*Bartramia longicauda*	Scolopacidae	Genetic-based	Absent	[9]	160	13.903
274	*Numenius borealis*	Scolopacidae	Birth-death polytomy resolution	Absent	[9]	375	8.336
275	*Numenius tahitiensis*	Scolopacidae	Genetic-based	Partial	[72]	400	18.312
276	*Numenius phaeopus*	Scolopacidae	Genetic-based	Absent	[9]	400	7.707
277	*Numenius americanus*	Scolopacidae	Genetic-based	Absent	[9]	650	30.619
278	*Numenius minutus*	Scolopacidae	Genetic-based	Partial	[71]	160	19.536
279	*Numenius arquata*	Scolopacidae	Genetic-based	Absent	[5]	815	18.174
280	*Limosa limosa*	Scolopacidae	Genetic-based	Absent	[5]	340	15.994
281	*Limosa lapponica*	Scolopacidae	Genetic-based	Absent	[5]	370	13.620
282	*Limosa fedoa*	Scolopacidae	Genetic-based	Absent	[9]	340	33.085
283	*Limosa haemastica*	Scolopacidae	Genetic-based	Absent	[71]	350	7.452
284	*Limnodromus scolopaceus*	Scolopacidae	Genetic-based	Partial	[95]	110	41.957
285	*Limnodromus griseus*	Scolopacidae	Genetic-based	Partial	[95]	113	23.093
286	*Limnodromus semipalmatus*	Scolopacidae	Birth-death polytomy resolution	Absent	[71]	180	19.956
287	*Scolopax minor*	Scolopacidae	Genetic-based	Partial	[79]	190	37.940
288	*Scolopax rusticola*	Scolopacidae	Genetic-based	Absent	[5]	300	38.554
289	*Gallinago hardwickii*	Scolopacidae	Birth-death polytomy resolution	Complete	[71]	160	2.880
290	*Gallinago gallinago*	Scolopacidae	Genetic-based	Absent	[5]	95	29.245
291	*Coenocorypha aucklandica*	Scolopacidae	Genetic-based	Absent	[71]	110	51.164
292	*Coenocorypha pusilla*	Scolopacidae	Genetic-based	Absent	[71]	80	44.310
293	*Xenus cinereus*	Scolopacidae	Genetic-based	Partial	[71]	50	16.170
294	*Phalaropus tricolor*	Scolopacidae	Genetic-based	Partial	[13]	80	2.489
295	*Phalaropus fulicarius*	Scolopacidae	Genetic-based	Partial	[95]	57	12.471
296	*Phalaropus lobatus*	Scolopacidae	Genetic-based	Partial	[95]	34	16.366
297	*Actitis hypoleucos*	Scolopacidae	Genetic-based	Complete	[5]	47.5	14.473
298	*Actitis macularius*	Scolopacidae	Genetic-based	Partial	[9]	38	13.279
299	*Tringa solitaria*	Scolopacidae	Genetic-based	Partial	[9]	48	15.990
300	*Tringa ochropus*	Scolopacidae	Genetic-based	Absent	[5]	86	21.890
301	*Tringa semipalmata*	Scolopacidae	Genetic-based	Absent	[9]	260	10.753
302	*Tringa melanoleuca*	Scolopacidae	Genetic-based	Absent	[14]	175	3.939
303	*Tringa nebularia*	Scolopacidae	Genetic-based	Absent	[5]	215	12.153
304	*Tringa stagnatilis*	Scolopacidae	Genetic-based	Partial	[71]	70	11.165
305	*Tringa totanus*	Scolopacidae	Genetic-based	Absent	[5]	120	18.415
306	*Tringa flavipes*	Scolopacidae	Genetic-based	Absent	[14]	80	7.329
307	*Tringa incana*	Scolopacidae	Genetic-based	Partial	[9]	110	11.901
308	*Tringa brevipes*	Scolopacidae	Genetic-based	Partial	[71]	110	13.485
309	*Arenaria interpres*	Scolopacidae	Genetic-based	Absent	[5]	137	15.252
310	*Arenaria melanocephala*	Scolopacidae	Genetic-based	Absent	[9]	130	45.875
311	*Calidris canutus*	Scolopacidae	Genetic-based	Partial	[5]	150	14.182
312	*Calidris tenuirostris*	Scolopacidae	Genetic-based	Partial	[71]	145	15.655
313	*Calidris virgata*	Scolopacidae	Genetic-based	Absent	[9]	200	13.957
314	*Calidris pugnax*	Scolopacidae	Genetic-based	Partial	[95]	155	18.360
315	*Calidris acuminata*	Scolopacidae	Birth-death polytomy resolution	Partial	[95]	78	13.632
316	*Calidris ferruginea*	Scolopacidae	Genetic-based	Partial	[5]	80	15.875
317	*Calidris temminckii*	Scolopacidae	Genetic-based	Partial	[5]	25	35.659
318	*Calidris bairdii*	Scolopacidae	Genetic-based	Complete	[95]	40	13.336
319	*Calidris subruficollis*	Scolopacidae	Genetic-based	Complete	[95]	70	18.520
320	*Calidris subminuta*	Scolopacidae	Genetic-based	Absent	[103]	28	12.742
321	*Calidris melanotos*	Scolopacidae	Genetic-based	Complete	[9]	70	12.227
322	*Calidris mauri*	Scolopacidae	Genetic-based	Absent	[9]	25	26.930
323	*Calidris pusilla*	Scolopacidae	Genetic-based	Partial	[95]	35	24.115
324	*Calidris himantopus*	Scolopacidae	Genetic-based	Partial	[9]	55	16.730
325	*Calidris minutilla*	Scolopacidae	Genetic-based	Absent	[80]	26	26.867
326	*Calidris ruficollis*	Scolopacidae	Genetic-based	Partial	[95]	35	15.228
327	*Calidris alpina*	Scolopacidae	Genetic-based	Absent	[5]	59	29.679
328	*Calidris ptilocnemis*	Scolopacidae	Genetic-based	Absent	[9]	85	50.832
329	*Calidris maritima*	Scolopacidae	Genetic-based	Absent	[5]	75	57.847
330	*Calidris fuscicollis*	Scolopacidae	Genetic-based	Complete	[95]	47	11.214
331	*Calidris alba*	Scolopacidae	Genetic-based	Partial	[95]	75	13.566

332	*Calidris minuta*	Scolopacidae	Genetic-based	Complete	[5]	26	21.752
333	*Glareola maldivarum*	Glareolidae	Genetic-based	Partial	[71]	75	11.057
334	*Stercorarius longicaudus*	Stercorariidae	Genetic-based	Complete	[9]	310	6.355
335	*Stercorarius parasiticus*	Stercorariidae	Genetic-based	Complete	[5]	470	10.662
336	*Stercorarius maccormicki*	Stercorariidae	Genetic-based	Complete	[95]	1250	3.244
337	*Stercorarius skua*	Stercorariidae	Genetic-based	Partial	[5]	1400	48.952
338	*Stercorarius pomarinus*	Stercorariidae	Genetic-based	Complete	[95]	715	13.988
339	*Cerorhinca monocerata*	Alcidae	Genetic-based	Absent	[96]	533	44.749
340	*Fratercula cirrhata*	Alcidae	Genetic-based	Absent	[96]	773	50.878
341	*Fratercula arctica*	Alcidae	Genetic-based	Absent	[5]	460	55.006
342	*Fratercula corniculata*	Alcidae	Genetic-based	Absent	[96]	612	53.518
343	*Ptychoramphus aleuticus*	Alcidae	Genetic-based	Absent	[96]	175	40.375
344	*Aethia cristatella*	Alcidae	Genetic-based	Absent	[96]	260	51.578
345	*Aethia pygmaea*	Alcidae	Genetic-based	Absent	[96]	116	53.203
346	*Aethia psittacula*	Alcidae	Genetic-based	Absent	[96]	297	47.955
347	*Aethia pusilla*	Alcidae	Genetic-based	Absent	[96]	85	52.502
348	*Synthliboramphus antiquus*	Alcidae	Genetic-based	Absent	[96]	206	43.762
349	*Synthliboramphus craveri*	Alcidae	Genetic-based	Absent	[96]	139	30.893
350	*Synthliboramphus hypoleucus*	Alcidae	Genetic-based	Absent	[96]	158	37.348
351	*Cepphus columba*	Alcidae	Genetic-based	Absent	[96]	500	50.645
352	*Cepphus grylle*	Alcidae	Genetic-based	Absent	[5]	500	62.174
353	*Brachyramphus brevirostris*	Alcidae	Genetic-based	Absent	[96]	224	61.292
354	*Brachyramphus marmoratus*	Alcidae	Genetic-based	Absent	[96]	195	48.216
355	*Uria lomvia*	Alcidae	Genetic-based	Absent	[96]	945	52.323
356	*Uria aalge*	Alcidae	Genetic-based	Absent	[5]	1000	55.254
357	*Alca torda*	Alcidae	Genetic-based	Absent	[5]	710	54.325
358	*Alle alle*	Alcidae	Genetic-based	Absent	[96]	163	58.527
359	*Onychoprion aleuticus*	Laridae	Genetic-based	Complete	[9]	120	32.922
360	*Onychoprion fuscatus*	Laridae	Genetic-based	Complete	[95]	195	3.205
361	*Onychoprion anaethetus*	Laridae	Genetic-based	Complete	[9]	145	2.808
362	*Sternula antillarum*	Laridae	Genetic-based	Complete	[9]	45	22.360
363	*Sternula albifrons*	Laridae	Genetic-based	Complete	[5]	45	10.978
364	*Gelochelidon nilotica*	Laridae	Genetic-based	Complete	[9]	175	5.648
365	*Hydroprogne caspia*	Laridae	Genetic-based	Complete	[9]	650	9.498
366	*Chlidonias albostriatus*	Laridae	Genetic-based	Absent	[71]	92	43.405
367	*Chlidonias hybrida*	Laridae	Genetic-based	Complete	[71]	84	8.214
368	*Chlidonias leucopterus*	Laridae	Genetic-based	Partial	[33]	65	12.633
369	*Chlidonias niger*	Laridae	Genetic-based	Complete	[5]	73	15.438
370	*Thalasseus maximus*	Laridae	Genetic-based	Partial	[9]	370	1.677
371	*Thalasseus bergii*	Laridae	Genetic-based	Complete	[71]	360	6.754
372	*Thalasseus elegans*	Laridae	Genetic-based	Complete	[9]	260	7.179
373	*Thalasseus sandvicensis*	Laridae	Genetic-based	Complete	[5]	246	8.890
374	*Sterna forsteri*	Laridae	Genetic-based	Partial	[9]	160	31.087
375	*Sterna hirundo*	Laridae	Genetic-based	Complete	[5]	117	9.304
376	*Sterna dougallii*	Laridae	Genetic-based	Complete	[95]	110	10.483
377	*Sterna striata*	Laridae	Genetic-based	Absent	[71]	130	37.881
378	*Sterna vittata*	Laridae	Genetic-based	Absent	[71]	175	48.487
379	*Sterna paradisaea*	Laridae	Genetic-based	Complete	[5]	107	1.236
380	*Anous stolidus*	Laridae	Genetic-based	Complete	[9]	185	9.417
381	*Anous minutus*	Laridae	Genetic-based	Complete	[9]	110	2.165
382	*Anous albivitta*	Laridae	Birth-death polytomy resolution	Complete	[71]	55	25.736
383	*Rynchops niger*	Laridae	Genetic-based	Complete	[95]	370	1.225
384	*Rhodostethia rosea*	Laridae	Genetic-based	Absent	[95]	185	61.724
385	*Rissa brevirostris*	Laridae	Genetic-based	Absent	[9]	375	48.768
386	*Rissa tridactyla*	Laridae	Genetic-based	Absent	[5]	410	43.080
387	*Xema sabini*	Laridae	Genetic-based	Complete	[9]	190	19.624
388	*Pagophila eburnea*	Laridae	Genetic-based	Partial	[51]	610	67.422
389	*Hydrocoloeus minutus*	Laridae	Genetic-based	Absent	[5]	125	50.159
390	*Chroicocephalus genei*	Laridae	Genetic-based	Absent	[78]	290	31.315
391	*Chroicocephalus philadelphia*	Laridae	Genetic-based	Absent	[95]	210	43.705
392	*Chroicocephalus hartlaubii*	Laridae	Genetic-based	Complete	[18]	285	28.834
393	*Chroicocephalus cirrocephalus*	Laridae	Genetic-based	Complete	[73]	380	9.984
394	*Chroicocephalus ridibundus*	Laridae	Genetic-based	Absent	[5]	260	33.477
395	*Chroicocephalus brunnicephalus*	Laridae	Genetic-based	Partial	[71]	580	20.908
396	*Chroicocephalus bulleri*	Laridae	Genetic-based	Partial	[71]	230	41.284
397	*Chroicocephalus novaehollandiae*	Laridae	Genetic-based	Partial	[71]	285	31.711
398	*Leucophaeus atricilla*	Laridae	Genetic-based	Partial	[95]	315	10.501
399	*Leucophaeus pipixcan*	Laridae	Genetic-based	Partial	[9]	270	7.791

400	*Ichthyaetus melanocephalus*	Laridae	Genetic-based	Absent	5	280	38.331
401	*Larus canus*	Laridae	Genetic-based	Absent	5	420	45.882
402	*Larus heermanni*	Laridae	Genetic-based	Partial	95	510	35.547
403	*Larus delawarensis*	Laridae	Genetic-based	Absent	9	510	34.870
404	*Larus livens*	Laridae	Genetic-based	Absent	9	1200	28.316
405	*Larus occidentalis*	Laridae	Genetic-based	Absent	9	1000	36.412
406	*Larus hyperboreus*	Laridae	Genetic-based	Absent	9	1750	58.152
407	*Larus argentatus*	Laridae	Genetic-based	Absent	5	1000	56.153
408	*Larus michahellis*	Laridae	Genetic-based	Absent	5	960	38.468
409	*Larus marinus*	Laridae	Genetic-based	Absent	5	1850	54.780
410	*Larus dominicanus*	Laridae	Genetic-based	Absent	71	1050	36.262
411	*Larus fuscus*	Laridae	Genetic-based	Absent	5	875	24.571
412	*Larus californicus*	Laridae	Genetic-based	Absent	9	690	40.592
413	*Larus schistisagus*	Laridae	Genetic-based	Absent	95	1350	48.983
414	*Larus glaucescens*	Laridae	Genetic-based	Absent	9	1050	43.343
415	*Larus glaucoides*	Laridae	Genetic-based	Absent	9	950	55.929
416	*Geococcyx californianus*	Cuculidae	Genetic-based	Complete	89	300	29.504
417	*Crotophaga ani*	Cuculidae	Genetic-based	Complete	89	125	2.401
418	*Crotophaga sulcirostris*	Cuculidae	Genetic-based	Complete	89	75	3.295
419	*Piaya melanogaster*	Cuculidae	Genetic-based	Absent	56	102	3.346
420	*Coccyzus americanus*	Cuculidae	Genetic-based	Complete	89	61	6.557
421	*Coccyzus merlini*	Cuculidae	Genetic-based	Partial	92	160	21.547
422	*Urodynamis taitensis*	Cuculidae	Genetic-based	Absent	99	120	19.676
423	*Scythrops novaehollandiae*	Cuculidae	Genetic-based	Partial	71	610	16.552
424	*Chrysococcyx lucidus*	Cuculidae	Genetic-based	Complete	71	23	23.837
425	*Cuculus canorus*	Cuculidae	Genetic-based	Partial	5	116	18.204
426	*Cuculus optatus*	Cuculidae	Birth-death polytomy resolution	Partial	71	100	16.462
427	*Cacomantis flabelliformis*	Cuculidae	Genetic-based	Partial	71	50	22.110
428	*Cacomantis pallidus*	Cuculidae	Genetic-based	Partial	71	82	25.887
429	*Nyctibius aethereus*	Nyctibiidae	Genetic-based	Absent	56	365	9.595
430	*Nyctibius bracteatus*	Nyctibiidae	Genetic-based	Absent	56	52	2.474
431	*Cypseloides niger*	Apodidae	Genetic-based	Complete	87	40	32.981
432	*Chaetura pelagica*	Apodidae	Genetic-based	Absent	87	23.5	17.379
433	*Chaetura vauxi*	Apodidae	Genetic-based	Absent	87	19	31.780
434	*Aerodramus spodiopygius*	Apodidae	Genetic-based	Absent	98	7	12.017
435	*Aeronautes saxatalis*	Apodidae	Genetic-based	Absent	87	33.5	32.149
436	*Apus pacificus*	Apodidae	Genetic-based	Absent	71	38	10.962
437	*Apus apus*	Apodidae	Genetic-based	Absent	5	42	19.008
438	*Threnetes ruckeri*	Trochilidae	Genetic-based	Complete	134	6	6.484
439	*Glaucis aeneus*	Trochilidae	Genetic-based	Complete	134	4.5	7.630
440	*Phaethornis superciliosus*	Trochilidae	Genetic-based	Absent	56	5	0.908
441	*Phaethornis longirostris*	Trochilidae	Genetic-based	Complete	134	6	12.100
442	*Phaethornis striigularis*	Trochilidae	Genetic-based	Complete	134	2.5	7.886
443	*Phaethornis bourcieri*	Trochilidae	Genetic-based	Absent	56	4.5	2.147
444	*Florisuga mellivora*	Trochilidae	Genetic-based	Absent	56	8	0.072
445	*Topaza pella*	Trochilidae	Genetic-based	Absent	56	12.5	1.436
446	*Anthracothorax prevostii*	Trochilidae	Genetic-based	Complete	134	6.5	16.390
447	*Calothorax lucifer*	Trochilidae	Birth-death polytomy resolution	Complete	89	3	24.546
448	*Heliothryx auritus*	Trochilidae	Genetic-based	Complete	56	5	7.661
449	*Sephanoides sephaniodes*	Trochilidae	Genetic-based	Complete	97	5.5	41.997
450	*Patagona gigas*	Trochilidae	Genetic-based	Absent	97	19.5	18.806
451	*Lampornis clemenciae*	Trochilidae	Genetic-based	Complete	89	7	25.822
452	*Eugenes fulgens*	Trochilidae	Genetic-based	Complete	89	8.5	20.872
453	*Archilochus colubris*	Trochilidae	Genetic-based	Complete	91	3	31.355
454	*Archilochus alexandri*	Trochilidae	Genetic-based	Complete	91	3.5	34.134
455	*Calypte anna*	Trochilidae	Genetic-based	Complete	91	4.5	43.784
456	*Calypte costae*	Trochilidae	Genetic-based	Complete	91	3	29.574
457	*Selasphorus platycercus*	Trochilidae	Genetic-based	Complete	91	3.5	29.503
458	*Selasphorus sasin*	Trochilidae	Genetic-based	Complete	91	3.5	31.444
459	*Selasphorus calliope*	Trochilidae	Genetic-based	Complete	91	3.5	35.336
460	*Selasphorus rufus*	Trochilidae	Genetic-based	Complete	91	3.5	38.940
461	*Chlorostilbon ricordii*	Trochilidae	Birth-death polytomy resolution	Complete	92	4	23.427
462	*Cynanthus latirostris*	Trochilidae	Genetic-based	Complete	89	4	25.242
463	*Hylocharis xantusii*	Trochilidae	Genetic-based	Complete	9	3.5	25.682
464	*Hylocharis leucotis*	Trochilidae	Genetic-based	Complete	89	3.5	21.745
465	*Campylopterus largipennis*	Trochilidae	Genetic-based	Absent	56	8.5	5.651
466	*Microchera albocoronata*	Trochilidae	Genetic-based	Complete	119	2.5	12.243
467	*Thalurania colombica*	Trochilidae	Genetic-based	Complete	134	4.5	6.005

468	*Thalurania furcata*	Trochilidae	Genetic-based	Complete	[119]	4.5	8.496
469	*Amazilia yucatanensis*	Trochilidae	Genetic-based	Complete	[89]	4.5	23.862
470	*Amazilia tzacatl*	Trochilidae	Genetic-based	Complete	[119]	5	7.864
471	*Amazilia beryllina*	Trochilidae	Genetic-based	Complete	[89]	4	22.000
472	*Amazilia versicolor*	Trochilidae	Genetic-based	Complete	[56]	3.5	11.168
473	*Hylocharis eliciae*	Trochilidae	Genetic-based	Complete	[134]	3.5	12.976
474	*Amazilia amabilis*	Trochilidae	Genetic-based	Complete	[134]	4	5.100
475	*Hylocharis sapphirina*	Trochilidae	Genetic-based	Complete	[56]	4	9.862
476	*Amazilia violiceps*	Trochilidae	Birth-death polytomy resolution	Complete	[89]	5	24.819
477	*Amazilia fimbriata*	Trochilidae	Genetic-based	Complete	[56]	5	9.217
478	*Cathartes aura*	Cathartidae	Genetic-based	Absent	[95]	1850	0.846
479	*Gymnogyps californianus*	Cathartidae	Genetic-based	Absent	[117]	11000	39.814
480	*Coragyps atratus*	Cathartidae	Genetic-based	Absent	[95]	2000	1.945
481	*Pandion haliaetus*	Pandionidae	Genetic-based	Partial	[26]	1500	14.094
482	*Chelictinia riocourii*	Accipitridae	Birth-death polytomy resolution	Absent	[74]	100	8.448
483	*Elanus caeruleus*	Accipitridae	Genetic-based	Partial	[48]	260	5.907
484	*Elanus leucurus*	Accipitridae	Genetic-based	Absent	[94]	308	1.409
485	*Neophron percnopterus*	Accipitridae	Genetic-based	Partial	[141]	2000	7.186
486	*Gypaetus barbatus*	Accipitridae	Genetic-based	Absent	[140]	5800	8.337
487	*Chondrohierax uncinatus*	Accipitridae	Genetic-based	Absent	[95]	270	0.713
488	*Pernis apivorus*	Accipitridae	Genetic-based	Partial	[5]	700	16.111
489	*Elanoides forficatus*	Accipitridae	Genetic-based	Absent	[9]	465	5.183
490	*Circaetus gallicus*	Accipitridae	Genetic-based	Partial	[29]	1700	24.830
491	*Gyps africanus*	Accipitridae	Genetic-based	Absent	[50]	5450	5.842
492	*Gyps rueppelli*	Accipitridae	Genetic-based	Absent	[50]	7570	15.461
493	*Gyps fulvus*	Accipitridae	Genetic-based	Absent	[139]	8000	30.736
494	*Spizaetus ornatus*	Accipitridae	Genetic-based	Absent	[56]	1250	3.365
495	*Clanga pomarina*	Accipitridae	Genetic-based	Absent	[29]	1475	16.195
496	*Clanga clanga*	Accipitridae	Genetic-based	Absent	[29]	1925	33.602
497	*Hieraaetus morphnoides*	Accipitridae	Genetic-based	Absent	[21]	800	17.695
498	*Aquila chrysaetos*	Accipitridae	Genetic-based	Absent	[5]	4400	38.741
499	*Aquila heliaca*	Accipitridae	Genetic-based	Absent	[29]	3215	28.141
500	*Accipiter superciliosus*	Accipitridae	Genetic-based	Absent	[56]	95	6.752
501	*Accipiter gularis*	Accipitridae	Genetic-based	Partial	[60]	132	23.999
502	*Accipiter tachiro*	Accipitridae	Genetic-based	Absent	[69]	280	15.157
503	*Accipiter nisus*	Accipitridae	Genetic-based	Absent	[5]	210	32.084
504	*Accipiter melanoleucus*	Accipitridae	Birth-death polytomy resolution	Absent	[70]	640	8.001
505	*Accipiter striatus*	Accipitridae	Genetic-based	Absent	[94]	155	17.201
506	*Accipiter cooperii*	Accipitridae	Genetic-based	Absent	[94]	455	28.855
507	*Accipiter gentilis*	Accipitridae	Genetic-based	Absent	[5]	830	43.742
508	*Circus aeruginosus*	Accipitridae	Genetic-based	Absent	[5]	660	20.583
509	*Circus approximans*	Accipitridae	Birth-death polytomy resolution	Absent	[71]	750	30.817
510	*Circus cyaneus*	Accipitridae	Genetic-based	Absent	[5]	450	44.858
511	*Circus pygargus*	Accipitridae	Birth-death polytomy resolution	Absent	[36]	310	13.968
512	*Milvus migrans*	Accipitridae	Genetic-based	Absent	[5]	850	7.492
513	*Milvus milvus*	Accipitridae	Genetic-based	Absent	[5]	1140	38.229
514	*Haliaeetus albicilla*	Accipitridae	Genetic-based	Absent	[26]	4775	48.194
515	*Haliaeetus leucocephalus*	Accipitridae	Genetic-based	Absent	[94]	4650	43.920
516	*Haliaeetus leucogaster*	Accipitridae	Genetic-based	Absent	[71]	3300	8.752
517	*Haliaeetus vocifer*	Accipitridae	Genetic-based	Absent	[86]	2800	8.118
518	*Rostrhamus sociabilis*	Accipitridae	Genetic-based	Absent	[95]	430	3.987
519	*Ictinia mississippiensis*	Accipitridae	Genetic-based	Absent	[95]	280	6.840
520	*Buteogallus anthracinus*	Accipitridae	Genetic-based	Absent	[9]	1000	15.508
521	*Buteogallus urubitinga*	Accipitridae	Genetic-based	Partial	[9]	1150	3.515
522	*Parabuteo unicinctus*	Accipitridae	Genetic-based	Absent	[95]	850	4.777
523	*Pseudastur albicollis*	Accipitridae	Genetic-based	Absent	[56]	700	0.505
524	*Geranoaetus albicaudatus*	Accipitridae	Genetic-based	Absent	[95]	1000	5.263
525	*Leucopternis melanops*	Accipitridae	Genetic-based	Absent	[56]	330	2.149
526	*Buteo nitidus*	Accipitridae	Genetic-based	Absent	[9]	530	7.573
527	*Buteo lineatus*	Accipitridae	Genetic-based	Absent	[94]	625	33.110
528	*Buteo platypterus*	Accipitridae	Genetic-based	Absent	[94]	390	19.117
529	*Buteo swainsoni*	Accipitridae	Genetic-based	Absent	[94]	1050	14.048
530	*Buteo brachyurus*	Accipitridae	Genetic-based	Absent	[95]	440	0.301
531	*Buteo jamaicensis*	Accipitridae	Genetic-based	Absent	[94]	1100	38.463
532	*Buteo albonotatus*	Accipitridae	Genetic-based	Absent	[95]	750	5.009
533	*Buteo buteo*	Accipitridae	Genetic-based	Absent	[5]	860	16.519
534	*Buteo rufinus*	Accipitridae	Genetic-based	Absent	[29]	1150	24.708
535	*Buteo regalis*	Accipitridae	Genetic-based	Absent	[94]	1150	36.158

536	*Buteo lagopus*	Accipitridae	Genetic-based	Absent	95	1100	52.244
537	*Caprimulgus tristigma*	Caprimulgidae	Birth-death polytomy resolution	Complete	53	83	10.030
538	*Nyctipolus nigrescens*	Caprimulgidae	Genetic-based	Absent	56	37	3.044
539	*Eleothreptus candicans*	Caprimulgidae	Genetic-based	Complete	84	48.5	18.688
540	*Nyctidromus albicollis*	Caprimulgidae	Genetic-based	Absent	89	66	1.890
541	*Phalaenoptilus nuttallii*	Caprimulgidae	Genetic-based	Absent	89	46	36.652
542	*Antrostomus carolinensis*	Caprimulgidae	Genetic-based	Absent	89	116	21.809
543	*Antrostomus ridgwayi*	Caprimulgidae	Genetic-based	Absent	89	49	22.848
544	*Antrostomus vociferus*	Caprimulgidae	Genetic-based	Absent	89	53	31.350
545	*Chordeiles acutipennis*	Caprimulgidae	Genetic-based	Absent	89	49	7.868
546	*Chordeiles minor*	Caprimulgidae	Genetic-based	Partial	89	80	13.745
547	*Caprimulgus nubicus*	Caprimulgidae	Birth-death polytomy resolution	Complete	141	55	13.800
548	*Caprimulgus vexillarius*	Caprimulgidae	Genetic-based	Complete	53	73	6.661
549	*Caprimulgus fossii*	Caprimulgidae	Genetic-based	Complete	53	62	12.783
550	*Caprimulgus europaeus*	Caprimulgidae	Genetic-based	Complete	5	79	14.810
551	*Aegolius acadicus*	Strigidae	Genetic-based	Absent	114	104	39.714
552	*Aegolius funereus*	Strigidae	Genetic-based	Partial	49	130	51.192
553	*Athene cunicularia*	Strigidae	Genetic-based	Absent	122	167	0.173
554	*Athene noctua*	Strigidae	Genetic-based	Absent	5	170	31.587
555	*Surnia ulula*	Strigidae	Genetic-based	Absent	89	325	56.312
556	*Glaucidium siju*	Strigidae	Birth-death polytomy resolution	Absent	92	70	21.566
557	*Glaucidium gnoma*	Strigidae	Genetic-based	Absent	89	60	25.588
558	*Glaucidium brasilianum*	Strigidae	Genetic-based	Absent	89	75	0.773
559	*Glaucidium hardyi*	Strigidae	Genetic-based	Absent	56	60	4.729
560	*Asio otus*	Strigidae	Genetic-based	Absent	36	305	43.956
561	*Asio flammeus*	Strigidae	Genetic-based	Absent	36	334	9.598
562	*Psiloscops flammeolus*	Strigidae	Genetic-based	Absent	89	65	32.758
563	*Megascops trichopsis*	Strigidae	Genetic-based	Absent	89	95	22.329
564	*Megascops watsonii*	Strigidae	Genetic-based	Absent	56	130	3.335
565	*Megascops kennicottii*	Strigidae	Genetic-based	Absent	89	200	39.956
566	*Megascops asio*	Strigidae	Genetic-based	Absent	89	180	35.754
567	*Bubo virginianus*	Strigidae	Genetic-based	Absent	129	1625	16.359
568	*Bubo scandiacus*	Strigidae	Genetic-based	Absent	118	1700	53.748
569	*Strix aluco*	Strigidae	Genetic-based	Absent	36	497	47.263
570	*Strix nebulosa*	Strigidae	Genetic-based	Absent	89	1150	53.415
571	*Strix occidentalis*	Strigidae	Genetic-based	Absent	28	630	35.538
572	*Strix varia*	Strigidae	Genetic-based	Absent	89	715	38.542
573	*Tyto alba*	Tytonidae	Genetic-based	Absent	5	440	1.174
574	*Euptilotis neoxenus*	Trogonidae	Genetic-based	Absent	87	123	25.615
575	*Apaloderma vittatum*	Trogonidae	Genetic-based	Complete	24	55	4.393
576	*Trogon viridis*	Trogonidae	Genetic-based	Absent	56	90	8.051
577	*Trogon violaceus*	Trogonidae	Genetic-based	Absent	56	47.5	3.341
578	*Trogon elegans*	Trogonidae	Genetic-based	Absent	87	78	12.855
579	*Trogon rufus*	Trogonidae	Genetic-based	Absent	56	52.5	6.809
580	*Upupa epops*	Upupidae	Genetic-based	Complete	5	57	12.598
581	*Eurystomus orientalis*	Coraciidae	Genetic-based	Absent	71	123	8.067
582	*Todus multicolor*	Todidae	Genetic-based	Absent	9	5.9	21.566
583	*Momotus momota*	Momotidae	Genetic-based	Absent	56	125	7.524
584	*Alcedo atthis*	Alcedinidae	Genetic-based	Absent	5	30	25.111
585	*Todiramphus chloris*	Alcedinidae	Genetic-based	Absent	98	75	2.538
586	*Halcyon smyrnensis*	Alcedinidae	Birth-death polytomy resolution	Complete	141	99	15.665
587	*Halcyon leucocephala*	Alcedinidae	Genetic-based	Complete	58	42	3.427
588	*Ceryle rudis*	Alcedinidae	Genetic-based	Complete	141	92.5	2.053
589	*Megaceryle torquata*	Alcedinidae	Genetic-based	Absent	89	295	14.429
590	*Chloroceryle aenea*	Alcedinidae	Genetic-based	Partial	134	14	2.285
591	*Chloroceryle inda*	Alcedinidae	Genetic-based	Absent	56	54	7.677
592	*Chloroceryle americana*	Alcedinidae	Genetic-based	Partial	89	40	2.023
593	*Nyctyornis athertoni*	Meropidae	Birth-death polytomy resolution	Complete	32	85	19.464
594	*Merops bulocki*	Meropidae	Genetic-based	Complete	42	24.5	8.783
595	*Merops boehmi*	Meropidae	Genetic-based	Complete	42	17	10.924
596	*Merops pusillus*	Meropidae	Genetic-based	Complete	42	16	7.066
597	*Merops hirundineus*	Meropidae	Genetic-based	Complete	42	23.5	7.389
598	*Merops albicollis*	Meropidae	Genetic-based	Complete	32	26	5.897
599	*Merops nubicus*	Meropidae	Genetic-based	Complete	42	47	4.971
600	*Merops orientalis*	Meropidae	Genetic-based	Complete	32	17.5	20.281
601	*Merops apiaster*	Meropidae	Genetic-based	Complete	5	52	11.358
602	*Merops persicus*	Meropidae	Genetic-based	Complete	42	47	10.217
603	*Merops superciliosus*	Meropidae	Genetic-based	Complete	32	44	4.868

604	*Jacamerops aureus*	Galbulidae	Birth-death polytomy resolution	Partial	[56]	67	0.703
605	*Galbula albirostris*	Galbulidae	Genetic-based	Partial	[56]	20	2.037
606	*Galbula dea*	Galbulidae	Birth-death polytomy resolution	Partial	[56]	29	3.264
607	*Monasa atra*	Bucconidae	Birth-death polytomy resolution	Absent	[56]	89	2.896
608	*Bucco tamatia*	Bucconidae	Birth-death polytomy resolution	Absent	[56]	37.5	4.808
609	*Malacoptila fusca*	Bucconidae	Birth-death polytomy resolution	Absent	[56]	42	2.195
610	*Nonnula rubecula*	Bucconidae	Genetic-based	Absent	[56]	18.5	10.898
611	*Notharchus tectus*	Bucconidae	Birth-death polytomy resolution	Absent	[56]	30	3.371
612	*Trachyphonus vaillantii*	Lybiidae	Birth-death polytomy resolution	Absent	[46]	71	15.123
613	*Tricholaema leucomelas*	Lybiidae	Genetic-based	Absent	[46]	34	22.353
614	*Capito niger*	Capitonidae	Genetic-based	Absent	[56]	55	2.685
615	*Ramphastos vitellinus*	Ramphastidae	Genetic-based	Absent	[56]	350	3.753
616	*Selenidera piperivora*	Ramphastidae	Genetic-based	Absent	[56]	145	2.578
617	*Pteroglossus viridis*	Ramphastidae	Genetic-based	Absent	[56]	135	2.519
618	*Jynx torquilla*	Picidae	Genetic-based	Partial	[5]	33	34.942
619	*Picumnus exilis*	Picidae	Genetic-based	Absent	[56]	9	5.381
620	*Xiphidiopicus percussus*	Picidae	Genetic-based	Partial	[92]	73	21.547
621	*Campephilus rubricollis*	Picidae	Genetic-based	Partial	[56]	210	4.968
622	*Picus viridis*	Picidae	Genetic-based	Partial	[5]	194	49.540
623	*Celeus undatus*	Picidae	Birth-death polytomy resolution	Partial	[56]	65	1.733
624	*Colaptes auratus*	Picidae	Genetic-based	Partial	[88]	115	43.985
625	*Colaptes chrysoides*	Picidae	Birth-death polytomy resolution	Partial	[89]	110	29.113
626	*Celeus elegans*	Picidae	Genetic-based	Partial	[56]	135	3.195
627	*Dryocopus pileatus*	Picidae	Genetic-based	Partial	[88]	295	41.345
628	*Sphyrapicus thyroideus*	Picidae	Genetic-based	Partial	[88]	54	34.682
629	*Sphyrapicus varius*	Picidae	Genetic-based	Partial	[88]	50	36.011
630	*Sphyrapicus nuchalis*	Picidae	Genetic-based	Partial	[89]	52.5	37.702
631	*Sphyrapicus ruber*	Picidae	Genetic-based	Partial	[89]	47.5	45.484
632	*Melanerpes superciliaris*	Picidae	Birth-death polytomy resolution	Partial	[92]	85	23.092
633	*Melanerpes formicivorus*	Picidae	Genetic-based	Absent	[89]	78	23.579
634	*Melanerpes erythrocephalus*	Picidae	Genetic-based	Partial	[88]	77	38.669
635	*Melanerpes lewis*	Picidae	Genetic-based	Absent	[89]	110	41.974
636	*Melanerpes uropygialis*	Picidae	Genetic-based	Partial	[88]	66	28.078
637	*Melanerpes aurifrons*	Picidae	Genetic-based	Partial	[88]	81	24.563
638	*Melanerpes carolinus*	Picidae	Genetic-based	Partial	[88]	74	35.715
639	*Picoides tridactylus*	Picidae	Genetic-based	Partial	[88]	60	52.275
640	*Picoides arcticus*	Picidae	Genetic-based	Partial	[88]	75	52.160
641	*Dendrocopos syriacus*	Picidae	Genetic-based	Partial	[141]	68	40.155
642	*Dendrocopos major*	Picidae	Genetic-based	Partial	[5]	82	43.380
643	*Dryobates minor*	Picidae	Genetic-based	Partial	[5]	21	51.994
644	*Dryobates pubescens*	Picidae	Genetic-based	Partial	[88]	27	46.415
645	*Dryobates nuttallii*	Picidae	Genetic-based	Partial	[88]	37.5	36.846
646	*Dryobates scalaris*	Picidae	Genetic-based	Partial	[88]	33	25.797
647	*Dryobates lignarius*	Picidae	Genetic-based	Partial	[97]	37	34.972
648	*Dryobates cassini*	Picidae	Genetic-based	Absent	[56]	31	3.610
649	*Dryobates borealis*	Picidae	Genetic-based	Partial	[88]	47.5	32.079
650	*Dryobates villosus*	Picidae	Genetic-based	Partial	[88]	65	38.604
651	*Dryobates stricklandi*	Picidae	Genetic-based	Partial	[88]	60	19.104
652	*Dryobates albolarvatus*	Picidae	Genetic-based	Partial	[88]	60	41.547
653	*Micrastur mirandollei*	Falconidae	Birth-death polytomy resolution	Absent	[56]	475	4.429
654	*Micrastur gilvicollis*	Falconidae	Genetic-based	Absent	[56]	209	4.444
655	*Micrastur semitorquatus*	Falconidae	Genetic-based	Absent	[56]	680	2.771
656	*Micrastur ruficollis*	Falconidae	Birth-death polytomy resolution	Absent	[56]	210	5.373
657	*Caracara cheriway*	Falconidae	Birth-death polytomy resolution	Absent	[95]	1150	11.958
658	*Falco tinnunculus*	Falconidae	Genetic-based	Absent	[5]	183	18.174
659	*Falco naumanni*	Falconidae	Genetic-based	Absent	[29]	152	11.776
660	*Falco sparverius*	Falconidae	Genetic-based	Absent	[94]	115	7.188
661	*Falco columbarius*	Falconidae	Genetic-based	Absent	[5]	200	31.455
662	*Falco subbuteo*	Falconidae	Genetic-based	Absent	[5]	210	16.258
663	*Falco amurensis*	Falconidae	Genetic-based	Absent	[29]	138	11.290
664	*Falco vespertinus*	Falconidae	Genetic-based	Absent	[29]	158	17.573
665	*Falco femoralis*	Falconidae	Genetic-based	Absent	[95]	340	9.396
666	*Falco novaeseelandiae*	Falconidae	Genetic-based	Complete	[71]	425	44.253
667	*Falco mexicanus*	Falconidae	Genetic-based	Absent	[94]	700	36.952
668	*Falco peregrinus*	Falconidae	Genetic-based	Absent	[5]	915	12.103
669	*Falco rusticolus*	Falconidae	Genetic-based	Absent	[94]	1350	62.373
670	*Falco biarmicus*	Falconidae	Genetic-based	Absent	[29]	658	4.927
671	*Strigops habroptila*	Strigopidae	Genetic-based	Absent	[71]	1975	41.502

672	*Eolophus roseicapilla*	Cacatuidae	Genetic-based	Absent	71	310	27.435
673	*Myiopsitta monachus*	Psittacidae	Genetic-based	Absent	9	110	29.683
674	*Psittacula krameri*	Psittaculidae	Genetic-based	Complete	5	120	19.255
675	*Vini australis*	Psittaculidae	Genetic-based	Absent	98	50	17.340
676	*Cyanoramphus unicolor*	Psittaculidae	Genetic-based	Complete	71	130	49.690
677	*Platycercus elegans*	Psittaculidae	Genetic-based	Absent	71	130	27.712
678	*Platycercus eximius*	Psittaculidae	Genetic-based	Absent	135	105	34.262
679	*Xenicus gilviventris*	Acanthisittidae	Genetic-based	Absent	71	18	43.552
680	*Myrmornis torquata*	Thamnophilidae	Genetic-based	Absent	56	45	1.769
681	*Epinecrophylla gutturalis*	Thamnophilidae	Genetic-based	Absent	56	8.5	2.192
682	*Myrmophylax atrothorax*	Thamnophilidae	Genetic-based	Absent	56	16	5.038
683	*Isleria guttata*	Thamnophilidae	Birth-death polytomy resolution	Absent	56	10	2.380
684	*Myrmotherula menetriesii*	Thamnophilidae	Genetic-based	Absent	56	8.5	4.743
685	*Myrmotherula axillaris*	Thamnophilidae	Genetic-based	Absent	56	8	0.831
686	*Myrmotherula longipennis*	Thamnophilidae	Genetic-based	Absent	56	9	2.352
687	*Thamnomanes ardesiacus*	Thamnophilidae	Genetic-based	Absent	56	18.5	2.210
688	*Thamnomanes caesius*	Thamnophilidae	Genetic-based	Absent	56	17	7.124
689	*Cymbilaimus lineatus*	Thamnophilidae	Genetic-based	Partial	56	37.5	0.142
690	*Frederickena viridis*	Thamnophilidae	Genetic-based	Complete	56	70	2.396
691	*Dysithamnus mentalis*	Thamnophilidae	Genetic-based	Absent	133	13.5	5.791
692	*Thamnophilus doliatus*	Thamnophilidae	Genetic-based	Partial	134	27	1.860
693	*Thamnophilus atrinucha*	Thamnophilidae	Genetic-based	Absent	134	23	6.264
694	*Thamnophilus punctatus*	Thamnophilidae	Genetic-based	Absent	56	18.5	1.478
695	*Thamnophilus murinus*	Thamnophilidae	Genetic-based	Absent	56	18.5	2.423
696	*Myrmelastes leucostigma*	Thamnophilidae	Genetic-based	Absent	56	24	3.034
697	*Sclateria naevia*	Thamnophilidae	Genetic-based	Complete	107	24	2.570
698	*Poliocrania exsul*	Thamnophilidae	Genetic-based	Absent	134	29	11.543
699	*Hylophylax naevius*	Thamnophilidae	Genetic-based	Absent	56	12.5	5.092
700	*Percnostola rufifrons*	Thamnophilidae	Genetic-based	Complete	56	26.5	1.286
701	*Pyriglena leuconota*	Thamnophilidae	Genetic-based	Complete	107	31	9.486
702	*Pyriglena leucoptera*	Thamnophilidae	Genetic-based	Complete	107	29.5	21.657
703	*Cercomacroides tyrannina*	Thamnophilidae	Genetic-based	Absent	56	17	7.248
704	*Myrmoderus ferrugineus*	Thamnophilidae	Genetic-based	Absent	56	26.5	20.760
705	*Hypocnemis cantator*	Thamnophilidae	Genetic-based	Absent	56	12	2.406
706	*Willisornis poecilinotus*	Thamnophilidae	Genetic-based	Absent	56	18	5.003
707	*Pithys albifrons*	Thamnophilidae	Genetic-based	Complete	56	20.5	2.441
708	*Phlegopsis nigromaculata*	Thamnophilidae	Genetic-based	Complete	107	46.5	7.148
709	*Phlegopsis erythroptera*	Thamnophilidae	Genetic-based	Complete	107	54	4.212
710	*Gymnopithys rufigula*	Thamnophilidae	Genetic-based	Complete	56	29	2.356
711	*Gymnopithys leucaspis*	Thamnophilidae	Genetic-based	Complete	107	24	1.454
712	*Rhegmatorhina melanosticta*	Thamnophilidae	Genetic-based	Complete	107	31	6.668
713	*Rhegmatorhina hoffmannsi*	Thamnophilidae	Genetic-based	Complete	107	31	9.722
714	*Rhegmatorhina gymnops*	Thamnophilidae	Genetic-based	Complete	107	28.5	5.591
715	*Conopophaga aurita*	Conopophagidae	Genetic-based	Absent	56	22.5	2.015
716	*Grallaria varia*	Grallariidae	Genetic-based	Absent	56	115	11.848
717	*Myrmothera campanisona*	Grallariidae	Genetic-based	Absent	56	50	3.059
718	*Hylopezus macularius*	Grallariidae	Birth-death polytomy resolution	Absent	56	47	1.014
719	*Scytalopus fuscus*	Rhinocryptidae	Genetic-based	Absent	97	15	32.515
720	*Scelorchilus albicollis*	Rhinocryptidae	Birth-death polytomy resolution	Absent	97	55	26.095
721	*Pteroptochos megapodius*	Rhinocryptidae	Genetic-based	Absent	97	110	32.393
722	*Formicarius analis*	Formicariidae	Genetic-based	Complete	56	59	0.946
723	*Formicarius colma*	Formicariidae	Genetic-based	Absent	56	44	10.971
724	*Geositta cunicularia*	Furnariidae	Genetic-based	Absent	97	27	33.038
725	*Sclerurus mexicanus*	Furnariidae	Genetic-based	Complete	56	27	1.378
726	*Sclerurus rufigularis*	Furnariidae	Birth-death polytomy resolution	Complete	56	22	3.221
727	*Sclerurus caudacutus*	Furnariidae	Birth-death polytomy resolution	Complete	56	38	6.384
728	*Glyphorynchus spirurus*	Furnariidae	Genetic-based	Complete	56	16	0.419
729	*Dendrocincla fuliginosa*	Furnariidae	Genetic-based	Complete	56	38	0.836
730	*Dendrocincla merula*	Furnariidae	Genetic-based	Complete	56	41	4.828
731	*Sittasomus griseicapillus*	Furnariidae	Genetic-based	Complete	56	14	12.428
732	*Deconychura longicauda*	Furnariidae	Genetic-based	Complete	56	27	1.890
733	*Certhiasomus stictolaemus*	Furnariidae	Birth-death polytomy resolution	Complete	56	16.5	3.171
734	*Hylexetastes perrotii*	Furnariidae	Genetic-based	Complete	56	115	3.649
735	*Dendrocolaptes picumnus*	Furnariidae	Genetic-based	Complete	56	68	4.926
736	*Dendrocolaptes sanctithomae*	Furnariidae	Genetic-based	Complete	134	70	9.698
737	*Dendrocolaptes certhia*	Furnariidae	Genetic-based	Complete	56	64	4.049
738	*Xiphorhynchus flavigaster*	Furnariidae	Genetic-based	Complete	37	48	19.179
739	*Xiphorhynchus pardalotus*	Furnariidae	Genetic-based	Complete	56	39	2.558

740	*Campylorhamphus procurvoides*	Furnariidae	Genetic-based	Complete	[56]	34	0.289
741	*Lepidocolaptes leucogaster*	Furnariidae	Genetic-based	Complete	[22]	35	22.444
742	*Lepidocolaptes souleyetii*	Furnariidae	Genetic-based	Complete	[9]	27	5.813
743	*Lepidocolaptes albolineatus*	Furnariidae	Genetic-based	Complete	[56]	20	3.611
744	*Xenops minutus*	Furnariidae	Genetic-based	Complete	[56]	8.5	19.010
745	*Upucerthia saturatior*	Furnariidae	Birth-death polytomy resolution	Absent	[97]	50	40.268
746	*Aphrastura spinicauda*	Furnariidae	Genetic-based	Absent	[97]	11.5	43.461
747	*Leptasthenura aegithaloides*	Furnariidae	Genetic-based	Absent	[97]	9.5	28.982
748	*Pseudasthenes humicola*	Furnariidae	Genetic-based	Absent	[97]	21	31.311
749	*Synallaxis rutilans*	Furnariidae	Genetic-based	Absent	[56]	18.5	3.803
750	*Synallaxis erythrothorax*	Furnariidae	Genetic-based	Complete	[22]	17	17.371
751	*Syndactyla subalaris*	Furnariidae	Birth-death polytomy resolution	Complete	[107]	33	0.989
752	*Philydor pyrrhodes*	Furnariidae	Genetic-based	Complete	[56]	30	3.532
753	*Philydor erythrocercum*	Furnariidae	Birth-death polytomy resolution	Complete	[56]	24.5	5.078
754	*Automolus rufipileatus*	Furnariidae	Genetic-based	Complete	[107]	34.5	4.204
755	*Automolus melanopezus*	Furnariidae	Genetic-based	Complete	[107]	29.5	6.286
756	*Clibanornis rubiginosus*	Furnariidae	Genetic-based	Complete	[56]	37	3.322
757	*Automolus infuscatus*	Furnariidae	Genetic-based	Complete	[56]	33	3.031
758	*Automolus ochrolaemus*	Furnariidae	Genetic-based	Complete	[56]	38	0.604
759	*Automolus subulatus*	Furnariidae	Genetic-based	Complete	[107]	28	4.225
760	*Automolus virgatus*	Furnariidae	Birth-death polytomy resolution	Complete	[107]	32.5	5.946
761	*Phytotoma rara*	Cotingidae	Genetic-based	Partial	[97]	41	40.336
762	*Lipaugus vociferans*	Cotingidae	Genetic-based	Complete	[56]	69	5.146
763	*Phoenicircus carnifex*	Cotingidae	Birth-death polytomy resolution	Absent	[56]	89	1.184
764	*Neopelma chrysocephalum*	Pipridae	Genetic-based	Absent	[56]	15.5	1.774
765	*Tyranneutes virescens*	Pipridae	Genetic-based	Absent	[56]	7.5	2.570
766	*Chiroxiphia lanceolata*	Pipridae	Birth-death polytomy resolution	Absent	[25]	16.5	8.364
767	*Corapipo gutturalis*	Pipridae	Genetic-based	Absent	[56]	8.5	2.210
768	*Lepidothrix coronata*	Pipridae	Genetic-based	Absent	[106]	8.5	3.808
769	*Lepidothrix serena*	Pipridae	Genetic-based	Absent	[56]	11	1.894
770	*Manacus candei*	Pipridae	Genetic-based	Absent	[134]	20	13.922
771	*Manacus manacus*	Pipridae	Genetic-based	Absent	[56]	16	8.203
772	*Pipra filicauda*	Pipridae	Genetic-based	Absent	[106]	14	1.468
773	*Dixiphia pipra*	Pipridae	Genetic-based	Absent	[56]	11.5	5.968
774	*Ceratopipra erythrocephala*	Pipridae	Genetic-based	Absent	[56]	12.5	2.513
775	*Ceratopipra mentalis*	Pipridae	Genetic-based	Absent	[134]	15	8.606
776	*Onychorhynchus coronatus*	Oxyruncidae	Genetic-based	Absent	[56]	15	3.051
777	*Myiobius barbatus*	Oxyruncidae	Genetic-based	Complete	[56]	11	9.588
778	*Terenotriccus erythrurus*	Oxyruncidae	Genetic-based	Complete	[56]	6.5	0.098
779	*Laniocera hypopyrra*	Tityridae	Genetic-based	Complete	[56]	46	4.842
780	*Tityra cayana*	Tityridae	Genetic-based	Absent	[56]	70	1.049
781	*Pachyramphus marginatus*	Tityridae	Genetic-based	Complete	[56]	18	8.303
782	*Pachyramphus aglaiae*	Tityridae	Genetic-based	Absent	[90]	29.5	19.618
783	*Pachyramphus minor*	Tityridae	Genetic-based	Absent	[56]	37	4.880
784	*Piprites chloris*	Tyrannidae	Genetic-based	Absent	[56]	18	8.285
785	*Corythopis torquatus*	Tyrannidae	Genetic-based	Absent	[56]	16.5	3.378
786	*Mionectes oleagineus*	Tyrannidae	Genetic-based	Absent	[56]	11.5	0.794
787	*Mionectes macconnelli*	Tyrannidae	Genetic-based	Absent	[56]	13	4.566
788	*Mionectes olivaceus*	Tyrannidae	Genetic-based	Absent	[133]	14.5	9.493
789	*Mionectes striaticollis*	Tyrannidae	Genetic-based	Absent	[62]	15	5.392
790	*Rhynchocyclus olivaceus*	Tyrannidae	Genetic-based	Complete	[56]	21.5	6.826
791	*Tolmomyias assimilis*	Tyrannidae	Genetic-based	Complete	[56]	15	4.634
792	*Tolmomyias poliocephalus*	Tyrannidae	Genetic-based	Complete	[56]	11	6.298
793	*Tolmomyias sulphurescens*	Tyrannidae	Genetic-based	Complete	[22]	15	4.675
794	*Todirostrum cinereum*	Tyrannidae	Genetic-based	Complete	[22]	6	2.389
795	*Hemitriccus josephinae*	Tyrannidae	Genetic-based	Complete	[56]	10.5	2.426
796	*Hemitriccus zosterops*	Tyrannidae	Genetic-based	Complete	[56]	9	2.295
797	*Lophotriccus galeatus*	Tyrannidae	Genetic-based	Complete	[56]	6.5	0.339
798	*Neopipo cinnamomea*	Tyrannidae	Genetic-based	Complete	[56]	7	3.137
799	*Platyrinchus saturatus*	Tyrannidae	Genetic-based	Absent	[56]	10.5	2.284
800	*Platyrinchus coronatus*	Tyrannidae	Genetic-based	Absent	[56]	8.5	0.137
801	*Platyrinchus platyrhynchos*	Tyrannidae	Genetic-based	Absent	[56]	12	4.106
802	*Camptostoma imberbe*	Tyrannidae	Genetic-based	Complete	[89]	7	21.424
803	*Myiopagis viridicata*	Tyrannidae	Genetic-based	Absent	[37]	12	2.382
804	*Anairetes parulus*	Tyrannidae	Genetic-based	Partial	[97]	6	26.925
805	*Elaenia parvirostris*	Tyrannidae	Genetic-based	Partial	[56]	23	12.451
806	*Elaenia albiceps*	Tyrannidae	Genetic-based	Partial	[97]	16	27.064
807	*Ramphotrigon ruficauda*	Tyrannidae	Genetic-based	Partial	[56]	20	3.122

808	*Attila spadiceus*	Tyrannidae	Genetic-based	Partial	56	38	2.019
809	*Rhytipterna simplex*	Tyrannidae	Genetic-based	Partial	56	35	7.469
810	*Myiarchus ferox*	Tyrannidae	Genetic-based	Partial	56	28	9.963
811	*Myiarchus swainsoni*	Tyrannidae	Genetic-based	Partial	56	26.5	15.150
812	*Myiarchus tuberculifer*	Tyrannidae	Genetic-based	Partial	56	21	4.006
813	*Myiarchus cinerascens*	Tyrannidae	Genetic-based	Absent	90	27.5	29.813
814	*Myiarchus crinitus*	Tyrannidae	Genetic-based	Absent	90	33.5	28.692
815	*Myiarchus tyrannulus*	Tyrannidae	Genetic-based	Partial	90	34	2.473
816	*Pitangus sulphuratus*	Tyrannidae	Genetic-based	Absent	90	57.5	7.552
817	*Myiodynastes luteiventris*	Tyrannidae	Genetic-based	Partial	90	45	7.531
818	*Tyrannus tyrannus*	Tyrannidae	Genetic-based	Partial	89	41	18.512
819	*Tyrannus vociferans*	Tyrannidae	Genetic-based	Absent	90	43	30.719
820	*Tyrannus forficatus*	Tyrannidae	Genetic-based	Partial	90	39.5	23.893
821	*Tyrannus verticalis*	Tyrannidae	Genetic-based	Partial	90	38	31.272
822	*Tyrannus savana*	Tyrannidae	Genetic-based	Absent	89	30	12.224
823	*Tyrannus crassirostris*	Tyrannidae	Genetic-based	Absent	90	55	24.276
824	*Tyrannus melancholicus*	Tyrannidae	Genetic-based	Partial	90	38.5	3.935
825	*Tyrannus couchii*	Tyrannidae	Genetic-based	Partial	90	40	22.511
826	*Tyrannus dominicensis*	Tyrannidae	Genetic-based	Partial	89	45.5	16.644
827	*Pyrocephalus rubinus*	Tyrannidae	Genetic-based	Partial	90	13.5	0.842
828	*Muscisaxicola maclovianus*	Tyrannidae	Genetic-based	Complete	97	20	31.464
829	*Xolmis pyrope*	Tyrannidae	Genetic-based	Absent	97	35	39.884
830	*Contopus pertinax*	Tyrannidae	Genetic-based	Absent	90	27	23.999
831	*Mitrephanes phaeocercus*	Tyrannidae	Genetic-based	Absent	37	8.5	15.106
832	*Sayornis saya*	Tyrannidae	Genetic-based	Absent	90	20	43.354
833	*Sayornis phoebe*	Tyrannidae	Genetic-based	Absent	90	18.5	41.384
834	*Sayornis nigricans*	Tyrannidae	Genetic-based	Absent	90	18	7.077
835	*Contopus sordidulus*	Tyrannidae	Genetic-based	Partial	89	13	25.306
836	*Contopus virens*	Tyrannidae	Genetic-based	Partial	89	14	18.383
837	*Contopus caribaeus*	Tyrannidae	Birth-death polytomy resolution	Absent	92	11	23.427
838	*Contopus cooperi*	Tyrannidae	Genetic-based	Partial	90	34.5	21.356
839	*Empidonax virescens*	Tyrannidae	Genetic-based	Absent	90	13	21.256
840	*Empidonax flaviventris*	Tyrannidae	Genetic-based	Partial	90	12.5	35.629
841	*Empidonax difficilis*	Tyrannidae	Genetic-based	Absent	90	10.5	37.910
842	*Empidonax occidentalis*	Tyrannidae	Genetic-based	Absent	90	10.5	36.538
843	*Empidonax traillii*	Tyrannidae	Genetic-based	Partial	90	14	26.240
844	*Empidonax fulvifrons*	Tyrannidae	Genetic-based	Absent	90	8	23.885
845	*Empidonax wrightii*	Tyrannidae	Genetic-based	Absent	90	12.5	33.377
846	*Empidonax minimus*	Tyrannidae	Genetic-based	Absent	90	10.5	37.447
847	*Empidonax oberholseri*	Tyrannidae	Genetic-based	Absent	90	10.5	38.026
848	*Empidonax hammondii*	Tyrannidae	Genetic-based	Absent	90	10	40.105
849	*Atrichornis clamosus*	Atrichornithidae	Genetic-based	Absent	116	45	34.021
850	*Climacteris picumnus*	Climacteridae	Genetic-based	Complete	71	33	24.342
851	*Ptilonorhynchus violaceus*	Ptilonorhynchidae	Genetic-based	Complete	127	223	27.000
852	*Malurus leucopterus*	Maluridae	Genetic-based	Complete	71	7	27.411
853	*Malurus lamberti*	Maluridae	Genetic-based	Complete	71	8	29.221
854	*Malurus splendens*	Maluridae	Genetic-based	Complete	71	9.5	27.837
855	*Malurus cyaneus*	Maluridae	Genetic-based	Complete	71	11.5	33.416
856	*Gerygone albofrontata*	Acanthizidae	Birth-death polytomy resolution	Absent	71	10	44.029
857	*Pyrrholaemus sagittatus*	Acanthizidae	Genetic-based	Absent	34	13.5	30.598
858	*Phylidonyris pyrrhopterus*	Meliphagidae	Genetic-based	Complete	71	17.5	37.171
859	*Foulehaio carunculatus*	Meliphagidae	Genetic-based	Absent	98	31	17.340
860	*Sugomel nigrum*	Meliphagidae	Genetic-based	Complete	71	10.5	26.264
861	*Myzomela rubratra*	Meliphagidae	Birth-death polytomy resolution	Partial	100	14	12.644
862	*Myzomela erythrocephala*	Meliphagidae	Genetic-based	Complete	68	8.5	11.327
863	*Myzomela cardinalis*	Meliphagidae	Genetic-based	Partial	98	14	16.115
864	*Gliciphila melanops*	Meliphagidae	Genetic-based	Complete	71	17.5	35.400
865	*Ramsayornis modestus*	Meliphagidae	Genetic-based	Complete	71	10.5	10.010
866	*Prosthemadera novaeseelandiae*	Meliphagidae	Genetic-based	Absent	71	95	42.690
867	*Certhionyx variegatus*	Meliphagidae	Genetic-based	Complete	71	27	28.179
868	*Gavicalis versicolor*	Meliphagidae	Birth-death polytomy resolution	Complete	71	34	9.637
869	*Anthornis melanura*	Meliphagidae	Birth-death polytomy resolution	Absent	71	27.5	42.954
870	*Anthochaera carunculata*	Meliphagidae	Genetic-based	Absent	71	110	33.013
871	*Gavicalis fasciogularis*	Meliphagidae	Birth-death polytomy resolution	Complete	71	27	24.727
872	*Philesturnus rufusater*	Callaeidae	Genetic-based	Absent	71	70	38.401
873	*Notiomystis cincta*	Notiomystidae	Genetic-based	Partial	115	33	38.383
874	*Coracina novaehollandiae*	Campephagidae	Genetic-based	Absent	71	120	20.503
875	*Lalage tricolor*	Campephagidae	Genetic-based	Absent	71	29	23.932

876	*Artamus superciliosus*	Artamidae	Genetic-based	Absent	71	35	27.054
877	*Batis molitor*	Platysteiridae	Genetic-based	Absent	46	9.5	14.639
878	*Tchagra senegalus*	Malaconotidae	Genetic-based	Complete	22	50	1.794
879	*Laniarius atrococcineus*	Malaconotidae	Genetic-based	Absent	46	48	21.971
880	*Rhodophoneus cruentus*	Malaconotidae	Genetic-based	Partial	111	47	8.178
881	*Cyclarhis gujanensis*	Vireonidae	Genetic-based	Complete	56	28.5	5.924
882	*Pachysylvia decurtata*	Vireonidae	Genetic-based	Absent	134	8.5	8.646
883	*Tunchiornis ochraceiceps*	Vireonidae	Genetic-based	Complete	56	11	1.804
884	*Pachysylvia muscicapina*	Vireonidae	Birth-death polytomy resolution	Complete	56	11.5	4.057
885	*Vireo philadelphicus*	Vireonidae	Genetic-based	Absent	90	13	33.352
886	*Vireo hypochryseus*	Vireonidae	Birth-death polytomy resolution	Absent	37	12	22.033
887	*Vireo gilvus*	Vireonidae	Genetic-based	Absent	90	14	39.785
888	*Vireo olivaceus*	Vireonidae	Genetic-based	Absent	90	18.5	16.118
889	*Vireo flavoviridis*	Vireonidae	Genetic-based	Absent	89	18.5	5.780
890	*Vireo altiloquus*	Vireonidae	Genetic-based	Absent	89	21	9.819
891	*Vireo huttoni*	Vireonidae	Genetic-based	Absent	90	12	32.567
892	*Vireo vicinior*	Vireonidae	Genetic-based	Absent	90	12.5	31.673
893	*Vireo solitarius*	Vireonidae	Genetic-based	Absent	90	16.5	35.659
894	*Vireo flavifrons*	Vireonidae	Genetic-based	Absent	90	18	26.338
895	*Vireo gundlachii*	Vireonidae	Birth-death polytomy resolution	Absent	92	13	21.566
896	*Vireo atricapilla*	Vireonidae	Genetic-based	Absent	90	9	26.610
897	*Vireo bellii*	Vireonidae	Genetic-based	Absent	90	8.5	30.250
898	*Vireo nelsoni*	Vireonidae	Birth-death polytomy resolution	Absent	37	9.5	18.605
899	*Vireo griseus*	Vireonidae	Genetic-based	Partial	90	12	29.002
900	*Oriolus oriolus*	Oriolidae	Genetic-based	Partial	54	72	14.417
901	*Dicrurus adsimilis*	Dicruridae	Genetic-based	Absent	46	46	8.751
902	*Rhipidura leucophrys*	Rhipiduridae	Genetic-based	Partial	71	18	-18.234
903	*Rhipidura fuliginosa*	Rhipiduridae	Genetic-based	Absent	71	8	40.883
904	*Rhipidura rufifrons*	Rhipiduridae	Genetic-based	Absent	100	11	12.097
905	*Terpsiphone viridis*	Monarchidae	Genetic-based	Absent	111	13.5	7.791
906	*Myiagra cyanoleuca*	Monarchidae	Genetic-based	Absent	71	17.5	22.451
907	*Monarcha melanopsis*	Monarchidae	Genetic-based	Absent	71	23	22.350
908	*Clytorhynchus vitiensis*	Monarchidae	Birth-death polytomy resolution	Absent	98	29	16.973
909	*Chasiempis sandwichensis*	Monarchidae	Genetic-based	Complete	31	14.5	19.627
910	*Lanius souzae*	Laniidae	Birth-death polytomy resolution	Complete	22	25.5	10.138
911	*Lanius meridionalis*	Laniidae	Genetic-based	Partial	111	60	40.449
912	*Lanius excubitor*	Laniidae	Genetic-based	Partial	141	62	38.474
913	*Lanius ludovicianus*	Laniidae	Genetic-based	Partial	90	48.5	35.581
914	*Lanius nubicus*	Laniidae	Genetic-based	Absent	121	24.5	25.126
915	*Lanius isabellinus*	Laniidae	Genetic-based	Partial	66	29	22.157
916	*Lanius collurio*	Laniidae	Genetic-based	Complete	121	28	16.091
917	*Lanius schach*	Laniidae	Genetic-based	Absent	111	45	16.684
918	*Lanius cristatus*	Laniidae	Genetic-based	Partial	121	31.5	30.805
919	*Lanius collaris*	Laniidae	Genetic-based	Complete	15	42	9.067
920	*Lanius senator*	Laniidae	Genetic-based	Partial	54	40	24.732
921	*Lanius minor*	Laniidae	Genetic-based	Complete	121	51.5	12.253
922	*Corvinella melanoleuca*	Laniidae	Birth-death polytomy resolution	Complete	125	80	15.201
923	*Pyrrhocorax graculus*	Corvidae	Genetic-based	Absent	111	220	40.065
924	*Pyrrhocorax pyrrhocorax*	Corvidae	Genetic-based	Absent	36	291	31.494
925	*Cyanopica cyanus*	Corvidae	Genetic-based	Absent	20	96.5	40.878
926	*Perisoreus infaustus*	Corvidae	Genetic-based	Absent	121	87	57.020
927	*Perisoreus canadensis*	Corvidae	Genetic-based	Absent	90	67.5	51.284
928	*Psilorhinus morio*	Corvidae	Genetic-based	Absent	90	199	17.898
929	*Cyanocorax yncas*	Corvidae	Genetic-based	Absent	90	79	5.300
930	*Aphelocoma ultramarina*	Corvidae	Genetic-based	Absent	90	133	19.864
931	*Aphelocoma californica*	Corvidae	Genetic-based	Absent	90	85	31.489
932	*Aphelocoma coerulescens*	Corvidae	Genetic-based	Absent	90	77	27.999
933	*Gymnorhinus cyanocephalus*	Corvidae	Genetic-based	Absent	90	105	38.440
934	*Cyanocitta stelleri*	Corvidae	Genetic-based	Absent	90	121	37.226
935	*Cyanocitta cristata*	Corvidae	Genetic-based	Absent	90	85	40.642
936	*Garrulus glandarius*	Corvidae	Genetic-based	Absent	54	158	49.466
937	*Podoces pleskei*	Corvidae	Birth-death polytomy resolution	Absent	111	88	31.663
938	*Pica pica*	Corvidae	Genetic-based	Absent	54	223	43.081
939	*Pica hudsonia*	Corvidae	Genetic-based	Absent	9	175	50.208
940	*Pica nuttalli*	Corvidae	Genetic-based	Absent	90	156	37.678
941	*Nucifraga caryocatactes*	Corvidae	Genetic-based	Absent	54	172	51.012
942	*Nucifraga columbiana*	Corvidae	Genetic-based	Absent	90	134	39.535
943	*Corvus ossifragus*	Corvidae	Genetic-based	Absent	90	273	34.728

944	*Corvus sinaloae*	Corvidae	Birth-death polytomy resolution	Absent	[9]	245	24.405
945	*Corvus monedula*	Corvidae	Genetic-based	Absent	[121]	200	47.985
946	*Corvus mellori*	Corvidae	Birth-death polytomy resolution	Absent	[71]	520	34.464
947	*Corvus dauuricus*	Corvidae	Genetic-based	Absent	[17]	190	38.558
948	*Corvus splendens*	Corvidae	Birth-death polytomy resolution	Absent	[17]	310	17.612
949	*Corvus tasmanicus*	Corvidae	Birth-death polytomy resolution	Absent	[71]	650	36.149
950	*Corvus macrorhynchos*	Corvidae	Genetic-based	Absent	[136]	730	22.362
951	*Corvus ruficollis*	Corvidae	Birth-death polytomy resolution	Absent	[17]	570	29.420
952	*Corvus frugilegus*	Corvidae	Genetic-based	Absent	[36]	450	43.148
953	*Corvus rhipidurus*	Corvidae	Birth-death polytomy resolution	Absent	[17]	625	15.646
954	*Corvus cryptoleucus*	Corvidae	Genetic-based	Absent	[90]	525	29.249
955	*Corvus corax*	Corvidae	Genetic-based	Absent	[36]	1300	47.937
956	*Corvus albus*	Corvidae	Genetic-based	Absent	[17]	550	7.550
957	*Corvus imparatus*	Corvidae	Birth-death polytomy resolution	Absent	[9]	220	24.035
958	*Corvus hawaiiensis*	Corvidae	Genetic-based	Absent	[9]	500	19.312
959	*Corvus coronoides*	Corvidae	Genetic-based	Absent	[71]	660	25.838
960	*Corvus orru*	Corvidae	Genetic-based	Absent	[71]	550	15.434
961	*Corvus bennetti*	Corvidae	Birth-death polytomy resolution	Absent	[71]	400	25.358
962	*Corvus corone*	Corvidae	Genetic-based	Absent	[141]	500	45.709
963	*Corvus brachyrhynchos*	Corvidae	Genetic-based	Absent	[90]	413	44.136
964	*Corvus caurinus*	Corvidae	Genetic-based	Absent	[90]	392	53.878
965	*Petroica australis*	Petroicidae	Genetic-based	Partial	[71]	35	44.003
966	*Petroica macrocephala*	Petroicidae	Genetic-based	Partial	[71]	11	42.723
967	*Elminia albonotata*	Stenostiridae	Genetic-based	Absent	[24]	7.5	10.100
968	*Remiz pendulinus*	Remizidae	Genetic-based	Absent	[121]	10	42.316
969	*Auriparus flaviceps*	Remizidae	Genetic-based	Partial	[90]	7	28.308
970	*Cyanistes caeruleus*	Paridae	Genetic-based	Absent	[121]	11	48.840
971	*Cyanistes cyanus*	Paridae	Genetic-based	Absent	[111]	13.5	47.771
972	*Cyanistes teneriffae*	Paridae	Birth-death polytomy resolution	Absent	[111]	10	32.527
973	*Poecile palustris*	Paridae	Genetic-based	Absent	[121]	12	48.572
974	*Parus major*	Paridae	Genetic-based	Absent	[54]	17	30.289
975	*Lophophanes cristatus*	Paridae	Genetic-based	Absent	[121]	13	51.838
976	*Baeolophus wollweberi*	Paridae	Genetic-based	Absent	[90]	10.5	25.317
977	*Baeolophus bicolor*	Paridae	Genetic-based	Absent	[90]	22	35.802
978	*Baeolophus inornatus*	Paridae	Genetic-based	Absent	[90]	16	32.935
979	*Periparus ater*	Paridae	Genetic-based	Absent	[54]	9.5	46.000
980	*Periparus venustulus*	Paridae	Genetic-based	Absent	[141]	10.5	31.923
981	*Poecile lugubris*	Paridae	Genetic-based	Absent	[111]	17	37.881
982	*Poecile gambeli*	Paridae	Genetic-based	Absent	[90]	11	45.468
983	*Poecile atricapillus*	Paridae	Genetic-based	Absent	[90]	11.5	50.085
984	*Poecile sclateri*	Paridae	Genetic-based	Absent	[90]	9.5	24.145
985	*Poecile carolinensis*	Paridae	Genetic-based	Absent	[90]	10.5	34.448
986	*Poecile cinctus*	Paridae	Genetic-based	Absent	[121]	12.5	60.401
987	*Poecile rufescens*	Paridae	Genetic-based	Absent	[90]	10.5	47.925
988	*Poecile hudsonicus*	Paridae	Genetic-based	Absent	[90]	10	56.310
989	*Panurus biarmicus*	Panuridae	Genetic-based	Complete	[121]	16	49.076
990	*Ammomanes cinctura*	Alaudidae	Genetic-based	Complete	[17]	18.5	25.513
991	*Ammomanes phoenicura*	Alaudidae	Genetic-based	Complete	[141]	23	20.208
992	*Eremopterix griseus*	Alaudidae	Birth-death polytomy resolution	Complete	[141]	16	20.369
993	*Eremopterix leucopareia*	Alaudidae	Birth-death polytomy resolution	Complete	[141]	16	5.025
994	*Eremopterix nigriceps*	Alaudidae	Genetic-based	Complete	[17]	14	17.009
995	*Eremopterix signatus*	Alaudidae	Birth-death polytomy resolution	Complete	[111]	16	3.502
996	*Alaemon alaudipes*	Alaudidae	Genetic-based	Complete	[17]	43	23.288
997	*Alaemon hamertoni*	Alaudidae	Birth-death polytomy resolution	Complete	[17]	39	6.958
998	*Calendulauda albescens*	Alaudidae	Genetic-based	Complete	[132]	28.5	31.151
999	*Ammomanes deserti*	Alaudidae	Birth-death polytomy resolution	Complete	[17]	25	25.756
1000	*Ammomanopsis grayi*	Alaudidae	Genetic-based	Complete	[132]	21.5	21.190
1001	*Ramphocoris clotbey*	Alaudidae	Birth-death polytomy resolution	Complete	[17]	50	28.301
1002	*Chersomanes albofasciata*	Alaudidae	Genetic-based	Complete	[132]	26	22.786
1003	*Mirafra javanica*	Alaudidae	Genetic-based	Complete	[71]	23	4.413
1004	*Mirafra cantillans*	Alaudidae	Birth-death polytomy resolution	Complete	[111]	18	13.338
1005	*Chersophilus duponti*	Alaudidae	Genetic-based	Complete	[17]	39.5	36.288
1006	*Eremalauda dunni*	Alaudidae	Genetic-based	Complete	[17]	20	19.098
1007	*Spizocorys starki*	Alaudidae	Genetic-based	Complete	[15]	19	20.722
1008	*Lullula arborea*	Alaudidae	Genetic-based	Complete	[121]	29	45.928
1009	*Galerida deva*	Alaudidae	Birth-death polytomy resolution	Complete	[17]	27	19.274
1010	*Galerida cristata*	Alaudidae	Genetic-based	Complete	[121]	42.5	30.588
1011	*Galerida theklae*	Alaudidae	Genetic-based	Complete	[121]	35	21.586

1012	*Alauda razae*	Alaudidae	Birth-death polytomy resolution	Complete	17	22	16.616
1013	*Alauda gulgula*	Alaudidae	Genetic-based	Complete	121	27	26.177
1014	*Alauda arvensis*	Alaudidae	Genetic-based	Complete	121	38	44.098
1015	*Alaudala cheleensis*	Alaudidae	Genetic-based	Complete	17	23.5	41.466
1016	*Calandrella brachydactyla*	Alaudidae	Genetic-based	Complete	121	21	28.085
1017	*Calandrella acutirostris*	Alaudidae	Genetic-based	Complete	111	21	33.099
1018	*Alaudala raytal*	Alaudidae	Birth-death polytomy resolution	Complete	111	21	24.917
1019	*Alaudala rufescens*	Alaudidae	Genetic-based	Complete	17	23.5	37.402
1020	*Eremophila bilopha*	Alaudidae	Genetic-based	Complete	121	23	28.367
1021	*Eremophila alpestris*	Alaudidae	Genetic-based	Complete	121	35	40.894
1022	*Melanocorypha bimaculata*	Alaudidae	Genetic-based	Complete	121	55	30.657
1023	*Melanocorypha yeltoniensis*	Alaudidae	Birth-death polytomy resolution	Complete	121	63	45.274
1024	*Melanocorypha maxima*	Alaudidae	Birth-death polytomy resolution	Complete	121	56	33.972
1025	*Melanocorypha calandra*	Alaudidae	Genetic-based	Complete	121	59	38.671
1026	*Alauda leucoptera*	Alaudidae	Birth-death polytomy resolution	Complete	141	44	46.819
1027	*Melanocorypha mongolica*	Alaudidae	Genetic-based	Complete	141	55	41.264
1028	*Andropadus importunus*	Pycnonotidae	Genetic-based	Complete	10	28.5	15.157
1029	*Phyllastrephus cabanisi*	Pycnonotidae	Genetic-based	Complete	125	28.5	5.971
1030	*Phyllastrephus terrestris*	Pycnonotidae	Genetic-based	Complete	41	40	16.283
1031	*Alophoixus bres*	Pycnonotidae	Genetic-based	Complete	27	32	7.328
1032	*Alophoixus phaeocephalus*	Pycnonotidae	Genetic-based	Complete	27	31.5	2.320
1033	*Hypsipetes amaurotis*	Pycnonotidae	Genetic-based	Complete	61	75	33.580
1034	*Iole propinqua*	Pycnonotidae	Genetic-based	Complete	83	26	16.088
1035	*Brachypodius atriceps*	Pycnonotidae	Genetic-based	Complete	141	25	10.015
1036	*Brachypodius eutilotus*	Pycnonotidae	Genetic-based	Complete	27	36.5	4.299
1037	*Pycnonotus striatus*	Pycnonotidae	Birth-death polytomy resolution	Complete	141	52.5	23.201
1038	*Pycnonotus leucotis*	Pycnonotidae	Birth-death polytomy resolution	Complete	111	27.5	25.178
1039	*Pycnonotus xanthopygos*	Pycnonotidae	Birth-death polytomy resolution	Complete	43	37	25.045
1040	*Rubigula melanicterus*	Pycnonotidae	Genetic-based	Complete	141	34	7.873
1041	*Pycnonotus finlaysoni*	Pycnonotidae	Genetic-based	Complete	59	28.5	13.492
1042	*Pycnonotus cafer*	Pycnonotidae	Birth-death polytomy resolution	Complete	35	34	20.887
1043	*Pycnonotus luteolus*	Pycnonotidae	Birth-death polytomy resolution	Complete	141	35.5	15.388
1044	*Pycnonotus flavescens*	Pycnonotidae	Genetic-based	Complete	141	31	19.679
1045	*Pycnonotus xantholaemus*	Pycnonotidae	Birth-death polytomy resolution	Complete	141	36	14.592
1046	*Pycnonotus sinensis*	Pycnonotidae	Genetic-based	Complete	22	38	29.917
1047	*Pycnonotus barbatus*	Pycnonotidae	Genetic-based	Complete	15	39	1.887
1048	*Pycnonotus leucogenys*	Pycnonotidae	Genetic-based	Complete	17	36	31.272
1049	*Pycnonotus jocosus*	Pycnonotidae	Genetic-based	Complete	71	36	17.152
1050	*Pycnonotus goiavier*	Pycnonotidae	Genetic-based	Complete	128	30.5	4.794
1051	*Cisticola juncidis*	Cisticolidae	Genetic-based	Complete	141	8	8.124
1052	*Cisticola tinniens*	Cisticolidae	Birth-death polytomy resolution	Complete	15	11.5	17.117
1053	*Cisticola chiniana*	Cisticolidae	Birth-death polytomy resolution	Absent	46	15	7.549
1054	*Eremomela usticollis*	Cisticolidae	Birth-death polytomy resolution	Absent	46	8	19.316
1055	*Spiloptila clamans*	Cisticolidae	Genetic-based	Partial	111	7	16.734
1056	*Prinia rufescens*	Cisticolidae	Birth-death polytomy resolution	Complete	4	6.5	15.740
1057	*Prinia flavicans*	Cisticolidae	Birth-death polytomy resolution	Complete	47	9	22.691
1058	*Prinia buchanani*	Cisticolidae	Birth-death polytomy resolution	Complete	4	7	25.877
1059	*Prinia inornata*	Cisticolidae	Birth-death polytomy resolution	Complete	4	7.5	13.011
1060	*Prinia flaviventris*	Cisticolidae	Birth-death polytomy resolution	Complete	4	9	13.083
1061	*Prinia polychroa*	Cisticolidae	Birth-death polytomy resolution	Complete	4	15	7.971
1062	*Prinia gracilis*	Cisticolidae	Birth-death polytomy resolution	Partial	64	7	20.034
1063	*Prinia sylvatica*	Cisticolidae	Birth-death polytomy resolution	Complete	4	16	19.061
1064	*Prinia subflava*	Cisticolidae	Genetic-based	Complete	125	9.5	8.164
1065	*Zosterops conspicillatus*	Zosteropidae	Genetic-based	Complete	100	9	13.450
1066	*Zosterops senegalensis*	Zosteropidae	Genetic-based	Complete	24	10.5	5.737
1067	*Stachyris nigricollis*	Timaliidae	Birth-death polytomy resolution	Complete	27	24.5	2.315
1068	*Cleptornis marchei*	Zosteropidae	Genetic-based	Absent	100	20	15.066
1069	*Stachyris poliocephala*	Timaliidae	Genetic-based	Complete	27	27	1.297
1070	*Macronus ptilosus*	Timaliidae	Genetic-based	Complete	27	26	3.299
1071	*Malacopteron magnum*	Pellorneidae	Birth-death polytomy resolution	Complete	27	28	2.851
1072	*Pellorneum malaccense*	Pellorneidae	Genetic-based	Complete	27	23	1.737
1073	*Pellorneum capistratum*	Pellorneidae	Genetic-based	Complete	27	31	7.344
1074	*Leiothrix lutea*	Leiothrichidae	Genetic-based	Complete	22	21	29.280
1075	*Turdoides striata*	Leiothrichidae	Genetic-based	Complete	35	70	21.535
1076	*Turdoides leucopygia*	Leiothrichidae	Birth-death polytomy resolution	Complete	125	78	10.694
1077	*Turdoides altirostris*	Leiothrichidae	Birth-death polytomy resolution	Complete	17	33	33.846
1078	*Turdoides caudata*	Leiothrichidae	Birth-death polytomy resolution	Complete	35	35	22.679
1079	*Chamaea fasciata*	Sylviidae	Genetic-based	Complete	89	14.5	38.104

1080	*Chrysomma sinense*	Sylviidae	Genetic-based	Complete	[35]	22	20.411
1081	*Sylvia borin*	Sylviidae	Genetic-based	Complete	[54]	17.5	19.130
1082	*Sylvia atricapilla*	Sylviidae	Genetic-based	Absent	[54]	17	26.917
1083	*Sylvia nana*	Sylviidae	Genetic-based	Absent	[110]	9	29.494
1084	*Sylvia curruca*	Sylviidae	Genetic-based	Absent	[54]	11.5	37.680
1085	*Sylvia leucomelaena*	Sylviidae	Genetic-based	Partial	[110]	14	20.175
1086	*Sylvia buryi*	Sylviidae	Genetic-based	Absent	[111]	22	16.466
1087	*Sylvia melanothorax*	Sylviidae	Genetic-based	Partial	[110]	11	25.867
1088	*Sylvia ruppeli*	Sylviidae	Genetic-based	Absent	[110]	12	25.982
1089	*Sylvia cantillans*	Sylviidae	Genetic-based	Absent	[110]	10.5	28.517
1090	*Sylvia mystacea*	Sylviidae	Genetic-based	Absent	[110]	10	27.959
1091	*Sylvia melanocephala*	Sylviidae	Genetic-based	Partial	[110]	10	30.591
1092	*Sylvia conspicillata*	Sylviidae	Genetic-based	Absent	[110]	8.5	29.591
1093	*Sylvia communis*	Sylviidae	Genetic-based	Partial	[54]	14	20.791
1094	*Sylvia sarda*	Sylviidae	Genetic-based	Absent	[110]	10.5	37.438
1095	*Sylvia undata*	Sylviidae	Genetic-based	Absent	[110]	8.5	41.357
1096	*Sylvia deserticola*	Sylviidae	Genetic-based	Absent	[110]	8.5	27.057
1097	*Acrocephalus palustris*	Acrocephalidae	Genetic-based	Complete	[63]	12	15.821
1098	*Locustella pryeri*	Locustellidae	Genetic-based	Complete	[63]	13	38.029
1099	*Locustella fasciolata*	Locustellidae	Genetic-based	Complete	[121]	27.5	24.044
1100	*Locustella certhiola*	Locustellidae	Genetic-based	Complete	[121]	15.5	28.612
1101	*Locustella pleskei*	Locustellidae	Genetic-based	Complete	[63]	20	31.617
1102	*Locustella ochotensis*	Locustellidae	Genetic-based	Complete	[63]	21	31.871
1103	*Locustella tacsanowskia*	Locustellidae	Genetic-based	Complete	[63]	11	32.734
1104	*Locustella naevia*	Locustellidae	Genetic-based	Partial	[54]	14	36.489
1105	*Locustella lanceolata*	Locustellidae	Genetic-based	Complete	[121]	11	30.540
1106	*Locustella fluviatilis*	Locustellidae	Genetic-based	Complete	[54]	16	19.232
1107	*Locustella luscinioides*	Locustellidae	Genetic-based	Partial	[63]	16.5	33.051
1108	*Scotocerca inquieta*	Scotocercidae	Genetic-based	Complete	[141]	8	29.487
1109	*Hippolais polyglotta*	Acrocephalidae	Genetic-based	Complete	[63]	11	27.274
1110	*Hippolais icterina*	Acrocephalidae	Genetic-based	Complete	[63]	13.5	17.993
1111	*Hippolais languida*	Acrocephalidae	Genetic-based	Complete	[63]	15	17.318
1112	*Hippolais olivetorum*	Acrocephalidae	Genetic-based	Complete	[63]	18.5	7.318
1113	*Acrocephalus luscinius*	Acrocephalidae	Birth-death polytomy resolution	Complete	[100]	34	13.447
1114	*Arundinax aedon*	Acrocephalidae	Genetic-based	Partial	[63]	26.5	33.687
1115	*Iduna pallida*	Acrocephalidae	Genetic-based	Complete	[63]	12	20.715
1116	*Iduna opaca*	Acrocephalidae	Genetic-based	Complete	[111]	13	23.628
1117	*Iduna caligata*	Acrocephalidae	Genetic-based	Complete	[63]	9	35.831
1118	*Iduna rama*	Acrocephalidae	Genetic-based	Complete	[63]	9	27.358
1119	*Acrocephalus griseldis*	Acrocephalidae	Genetic-based	Complete	[63]	17	4.459
1120	*Acrocephalus gracilirostris*	Acrocephalidae	Genetic-based	Complete	[63]	15	10.033
1121	*Acrocephalus arundinaceus*	Acrocephalidae	Genetic-based	Complete	[63]	27	15.378
1122	*Acrocephalus australis*	Acrocephalidae	Genetic-based	Absent	[63]	20	12.324
1123	*Acrocephalus stentoreus*	Acrocephalidae	Genetic-based	Complete	[63]	29	26.601
1124	*Acrocephalus bistrigiceps*	Acrocephalidae	Genetic-based	Complete	[63]	9.5	28.011
1125	*Acrocephalus paludicola*	Acrocephalidae	Genetic-based	Complete	[63]	12.5	35.566
1126	*Acrocephalus schoenobaenus*	Acrocephalidae	Genetic-based	Complete	[63]	11	18.669
1127	*Acrocephalus melanopogon*	Acrocephalidae	Genetic-based	Complete	[63]	10.5	36.870
1128	*Acrocephalus agricola*	Acrocephalidae	Genetic-based	Complete	[63]	10.5	33.402
1129	*Acrocephalus tangorum*	Acrocephalidae	Genetic-based	Complete	[63]	8	31.869
1130	*Acrocephalus concinens*	Acrocephalidae	Genetic-based	Complete	[63]	8.5	27.348
1131	*Acrocephalus dumetorum*	Acrocephalidae	Genetic-based	Complete	[63]	11	35.072
1132	*Acrocephalus scirpaceus*	Acrocephalidae	Genetic-based	Complete	[63]	12	15.127
1133	*Acrocephalus baeticatus*	Acrocephalidae	Genetic-based	Complete	[63]	10.5	7.870
1134	*Aegithalos caudatus*	Aegithalidae	Genetic-based	Complete	[121]	8.5	49.261
1135	*Psaltriparus minimus*	Aegithalidae	Genetic-based	Complete	[89]	5.5	32.239
1136	*Horornis diphone*	Scotocercidae	Genetic-based	Complete	[1]	15	35.434
1137	*Cettia cetti*	Scotocercidae	Genetic-based	Absent	[63]	13.5	38.773
1138	*Urosphena squameiceps*	Scotocercidae	Genetic-based	Complete	[63]	9	32.148
1139	*Cettia castaneocoronata*	Scotocercidae	Birth-death polytomy resolution	Complete	[63]	9	24.572
1140	*Horornis canturians*	Scotocercidae	Birth-death polytomy resolution	Complete	[63]	19.5	31.419
1141	*Phylloscopus trochiloides*	Phylloscopidae	Genetic-based	Complete	[111]	8.5	37.340
1142	*Phylloscopus nitidus*	Phylloscopidae	Genetic-based	Complete	[111]	7.5	25.386
1143	*Phylloscopus ibericus*	Phylloscopidae	Birth-death polytomy resolution	Absent	[111]	7.5	26.857
1144	*Phylloscopus neglectus*	Phylloscopidae	Birth-death polytomy resolution	Absent	[111]	5.5	30.490
1145	*Phylloscopus borealis*	Phylloscopidae	Genetic-based	Complete	[121]	9.5	30.855
1146	*Phylloscopus umbrovirens*	Phylloscopidae	Genetic-based	Absent	[111]	10	7.389
1147	*Phylloscopus sibilatrix*	Phylloscopidae	Genetic-based	Complete	[121]	10.5	31.865

1148	*Phylloscopus orientalis*	Phylloscopidae	Genetic-based	Complete	121	8	27.421
1149	*Phylloscopus bonelli*	Phylloscopidae	Genetic-based	Complete	121	8	29.753
1150	*Phylloscopus proregulus*	Phylloscopidae	Genetic-based	Absent	121	5	39.506
1151	*Phylloscopus humei*	Phylloscopidae	Genetic-based	Absent	121	7	34.350
1152	*Phylloscopus inornatus*	Phylloscopidae	Genetic-based	Absent	121	5.5	35.994
1153	*Phylloscopus schwarzi*	Phylloscopidae	Genetic-based	Absent	111	10	34.721
1154	*Phylloscopus fuscatus*	Phylloscopidae	Genetic-based	Absent	121	8	34.022
1155	*Phylloscopus trochilus*	Phylloscopidae	Genetic-based	Complete	121	10	18.738
1156	*Phylloscopus canariensis*	Phylloscopidae	Genetic-based	Absent	111	8	28.214
1157	*Phylloscopus collybita*	Phylloscopidae	Genetic-based	Absent	121	8	34.308
1158	*Atticora tibialis*	Hirundinidae	Genetic-based	Complete	56	10	7.536
1159	*Stelgidopteryx ruficollis*	Hirundinidae	Genetic-based	Complete	123	16	10.189
1160	*Stelgidopteryx serripennis*	Hirundinidae	Genetic-based	Complete	137	14.5	31.816
1161	*Progne tapera*	Hirundinidae	Genetic-based	Complete	123	35	13.288
1162	*Progne chalybea*	Hirundinidae	Genetic-based	Complete	141	41	6.749
1163	*Progne subis*	Hirundinidae	Genetic-based	Complete	76	55	10.097
1164	*Riparia paludicola*	Hirundinidae	Genetic-based	Complete	17	12.5	0.150
1165	*Riparia riparia*	Hirundinidae	Genetic-based	Complete	121	15	16.398
1166	*Tachycineta thalassina*	Hirundinidae	Genetic-based	Complete	89	15	39.650
1167	*Tachycineta bicolor*	Hirundinidae	Genetic-based	Complete	120	21	34.731
1168	*Tachycineta albiventer*	Hirundinidae	Genetic-based	Complete	123	15.5	8.849
1169	*Tachycineta albilinea*	Hirundinidae	Genetic-based	Complete	22	14	18.196
1170	*Tachycineta meyeni*	Hirundinidae	Genetic-based	Complete	123	17.5	35.780
1171	*Tachycineta leucorrhoa*	Hirundinidae	Genetic-based	Complete	141	19	22.840
1172	*Pseudhirundo griseopyga*	Hirundinidae	Genetic-based	Complete	141	10	7.376
1173	*Psalidoprocne pristoptera*	Hirundinidae	Genetic-based	Complete	141	12	9.051
1174	*Psalidoprocne albiceps*	Hirundinidae	Genetic-based	Complete	141	12.5	4.111
1175	*Delichon dasypus*	Hirundinidae	Genetic-based	Complete	71	18	23.374
1176	*Delichon urbicum*	Hirundinidae	Genetic-based	Complete	17	16.5	17.737
1177	*Ptyonoprogne rupestris*	Hirundinidae	Genetic-based	Complete	17	25	29.398
1178	*Hirundo neoxena*	Hirundinidae	Genetic-based	Complete	71	15	30.720
1179	*Hirundo smithii*	Hirundinidae	Genetic-based	Complete	111	13	4.478
1180	*Hirundo rustica*	Hirundinidae	Genetic-based	Complete	17	20	7.707
1181	*Hirundo aethiopica*	Hirundinidae	Genetic-based	Complete	123	13.5	4.080
1182	*Hirundo angolensis*	Hirundinidae	Genetic-based	Complete	123	17.5	6.350
1183	*Cecropis semirufa*	Hirundinidae	Genetic-based	Complete	123	32.5	7.509
1184	*Cecropis senegalensis*	Hirundinidae	Birth-death polytomy resolution	Complete	123	46	4.421
1185	*Cecropis daurica*	Hirundinidae	Genetic-based	Complete	17	25	17.130
1186	*Cecropis abyssinica*	Hirundinidae	Genetic-based	Complete	123	21.5	8.920
1187	*Petrochelidon fulva*	Hirundinidae	Genetic-based	Complete	89	16	23.242
1188	*Petrochelidon pyrrhonota*	Hirundinidae	Genetic-based	Complete	89	22	14.825
1189	*Petrochelidon rufigula*	Hirundinidae	Genetic-based	Complete	141	16	8.544
1190	*Petrochelidon nigricans*	Hirundinidae	Genetic-based	Complete	71	16.5	21.940
1191	*Petrochelidon ariel*	Hirundinidae	Genetic-based	Complete	71	11.5	25.261
1192	*Regulus satrapa*	Regulidae	Genetic-based	Absent	90	5.5	38.537
1193	*Regulus ignicapilla*	Regulidae	Genetic-based	Absent	17	6	43.197
1194	*Regulus regulus*	Regulidae	Genetic-based	Absent	121	6	46.108
1195	*Regulus calendula*	Regulidae	Genetic-based	Absent	90	6.5	42.927
1196	*Phainopepla nitens*	Ptiliogonatidae	Genetic-based	Partial	90	22	29.083
1197	*Hypocolius ampelinus*	Hypocoliidae	Genetic-based	Absent	111	53	25.903
1198	*Bombycilla garrulus*	Bombycillidae	Genetic-based	Absent	54	55	50.721
1199	*Bombycilla cedrorum*	Bombycillidae	Genetic-based	Absent	90	33	31.908
1200	*Sitta neumayer*	Sittidae	Genetic-based	Absent	121	27	38.502
1201	*Sitta tephronota*	Sittidae	Genetic-based	Absent	17	49	36.252
1202	*Sitta carolinensis*	Sittidae	Genetic-based	Absent	90	21	38.206
1203	*Sitta europaea*	Sittidae	Genetic-based	Absent	121	22.5	44.825
1204	*Sitta pygmaea*	Sittidae	Genetic-based	Absent	90	10.5	34.615
1205	*Sitta pusilla*	Sittidae	Genetic-based	Absent	90	10	32.305
1206	*Sitta whiteheadi*	Sittidae	Genetic-based	Absent	121	13	42.042
1207	*Sitta canadensis*	Sittidae	Genetic-based	Absent	90	10.5	45.450
1208	*Sitta krueperi*	Sittidae	Genetic-based	Absent	121	12	40.295
1209	*Sitta ledanti*	Sittidae	Genetic-based	Absent	17	17.5	36.719
1210	*Tichodroma muraria*	Tichodromidae	Genetic-based	Absent	9	17.5	36.668
1211	*Certhia brachydactyla*	Certhiidae	Genetic-based	Absent	121	9.5	43.495
1212	*Certhia americana*	Certhiidae	Genetic-based	Absent	90	9	38.084
1213	*Certhia familiaris*	Certhiidae	Genetic-based	Absent	121	9	51.104
1214	*Ramphocaenus melanurus*	Polioptilidae	Genetic-based	Absent	56	9.5	3.026
1215	*Microbates collaris*	Polioptilidae	Birth-death polytomy resolution	Absent	56	11	0.011

1216	*Polioptila lembeyei*	Polioptilidae	Birth-death polytomy resolution	Partial	92	5	21.193
1217	*Polioptila caerulea*	Polioptilidae	Genetic-based	Absent	90	6	30.226
1218	*Polioptila melanura*	Polioptilidae	Genetic-based	Absent	90	5.5	29.346
1219	*Polioptila californica*	Polioptilidae	Genetic-based	Absent	90	5.5	28.524
1220	*Salpinctes obsoletus*	Troglodytidae	Genetic-based	Absent	90	16.5	32.491
1221	*Catherpes mexicanus*	Troglodytidae	Genetic-based	Absent	90	13.5	33.830
1222	*Microcerculus bambla*	Troglodytidae	Birth-death polytomy resolution	Absent	56	18	2.598
1223	*Campylorhynchus brunneicapillus*	Troglodytidae	Genetic-based	Partial	90	40	29.000
1224	*Cistothorus palustris*	Troglodytidae	Genetic-based	Partial	90	11.5	37.645
1225	*Cistothorus platensis*	Troglodytidae	Genetic-based	Absent	90	9	17.052
1226	*Troglodytes aedon*	Troglodytidae	Genetic-based	Absent	90	11	1.970
1227	*Troglodytes troglodytes*	Troglodytidae	Genetic-based	Absent	121	9	43.745
1228	*Cyphorhinus arada*	Troglodytidae	Genetic-based	Absent	56	21	4.322
1229	*Cinnycerthia fulva*	Troglodytidae	Birth-death polytomy resolution	Absent	62	16.5	14.353
1230	*Thryomanes bewickii*	Troglodytidae	Genetic-based	Absent	90	10	33.340
1231	*Henicorhina leucosticta*	Troglodytidae	Genetic-based	Absent	105	15.5	5.901
1232	*Thryothorus ludovicianus*	Troglodytidae	Genetic-based	Partial	90	18.5	33.545
1233	*Pheugopedius maculipectus*	Troglodytidae	Genetic-based	Absent	105	15	18.084
1234	*Pheugopedius coraya*	Troglodytidae	Genetic-based	Absent	56	24	2.139
1235	*Cantorchilus nigricapillus*	Troglodytidae	Genetic-based	Absent	134	22	5.474
1236	*Thryophilus rufalbus*	Troglodytidae	Genetic-based	Absent	133	26.5	9.438
1237	*Buphagus erythrorynchus*	Buphagidae	Genetic-based	Complete	15	51	8.119
1238	*Dumetella carolinensis*	Mimidae	Genetic-based	Absent	90	39.5	31.128
1239	*Oreoscoptes montanus*	Mimidae	Genetic-based	Absent	90	45	36.023
1240	*Mimus polyglottos*	Mimidae	Genetic-based	Absent	90	48.5	33.599
1241	*Mimus thenca*	Mimidae	Genetic-based	Absent	97	66	34.776
1242	*Toxostoma curvirostre*	Mimidae	Genetic-based	Absent	90	79.5	27.338
1243	*Toxostoma longirostre*	Mimidae	Genetic-based	Absent	90	68	24.484
1244	*Toxostoma rufum*	Mimidae	Genetic-based	Absent	90	69	39.324
1245	*Toxostoma bendirei*	Mimidae	Genetic-based	Absent	90	60	31.966
1246	*Toxostoma redivivum*	Mimidae	Genetic-based	Absent	90	84.5	35.709
1247	*Toxostoma crissale*	Mimidae	Genetic-based	Partial	90	63	28.479
1248	*Toxostoma lecontei*	Mimidae	Genetic-based	Absent	90	62	31.763
1249	*Aplonis opaca*	Sturnidae	Birth-death polytomy resolution	Absent	100	82	10.276
1250	*Aplonis atrifusca*	Sturnidae	Birth-death polytomy resolution	Absent	98	142	13.907
1251	*Aplonis tabuensis*	Sturnidae	Genetic-based	Absent	98	61	15.647
1252	*Onychognathus tristramii*	Sturnidae	Genetic-based	Absent	43	123	23.209
1253	*Lamprotornis pulcher*	Sturnidae	Genetic-based	Complete	130	68	14.617
1254	*Lamprotornis mevesii*	Sturnidae	Genetic-based	Complete	15	71	17.189
1255	*Creatophora cinerea*	Sturnidae	Genetic-based	Complete	16	75	9.347
1256	*Sturnus vulgaris*	Sturnidae	Genetic-based	Complete	17	78	46.969
1257	*Sturnus unicolor*	Sturnidae	Genetic-based	Complete	121	85	36.419
1258	*Pastor roseus*	Sturnidae	Genetic-based	Complete	17	74	29.115
1259	*Gracupica contra*	Sturnidae	Genetic-based	Complete	104	83	22.389
1260	*Agropsar sturninus*	Sturnidae	Genetic-based	Complete	17	100	22.631
1261	*Agropsar philippensis*	Sturnidae	Genetic-based	Complete	136	50	26.102
1262	*Sturnia malabarica*	Sturnidae	Genetic-based	Complete	111	38	20.462
1263	*Sturnia pagodarum*	Sturnidae	Genetic-based	Complete	111	47	21.525
1264	*Spodiopsar cineraceus*	Sturnidae	Genetic-based	Complete	22	82	37.709
1265	*Acridotheres fuscus*	Sturnidae	Genetic-based	Complete	99	85	18.278
1266	*Acridotheres cristatellus*	Sturnidae	Genetic-based	Complete	9	108	22.905
1267	*Acridotheres tristis*	Sturnidae	Genetic-based	Complete	15	113	24.550
1268	*Acridotheres ginginianus*	Sturnidae	Genetic-based	Complete	111	70	26.329
1269	*Cinclus cinclus*	Cinclidae	Genetic-based	Absent	54	60	48.219
1270	*Cinclus mexicanus*	Cinclidae	Genetic-based	Absent	90	57	38.985
1271	*Sialia sialis*	Turdidae	Genetic-based	Absent	90	29	33.415
1272	*Sialia mexicana*	Turdidae	Genetic-based	Absent	90	27.5	35.718
1273	*Sialia currucoides*	Turdidae	Genetic-based	Absent	90	30	43.202
1274	*Myadestes townsendi*	Turdidae	Genetic-based	Absent	90	32.5	44.303
1275	*Myadestes obscurus*	Turdidae	Genetic-based	Absent	9	51	19.608
1276	*Myadestes occidentalis*	Turdidae	Genetic-based	Absent	37	41	20.983
1277	*Myadestes melanops*	Turdidae	Genetic-based	Absent	133	31.5	9.670
1278	*Myadestes ralloides*	Turdidae	Genetic-based	Absent	44	31	5.018
1279	*Zoothera dauma*	Turdidae	Genetic-based	Absent	17	110	21.193
1280	*Zoothera heinei*	Turdidae	Genetic-based	Absent	71	100	17.415
1281	*Zoothera lunulata*	Turdidae	Genetic-based	Absent	71	105	30.346
1282	*Ixoreus naevius*	Turdidae	Genetic-based	Absent	90	83	49.908
1283	*Hylocichla mustelina*	Turdidae	Genetic-based	Absent	90	56	28.143

1284	*Catharus fuscater*	Turdidae	Genetic-based	Absent	[133]	36.5	3.484
1285	*Catharus dryas*	Turdidae	Genetic-based	Absent	[38]	40	15.313
1286	*Catharus aurantiirostris*	Turdidae	Genetic-based	Absent	[133]	26.5	15.242
1287	*Catharus mexicanus*	Turdidae	Genetic-based	Absent	[38]	30	15.981
1288	*Catharus ustulatus*	Turdidae	Genetic-based	Absent	[90]	35	32.035
1289	*Catharus occidentalis*	Turdidae	Genetic-based	Absent	[37]	26	21.881
1290	*Catharus guttatus*	Turdidae	Genetic-based	Absent	[90]	27.5	39.976
1291	*Catharus frantzii*	Turdidae	Genetic-based	Absent	[37]	31	15.102
1292	*Catharus bicknelli*	Turdidae	Genetic-based	Absent	[90]	29.5	33.169
1293	*Catharus fuscescens*	Turdidae	Genetic-based	Absent	[90]	34	14.956
1294	*Catharus minimus*	Turdidae	Genetic-based	Absent	[90]	38	28.818
1295	*Geokichla sibirica*	Turdidae	Genetic-based	Absent	[17]	66	30.014
1296	*Turdus viscivorus*	Turdidae	Genetic-based	Absent	[17]	130	48.734
1297	*Turdus philomelos*	Turdidae	Genetic-based	Absent	[17]	68	40.602
1298	*Turdus menachensis*	Turdidae	Genetic-based	Absent	[111]	75	17.513
1299	*Turdus iliacus*	Turdidae	Genetic-based	Absent	[17]	63	49.714
1300	*Turdus olivaceus*	Turdidae	Genetic-based	Absent	[24]	80	24.509
1301	*Turdus merula*	Turdidae	Genetic-based	Absent	[17]	88	48.252
1302	*Turdus cardis*	Turdidae	Genetic-based	Absent	[136]	60	30.178
1303	*Turdus obscurus*	Turdidae	Genetic-based	Absent	[17]	89	30.858
1304	*Turdus pallidus*	Turdidae	Genetic-based	Absent	[17]	77	37.152
1305	*Turdus celaenops*	Turdidae	Genetic-based	Absent	[136]	75	32.111
1306	*Turdus chrysolaus*	Turdidae	Genetic-based	Absent	[136]	77	33.311
1307	*Turdus pilaris*	Turdidae	Genetic-based	Absent	[17]	111	48.559
1308	*Turdus torquatus*	Turdidae	Genetic-based	Absent	[17]	114	49.213
1309	*Turdus naumanni*	Turdidae	Genetic-based	Absent	[17]	72	42.875
1310	*Turdus atrogularis*	Turdidae	Genetic-based	Absent	[17]	82	41.088
1311	*Turdus libonyana*	Turdidae	Genetic-based	Absent	[46]	64	16.240
1312	*Turdus migratorius*	Turdidae	Genetic-based	Absent	[90]	77	43.248
1313	*Turdus falcklandii*	Turdidae	Genetic-based	Absent	[97]	105	41.205
1314	*Turdus ignobilis*	Turdidae	Genetic-based	Absent	[44]	67	4.941
1315	*Turdus grayi*	Turdidae	Genetic-based	Absent	[90]	76	15.895
1316	*Turdus rufopalliatus*	Turdidae	Genetic-based	Absent	[37]	80	22.347
1317	*Turdus obsoletus*	Turdidae	Genetic-based	Absent	[133]	72	3.625
1318	*Turdus assimilis*	Turdidae	Genetic-based	Absent	[37]	75	18.124
1319	*Turdus albicollis*	Turdidae	Birth-death polytomy resolution	Absent	[56]	62	11.245
1320	*Tarsiger cyanurus*	Muscicapidae	Genetic-based	Absent	[121]	14	41.097
1321	*Cossypha heuglini*	Muscicapidae	Genetic-based	Absent	[46]	36.5	7.794
1322	*Cossypha anomala*	Muscicapidae	Genetic-based	Absent	[24]	24.5	15.673
1323	*Cossypha humeralis*	Muscicapidae	Genetic-based	Absent	[46]	24	23.379
1324	*Niltava sundara*	Muscicapidae	Genetic-based	Absent	[141]	21.5	24.432
1325	*Cyanoptila cyanomelana*	Muscicapidae	Genetic-based	Absent	[111]	25	20.140
1326	*Copsychus fulicatus*	Muscicapidae	Genetic-based	Complete	[35]	18.5	20.250
1327	*Cercotrichas galactotes*	Muscicapidae	Genetic-based	Complete	[17]	24	20.117
1328	*Cercotrichas podobe*	Muscicapidae	Genetic-based	Complete	[141]	25.5	19.380
1329	*Bradornis mariquensis*	Muscicapidae	Genetic-based	Absent	[46]	29	21.314
1330	*Melaenornis silens*	Muscicapidae	Genetic-based	Absent	[46]	29	27.720
1331	*Melaenornis pammelaina*	Muscicapidae	Birth-death polytomy resolution	Absent	[46]	27	15.944
1332	*Muscicapa striata*	Muscicapidae	Genetic-based	Complete	[121]	16.5	17.859
1333	*Muscicapa gambagae*	Muscicapidae	Genetic-based	Absent	[111]	13	10.597
1334	*Muscicapa sibirica*	Muscicapidae	Genetic-based	Complete	[121]	10.5	27.516
1335	*Muscicapa dauurica*	Muscicapidae	Genetic-based	Absent	[111]	12	26.604
1336	*Erithacus rubecula*	Muscicapidae	Genetic-based	Absent	[17]	19	47.609
1337	*Larvivora brunnea*	Muscicapidae	Genetic-based	Absent	[141]	17	21.602
1338	*Larvivora cyane*	Muscicapidae	Genetic-based	Absent	[77]	14.5	28.040
1339	*Larvivora akahige*	Muscicapidae	Genetic-based	Absent	[141]	20	35.991
1340	*Larvivora sibilans*	Muscicapidae	Genetic-based	Absent	[111]	16	41.367
1341	*Irania gutturalis*	Muscicapidae	Genetic-based	Absent	[121]	24	18.477
1342	*Luscinia svecica*	Muscicapidae	Genetic-based	Absent	[121]	18.5	38.658
1343	*Luscinia luscinia*	Muscicapidae	Genetic-based	Absent	[121]	26	19.576
1344	*Luscinia megarhynchos*	Muscicapidae	Genetic-based	Absent	[121]	24	23.628
1345	*Calliope pectoralis*	Muscicapidae	Genetic-based	Absent	[121]	23	32.914
1346	*Calliope calliope*	Muscicapidae	Genetic-based	Absent	[77]	22.5	39.548
1347	*Monticola rufocinereus*	Muscicapidae	Genetic-based	Absent	[111]	23.5	8.390
1348	*Ficedula zanthopygia*	Muscicapidae	Genetic-based	Absent	[77]	12	24.197
1349	*Ficedula narcissina*	Muscicapidae	Genetic-based	Absent	[77]	11.5	27.117
1350	*Ficedula superciliaris*	Muscicapidae	Genetic-based	Absent	[141]	8	23.980
1351	*Ficedula parva*	Muscicapidae	Genetic-based	Absent	[121]	10	40.669

1352	*Ficedula albicilla*	Muscicapidae	Genetic-based	Absent	77	11	37.586
1353	*Ficedula semitorquata*	Muscicapidae	Genetic-based	Absent	121	13.5	17.691
1354	*Ficedula hypoleuca*	Muscicapidae	Genetic-based	Absent	121	13.5	32.453
1355	*Ficedula albicollis*	Muscicapidae	Genetic-based	Absent	121	13.5	18.425
1356	*Phoenicurus erythronotus*	Muscicapidae	Genetic-based	Absent	111	18.5	36.319
1357	*Phoenicurus frontalis*	Muscicapidae	Genetic-based	Absent	141	15.5	28.676
1358	*Phoenicurus coeruleocephala*	Muscicapidae	Genetic-based	Absent	141	15	36.674
1359	*Phoenicurus schisticeps*	Muscicapidae	Genetic-based	Absent	141	16	30.867
1360	*Phoenicurus phoenicurus*	Muscicapidae	Genetic-based	Absent	121	17	34.189
1361	*Phoenicurus moussieri*	Muscicapidae	Genetic-based	Absent	111	14.5	32.722
1362	*Phoenicurus erythrogastrus*	Muscicapidae	Genetic-based	Absent	111	25	40.222
1363	*Phoenicurus ochruros*	Muscicapidae	Genetic-based	Absent	121	16	33.523
1364	*Phoenicurus auroreus*	Muscicapidae	Genetic-based	Absent	77	15.5	37.776
1365	*Monticola rufiventris*	Muscicapidae	Genetic-based	Absent	141	54.5	26.218
1366	*Monticola cinclorhyncha*	Muscicapidae	Genetic-based	Absent	141	35	23.065
1367	*Monticola brevipes*	Muscicapidae	Genetic-based	Absent	46	33	22.663
1368	*Monticola solitarius*	Muscicapidae	Genetic-based	Absent	121	53.5	19.667
1369	*Monticola saxatilis*	Muscicapidae	Genetic-based	Absent	121	52.5	22.020
1370	*Saxicola rubetra*	Muscicapidae	Genetic-based	Absent	121	16	27.487
1371	*Saxicola ferreus*	Muscicapidae	Genetic-based	Absent	141	15	23.239
1372	*Saxicola caprata*	Muscicapidae	Genetic-based	Absent	111	18	17.479
1373	*Saxicola torquatus*	Muscicapidae	Genetic-based	Absent	121	13.5	17.413
1374	*Saxicola dacotiae*	Muscicapidae	Genetic-based	Absent	141	14	28.384
1375	*Oenanthe lugentoides*	Muscicapidae	Birth-death polytomy resolution	Absent	9	23	18.906
1376	*Myrmecocichla aethiops*	Muscicapidae	Genetic-based	Complete	17	57	7.439
1377	*Oenanthe oenanthe*	Muscicapidae	Genetic-based	Absent	17	25.5	31.788
1378	*Oenanthe isabellina*	Muscicapidae	Genetic-based	Absent	17	30	24.082
1379	*Oenanthe bottae*	Muscicapidae	Genetic-based	Absent	111	36	17.331
1380	*Oenanthe monacha*	Muscicapidae	Genetic-based	Absent	17	20	25.145
1381	*Oenanthe deserti*	Muscicapidae	Genetic-based	Absent	17	18	26.001
1382	*Oenanthe cypriaca*	Muscicapidae	Genetic-based	Absent	17	18	22.227
1383	*Oenanthe hispanica*	Muscicapidae	Genetic-based	Absent	17	16.5	29.583
1384	*Oenanthe pleschanka*	Muscicapidae	Genetic-based	Absent	17	19	24.980
1385	*Oenanthe moesta*	Muscicapidae	Genetic-based	Absent	17	32	29.133
1386	*Oenanthe melanura*	Muscicapidae	Genetic-based	Absent	17	15.5	20.398
1387	*Oenanthe picata*	Muscicapidae	Genetic-based	Absent	111	22.5	31.727
1388	*Oenanthe albonigra*	Muscicapidae	Genetic-based	Absent	111	25	28.808
1389	*Oenanthe leucopyga*	Muscicapidae	Genetic-based	Absent	17	31	23.975
1390	*Oenanthe leucura*	Muscicapidae	Genetic-based	Absent	17	40	30.666
1391	*Oenanthe xanthoprymna*	Muscicapidae	Genetic-based	Absent	17	21	26.470
1392	*Oenanthe chrysopygia*	Muscicapidae	Genetic-based	Absent	17	23.5	26.609
1393	*Oenanthe finschii*	Muscicapidae	Genetic-based	Absent	17	27	35.362
1394	*Oenanthe lugens*	Muscicapidae	Genetic-based	Absent	17	22	19.321
1395	*Hedydipna collaris*	Nectariniidae	Genetic-based	Complete	15	8	9.673
1396	*Hedydipna metallica*	Nectariniidae	Birth-death polytomy resolution	Complete	111	7	20.122
1397	*Cinnyris osea*	Nectariniidae	Birth-death polytomy resolution	Complete	17	7	18.431
1398	*Cinnyris cupreus*	Nectariniidae	Birth-death polytomy resolution	Complete	15	8.5	1.514
1399	*Chalcomitra amethystina*	Nectariniidae	Birth-death polytomy resolution	Complete	15	13	15.040
1400	*Cinnyris mariquensis*	Nectariniidae	Genetic-based	Complete	125	11.5	5.903
1401	*Chalcomitra senegalensis*	Nectariniidae	Genetic-based	Complete	15	11.5	7.373
1402	*Cyanomitra olivacea*	Nectariniidae	Genetic-based	Complete	22	10	9.885
1403	*Cinnyris bifasciatus*	Nectariniidae	Birth-death polytomy resolution	Complete	15	7	11.071
1404	*Cinnyris habessinicus*	Nectariniidae	Birth-death polytomy resolution	Complete	111	9.5	11.762
1405	*Nectarinia tacazze*	Nectariniidae	Birth-death polytomy resolution	Complete	12	14.5	5.712
1406	*Cinnyris venustus*	Nectariniidae	Genetic-based	Complete	15	7.5	2.177
1407	*Cinnyris talatala*	Nectariniidae	Genetic-based	Complete	15	8	20.562
1408	*Peucedramus taeniatus*	Peucedramidae	Genetic-based	Absent	90	11	23.538
1409	*Prunella ocularis*	Prunellidae	Birth-death polytomy resolution	Absent	121	19	27.370
1410	*Prunella fulvescens*	Prunellidae	Genetic-based	Absent	17	20	40.065
1411	*Prunella atrogularis*	Prunellidae	Genetic-based	Absent	121	19	48.070
1412	*Prunella montanella*	Prunellidae	Genetic-based	Absent	121	17.5	53.108
1413	*Prunella collaris*	Prunellidae	Genetic-based	Absent	121	38	40.774
1414	*Prunella modularis*	Prunellidae	Genetic-based	Absent	54	19	50.442
1415	*Amblyospiza albifrons*	Ploceidae	Genetic-based	Absent	67	45	12.521
1416	*Philetairus socius*	Ploceidae	Genetic-based	Complete	15	28	24.586
1417	*Quelea erythrops*	Ploceidae	Birth-death polytomy resolution	Complete	15	20	7.703
1418	*Quelea quelea*	Ploceidae	Genetic-based	Complete	15	20.5	7.314
1419	*Euplectes afer*	Ploceidae	Genetic-based	Complete	125	15.5	8.343

1420	*Euplectes axillaris*	Ploceidae	Genetic-based	Complete	[15]	26	8.724
1421	*Euplectes progne*	Ploceidae	Genetic-based	Complete	[22]	36	17.556
1422	*Euplectes orix*	Ploceidae	Genetic-based	Complete	[15]	23.5	17.224
1423	*Euplectes ardens*	Ploceidae	Genetic-based	Complete	[15]	19	10.321
1424	*Ploceus bannermani*	Ploceidae	Birth-death polytomy resolution	Complete	[7]	31	6.961
1425	*Ploceus manyar*	Ploceidae	Genetic-based	Complete	[17]	19	11.856
1426	*Ploceus xanthops*	Ploceidae	Birth-death polytomy resolution	Complete	[125]	41	14.417
1427	*Ploceus baglafecht*	Ploceidae	Birth-death polytomy resolution	Complete	[12]	31	3.130
1428	*Ploceus xanthopterus*	Ploceidae	Birth-death polytomy resolution	Complete	[15]	23	19.316
1429	*Ploceus ocularis*	Ploceidae	Genetic-based	Complete	[125]	27	12.086
1430	*Ploceus capensis*	Ploceidae	Genetic-based	Complete	[15]	44	28.700
1431	*Ploceus velatus*	Ploceidae	Genetic-based	Absent	[46]	31	21.823
1432	*Sporaeginthus subflavus*	Estrildidae	Genetic-based	Complete	[15]	8	7.800
1433	*Estrilda erythronotos*	Estrildidae	Genetic-based	Partial	[45]	9	9.631
1434	*Estrilda astrild*	Estrildidae	Genetic-based	Complete	[15]	8	10.465
1435	*Pytilia melba*	Estrildidae	Genetic-based	Complete	[15]	15.5	6.415
1436	*Lagonosticta senegala*	Estrildidae	Genetic-based	Complete	[15]	9.5	6.887
1437	*Lagonosticta rhodopareia*	Estrildidae	Genetic-based	Complete	[45]	10	9.054
1438	*Granatina granatina*	Estrildidae	Genetic-based	Complete	[15]	11.5	7.940
1439	*Uraeginthus angolensis*	Estrildidae	Genetic-based	Complete	[45]	9.5	15.594
1440	*Heteromunia pectoralis*	Estrildidae	Genetic-based	Complete	[30]	15.5	38.822
1441	*Lonchura oryzivora*	Estrildidae	Genetic-based	Complete	[71]	25	7.705
1442	*Spermestes cucullata*	Estrildidae	Genetic-based	Complete	[15]	10	9.181
1443	*Lonchura flaviprymna*	Estrildidae	Birth-death polytomy resolution	Complete	[71]	11.5	14.813
1444	*Lonchura punctulata*	Estrildidae	Genetic-based	Complete	[71]	14	1.531
1445	*Lonchura pallida*	Estrildidae	Genetic-based	Complete	[71]	12	5.506
1446	*Stagonopleura oculata*	Estrildidae	Birth-death polytomy resolution	Complete	[71]	13	33.287
1447	*Stagonopleura bella*	Estrildidae	Birth-death polytomy resolution	Complete	[71]	13.5	38.333
1448	*Emblema pictum*	Estrildidae	Genetic-based	Complete	[71]	10.5	23.424
1449	*Taeniopygia bichenovii*	Estrildidae	Genetic-based	Complete	[30]	9.5	24.459
1450	*Taeniopygia guttata*	Estrildidae	Genetic-based	Complete	[138]	12.5	9.250
1451	*Poephila personata*	Estrildidae	Genetic-based	Complete	[71]	14	16.391
1452	*Poephila cincta*	Estrildidae	Genetic-based	Complete	[71]	15	18.006
1453	*Poephila acuticauda*	Estrildidae	Genetic-based	Complete	[30]	14	16.042
1454	*Neochmia ruficauda*	Estrildidae	Genetic-based	Complete	[71]	11	18.816
1455	*Neochmia modesta*	Estrildidae	Genetic-based	Complete	[71]	12.5	24.439
1456	*Neochmia phaeton*	Estrildidae	Genetic-based	Complete	[71]	10	17.044
1457	*Neochmia temporalis*	Estrildidae	Genetic-based	Complete	[71]	11.5	24.760
1458	*Motacilla grandis*	Motacillidae	Birth-death polytomy resolution	Absent	[2]	28	35.799
1459	*Motacilla aguimp*	Motacillidae	Genetic-based	Absent	[2]	26	5.191
1460	*Motacilla maderaspatensis*	Motacillidae	Genetic-based	Absent	[2]	33	20.674
1461	*Motacilla cinerea*	Motacillidae	Genetic-based	Absent	[2]	17.5	27.550
1462	*Motacilla alba*	Motacillidae	Genetic-based	Absent	[2]	21	35.710
1463	*Motacilla flava*	Motacillidae	Genetic-based	Absent	[2]	18	18.305
1464	*Motacilla citreola*	Motacillidae	Genetic-based	Absent	[2]	21.5	41.200
1465	*Dendronanthus indicus*	Motacillidae	Birth-death polytomy resolution	Absent	[2]	15.5	21.870
1466	*Anthus godlewskii*	Motacillidae	Genetic-based	Absent	[2]	24	31.067
1467	*Anthus novaeseelandiae*	Motacillidae	Genetic-based	Absent	[71]	18	28.989
1468	*Anthus richardi*	Motacillidae	Genetic-based	Absent	[2]	30	33.724
1469	*Anthus berthelotii*	Motacillidae	Genetic-based	Absent	[2]	17.5	30.378
1470	*Anthus similis*	Motacillidae	Genetic-based	Absent	[111]	29	0.902
1471	*Anthus rufulus*	Motacillidae	Birth-death polytomy resolution	Partial	[2]	20	12.665
1472	*Anthus spragueii*	Motacillidae	Genetic-based	Absent	[90]	25	36.891
1473	*Anthus roseatus*	Motacillidae	Birth-death polytomy resolution	Absent	[2]	21	33.216
1474	*Anthus gustavi*	Motacillidae	Genetic-based	Absent	[2]	19	31.116
1475	*Anthus sylvanus*	Motacillidae	Birth-death polytomy resolution	Absent	[2]	18.5	28.669
1476	*Anthus hodgsoni*	Motacillidae	Genetic-based	Absent	[2]	21.5	35.343
1477	*Anthus trivialis*	Motacillidae	Genetic-based	Absent	[2]	24	22.847
1478	*Anthus rubescens*	Motacillidae	Genetic-based	Absent	[2]	21	44.817
1479	*Anthus nilghiriensis*	Motacillidae	Genetic-based	Absent	[2]	22	10.355
1480	*Anthus cervinus*	Motacillidae	Genetic-based	Absent	[2]	22.5	33.998
1481	*Anthus pratensis*	Motacillidae	Birth-death polytomy resolution	Absent	[2]	19	42.928
1482	*Anthus petrosus*	Motacillidae	Genetic-based	Absent	[2]	25	51.947
1483	*Anthus spinoletta*	Motacillidae	Genetic-based	Absent	[2]	21	39.412
1484	*Carpospiza brachydactyla*	Passeridae	Genetic-based	Partial	[141]	23	25.135
1485	*Gymnoris dentata*	Passeridae	Birth-death polytomy resolution	Complete	[111]	19	10.860
1486	*Gymnoris xanthocollis*	Passeridae	Birth-death polytomy resolution	Complete	[35]	17	23.334
1487	*Montifringilla nivalis*	Passeridae	Genetic-based	Complete	[121]	44	40.030

1488	*Petronia petronia*	Passeridae	Genetic-based	Complete	17	32.5	40.138
1489	*Passer cinnamomeus*	Passeridae	Genetic-based	Complete	141	18	32.677
1490	*Passer montanus*	Passeridae	Genetic-based	Complete	141	23.5	30.060
1491	*Passer shelleyi*	Passeridae	Birth-death polytomy resolution	Complete	141	17	5.498
1492	*Passer flaveolus*	Passeridae	Genetic-based	Complete	141	20	13.140
1493	*Passer luteus*	Passeridae	Genetic-based	Complete	17	13.5	16.916
1494	*Passer simplex*	Passeridae	Birth-death polytomy resolution	Complete	111	19.5	24.190
1495	*Passer iagoensis*	Passeridae	Birth-death polytomy resolution	Complete	17	18	15.966
1496	*Passer hispaniolensis*	Passeridae	Genetic-based	Complete	17	26.5	35.451
1497	*Passer domesticus*	Passeridae	Genetic-based	Complete	17	29.5	38.549
1498	*Passer insularis*	Passeridae	Genetic-based	Complete	141	27.5	12.411
1499	*Passer ammodendri*	Passeridae	Genetic-based	Complete	111	28.5	41.848
1500	*Passer euchlorus*	Passeridae	Birth-death polytomy resolution	Complete	111	14.5	15.872
1501	*Passer motitensis*	Passeridae	Birth-death polytomy resolution	Complete	141	31	21.522
1502	*Passer moabiticus*	Passeridae	Birth-death polytomy resolution	Complete	43	13.5	33.154
1503	*Passer rufocinctus*	Passeridae	Birth-death polytomy resolution	Complete	141	28.5	0.743
1504	*Passer melanurus*	Passeridae	Genetic-based	Complete	15	27.5	23.709
1505	*Passer eminibey*	Passeridae	Birth-death polytomy resolution	Complete	141	14.5	2.700
1506	*Passer griseus*	Passeridae	Genetic-based	Complete	15	31	0.089
1507	*Passer pyrrhonotus*	Passeridae	Birth-death polytomy resolution	Complete	111	17	29.224
1508	*Fringilla montifringilla*	Fringillidae	Genetic-based	Absent	121	23	42.364
1509	*Fringilla teydea*	Fringillidae	Genetic-based	Absent	17	30.5	28.278
1510	*Fringilla coelebs*	Fringillidae	Genetic-based	Absent	121	22	48.629
1511	*Euphonia xanthogaster*	Fringillidae	Birth-death polytomy resolution	Absent	44	12.5	6.039
1512	*Euphonia anneae*	Fringillidae	Birth-death polytomy resolution	Absent	133	15	9.140
1513	*Mycerobas carnipes*	Fringillidae	Genetic-based	Absent	111	58	36.336
1514	*Coccothraustes vespertinus*	Fringillidae	Genetic-based	Absent	90	61.5	38.636
1515	*Coccothraustes coccothraustes*	Fringillidae	Genetic-based	Absent	54	59	43.551
1516	*Loxops coccineus*	Fringillidae	Genetic-based	Complete	31	11	19.627
1517	*Chlorodrepanis virens*	Fringillidae	Genetic-based	Complete	31	13.5	20.093
1518	*Himatione sanguinea*	Fringillidae	Genetic-based	Complete	31	15	20.574
1519	*Drepanis coccinea*	Fringillidae	Genetic-based	Complete	31	18	20.654
1520	*Bucanetes mongolicus*	Fringillidae	Genetic-based	Absent	111	22	40.282
1521	*Bucanetes githagineus*	Fringillidae	Genetic-based	Partial	121	20.5	27.221
1522	*Pinicola enucleator*	Fringillidae	Genetic-based	Absent	121	60	52.513
1523	*Pyrrhula pyrrhula*	Fringillidae	Genetic-based	Absent	54	29.5	50.628
1524	*Pyrrhula murina*	Fringillidae	Birth-death polytomy resolution	Absent	111	30	37.800
1525	*Carpodacus sibiricus*	Fringillidae	Genetic-based	Absent	111	21	42.453
1526	*Carpodacus rubicilla*	Fringillidae	Genetic-based	Absent	111	45	37.896
1527	*Carpodacus synoicus*	Fringillidae	Birth-death polytomy resolution	Absent	111	20.5	29.532
1528	*Carpodacus roseus*	Fringillidae	Genetic-based	Absent	111	28	47.871
1529	*Carpodacus erythrinus*	Fringillidae	Genetic-based	Complete	121	26	40.313
1530	*Haemorhous mexicanus*	Fringillidae	Genetic-based	Partial	90	21	36.041
1531	*Haemorhous cassinii*	Fringillidae	Genetic-based	Absent	90	29	36.354
1532	*Haemorhous purpureus*	Fringillidae	Genetic-based	Absent	90	23	45.211
1533	*Leucosticte tephrocotis*	Fringillidae	Genetic-based	Absent	90	38	52.517
1534	*Leucosticte atrata*	Fringillidae	Genetic-based	Absent	90	26.5	40.983
1535	*Leucosticte australis*	Fringillidae	Genetic-based	Absent	90	28	37.849
1536	*Chloris sinica*	Fringillidae	Genetic-based	Complete	75	20	35.356
1537	*Chloris chloris*	Fringillidae	Genetic-based	Partial	54	25.5	45.730
1538	*Rhodopechys sanguineus*	Fringillidae	Birth-death polytomy resolution	Partial	141	40	36.464
1539	*Rhodospiza obsoleta*	Fringillidae	Genetic-based	Partial	141	22.5	35.902
1540	*Crithagra rothschildi*	Fringillidae	Birth-death polytomy resolution	Absent	111	14	19.711
1541	*Crithagra mozambica*	Fringillidae	Genetic-based	Absent	46	12.5	8.714
1542	*Serinus canaria*	Fringillidae	Genetic-based	Absent	111	17.5	33.388
1543	*Serinus serinus*	Fringillidae	Genetic-based	Absent	121	11.5	43.884
1544	*Serinus syriacus*	Fringillidae	Birth-death polytomy resolution	Absent	17	13	31.387
1545	*Serinus pusillus*	Fringillidae	Genetic-based	Absent	17	11.5	37.379
1546	*Carduelis carduelis*	Fringillidae	Genetic-based	Partial	54	21.5	43.152
1547	*Carduelis citrinella*	Fringillidae	Genetic-based	Absent	54	13	42.759
1548	*Acanthis flammea*	Fringillidae	Genetic-based	Absent	54	13.5	57.402
1549	*Acanthis hornemanni*	Fringillidae	Genetic-based	Absent	111	15	59.072
1550	*Loxia leucoptera*	Fringillidae	Genetic-based	Absent	121	32.5	52.091
1551	*Loxia curvirostra*	Fringillidae	Genetic-based	Absent	121	38	40.561
1552	*Loxia scotica*	Fringillidae	Genetic-based	Absent	121	42.5	57.275
1553	*Loxia pytyopsittacus*	Fringillidae	Genetic-based	Absent	121	56.5	61.759
1554	*Linaria flavirostris*	Fringillidae	Genetic-based	Absent	111	15	49.550
1555	*Linaria cannabina*	Fringillidae	Genetic-based	Partial	54	20.5	41.491

1556	*Spinus spinus*	Fringillidae	Genetic-based	Absent	54	14.5	45.430
1557	*Spinus pinus*	Fringillidae	Genetic-based	Absent	90	14.5	40.313
1558	*Carduelis corsicana*	Fringillidae	Birth-death polytomy resolution	Absent	111	11.5	40.954
1559	*Spinus barbatus*	Fringillidae	Genetic-based	Partial	97	16.5	40.109
1560	*Spinus tristis*	Fringillidae	Genetic-based	Absent	90	15	37.979
1561	*Spinus psaltria*	Fringillidae	Genetic-based	Partial	90	8.5	18.851
1562	*Spinus lawrencei*	Fringillidae	Genetic-based	Absent	90	11.5	34.963
1563	*Plectrophenax nivalis*	Calcariidae	Genetic-based	Absent	89	37	60.097
1564	*Plectrophenax hyperboreus*	Calcariidae	Genetic-based	Absent	89	50	61.849
1565	*Rhynchophanes mccownii*	Calcariidae	Genetic-based	Absent	90	25.5	38.413
1566	*Calcarius lapponicus*	Calcariidae	Genetic-based	Absent	121	29	56.722
1567	*Calcarius ornatus*	Calcariidae	Genetic-based	Absent	90	20	36.796
1568	*Calcarius pictus*	Calcariidae	Genetic-based	Absent	90	26.5	50.486
1569	*Pheucticus chrysopeplus*	Cardinalidae	Birth-death polytomy resolution	Absent	40	76	22.154
1570	*Pheucticus chrysogaster*	Cardinalidae	Birth-death polytomy resolution	Absent	40	56	1.973
1571	*Pheucticus tibialis*	Cardinalidae	Birth-death polytomy resolution	Absent	40	62	9.533
1572	*Pheucticus aureoventris*	Cardinalidae	Genetic-based	Absent	40	49	12.025
1573	*Pheucticus ludovicianus*	Cardinalidae	Genetic-based	Absent	90	47	26.658
1574	*Pheucticus melanocephalus*	Cardinalidae	Genetic-based	Absent	90	42	33.900
1575	*Spiza americana*	Cardinalidae	Genetic-based	Absent	90	30	25.247
1576	*Cyanoloxia cyanoides*	Cardinalidae	Genetic-based	Absent	56	30	7.251
1577	*Passerina amoena*	Cardinalidae	Genetic-based	Partial	90	15.5	34.645
1578	*Passerina caerulea*	Cardinalidae	Genetic-based	Partial	90	29.5	26.948
1579	*Passerina cyanea*	Cardinalidae	Genetic-based	Partial	90	15	29.872
1580	*Passerina versicolor*	Cardinalidae	Genetic-based	Partial	90	13	23.436
1581	*Passerina ciris*	Cardinalidae	Genetic-based	Partial	90	16	23.148
1582	*Habia rubica*	Cardinalidae	Genetic-based	Absent	38	33	4.658
1583	*Habia fuscicauda*	Cardinalidae	Genetic-based	Absent	38	40	15.811
1584	*Piranga rubra*	Cardinalidae	Genetic-based	Absent	90	31	12.083
1585	*Piranga flava*	Cardinalidae	Genetic-based	Absent	90	35	16.099
1586	*Piranga bidentata*	Cardinalidae	Genetic-based	Absent	89	38	20.576
1587	*Piranga olivacea*	Cardinalidae	Genetic-based	Absent	89	31.5	17.608
1588	*Piranga ludoviciana*	Cardinalidae	Genetic-based	Absent	89	30	36.146
1589	*Cardinalis sinuatus*	Cardinalidae	Genetic-based	Absent	90	35.5	27.592
1590	*Cardinalis phoeniceus*	Cardinalidae	Genetic-based	Complete	40	30	11.488
1591	*Cardinalis cardinalis*	Cardinalidae	Genetic-based	Absent	90	44	31.492
1592	*Rhodothraupis celaeno*	Cardinalidae	Genetic-based	Absent	40	60	22.848
1593	*Periporphyrus erythromelas*	Cardinalidae	Genetic-based	Absent	40	48	1.251
1594	*Caryothraustes poliogaster*	Cardinalidae	Genetic-based	Absent	40	42	13.712
1595	*Caryothraustes canadensis*	Cardinalidae	Genetic-based	Complete	56	33.5	7.209
1596	*Diuca diuca*	Thraupidae	Genetic-based	Absent	97	37	36.589
1597	*Coereba flaveola*	Thraupidae	Genetic-based	Complete	56	10	2.887
1598	*Tiaris olivaceus*	Thraupidae	Genetic-based	Absent	23	8	12.544
1599	*Phonipara canora*	Thraupidae	Genetic-based	Absent	23	8	21.404
1600	*Tangara chilensis*	Thraupidae	Genetic-based	Partial	56	19	5.022
1601	*Tangara gyrola*	Thraupidae	Genetic-based	Partial	56	22	2.063
1602	*Tangara arthus*	Thraupidae	Genetic-based	Absent	23	21	9.790
1603	*Stilpnia vitriolina*	Thraupidae	Genetic-based	Absent	23	22.5	4.125
1604	*Ixothraupis punctata*	Thraupidae	Genetic-based	Absent	23	15	5.222
1605	*Ixothraupis varia*	Thraupidae	Genetic-based	Absent	56	10	3.730
1606	*Pipraeidea bonariensis*	Thraupidae	Genetic-based	Absent	23	36	28.399
1607	*Stilpnia cyanoptera*	Thraupidae	Genetic-based	Absent	23	43.5	9.213
1608	*Thraupis sayaca*	Thraupidae	Genetic-based	Absent	23	31	18.793
1609	*Thraupis episcopus*	Thraupidae	Genetic-based	Complete	56	36	2.220
1610	*Thraupis abbas*	Thraupidae	Genetic-based	Absent	23	46.5	17.725
1611	*Thraupis palmarum*	Thraupidae	Genetic-based	Absent	23	37.5	9.639
1612	*Thraupis ornata*	Thraupidae	Genetic-based	Absent	23	35	20.065
1613	*Tangara cyanocephala*	Thraupidae	Genetic-based	Absent	23	19	17.273
1614	*Anisognathus melanogenys*	Thraupidae	Genetic-based	Complete	23	40	10.778
1615	*Anisognathus lacrymosus*	Thraupidae	Genetic-based	Complete	23	32	0.791
1616	*Anisognathus igniventris*	Thraupidae	Genetic-based	Complete	23	31	14.557
1617	*Cnemathraupis eximia*	Thraupidae	Genetic-based	Complete	23	60	1.107
1618	*Buthraupis montana*	Thraupidae	Genetic-based	Complete	23	87	3.495
1619	*Anisognathus notabilis*	Thraupidae	Genetic-based	Complete	23	50	0.507
1620	*Thraupis glaucocolpa*	Thraupidae	Birth-death polytomy resolution	Absent	23	34	9.790
1621	*Anisognathus somptuosus*	Thraupidae	Genetic-based	Complete	23	45	1.270
1622	*Saltator maximus*	Thraupidae	Genetic-based	Absent	23	47.5	1.627
1623	*Saltator grossus*	Thraupidae	Genetic-based	Absent	23	47	0.769

1624	*Chlorophanes spiza*	Thraupidae	Genetic-based	Complete	[56]	18.5	5.446
1625	*Hemithraupis flavicollis*	Thraupidae	Genetic-based	Complete	[23]	13	7.273
1626	*Volatinia jacarina*	Thraupidae	Genetic-based	Complete	[23]	10	3.190
1627	*Tachyphonus luctuosus*	Thraupidae	Genetic-based	Absent	[23]	13	1.303
1628	*Tachyphonus cristatus*	Thraupidae	Genetic-based	Complete	[56]	20	9.310
1629	*Tachyphonus surinamus*	Thraupidae	Genetic-based	Complete	[56]	21	0.407
1630	*Lanio fulvus*	Thraupidae	Genetic-based	Complete	[56]	24.5	0.795
1631	*Tachyphonus rufus*	Thraupidae	Genetic-based	Absent	[23]	34	11.815
1632	*Ramphocelus passerinii*	Thraupidae	Genetic-based	Partial	[134]	32	13.340
1633	*Ramphocelus carbo*	Thraupidae	Genetic-based	Complete	[56]	27	6.624
1634	*Ramphocelus bresilius*	Thraupidae	Genetic-based	Complete	[23]	32	16.656
1635	*Thlypopsis fulviceps*	Thraupidae	Birth-death polytomy resolution	Absent	[23]	12	8.932
1636	*Rhopospina fruticeti*	Thraupidae	Genetic-based	Partial	[97]	38.5	29.834
1637	*Tersina viridis*	Thraupidae	Genetic-based	Absent	[23]	31	8.883
1638	*Cyanerpes cyaneus*	Thraupidae	Genetic-based	Complete	[23]	15	0.789
1639	*Cyanerpes nitidus*	Thraupidae	Genetic-based	Complete	[23]	9	1.523
1640	*Cyanerpes caeruleus*	Thraupidae	Genetic-based	Complete	[23]	11	3.123
1641	*Cyanerpes lucidus*	Thraupidae	Genetic-based	Complete	[23]	11	12.157
1642	*Phrygilus gayi*	Thraupidae	Genetic-based	Absent	[97]	26	38.003
1643	*Diglossa baritula*	Thraupidae	Genetic-based	Complete	[108]	10.5	16.759
1644	*Diglossa albilatera*	Thraupidae	Genetic-based	Absent	[23]	10	0.178
1645	*Sporophila angolensis*	Thraupidae	Genetic-based	Complete	[23]	13	9.433
1646	*Sporophila funerea*	Thraupidae	Genetic-based	Absent	[23]	13.5	7.664
1647	*Sporophila torqueola*	Thraupidae	Genetic-based	Absent	[90]	9.5	20.289
1648	*Sporophila castaneiventris*	Thraupidae	Genetic-based	Complete	[56]	8	4.472
1649	*Sporophila corvina*	Thraupidae	Genetic-based	Complete	[23]	11	13.395
1650	*Sporophila intermedia*	Thraupidae	Genetic-based	Complete	[23]	13.5	6.881
1651	*Teretistris fornsi*	Teretistridae	Birth-death polytomy resolution	Partial	[92]	10	21.457
1652	*Icteria virens*	Icteriidae	Genetic-based	Partial	[89]	27	30.567
1653	*Seiurus aurocapilla*	Parulidae	Genetic-based	Absent	[90]	20.5	34.142
1654	*Helmitheros vermivorum*	Parulidae	Genetic-based	Absent	[90]	14.5	25.182
1655	*Mniotilta varia*	Parulidae	Genetic-based	Absent	[90]	12	28.686
1656	*Parkesia noveboracensis*	Parulidae	Genetic-based	Absent	[90]	19	33.403
1657	*Parkesia motacilla*	Parulidae	Genetic-based	Absent	[90]	21.5	25.008
1658	*Geothlypis trichas*	Parulidae	Genetic-based	Absent	[90]	11.5	36.520
1659	*Geothlypis poliocephala*	Parulidae	Genetic-based	Absent	[90]	15	16.394
1660	*Geothlypis formosa*	Parulidae	Genetic-based	Absent	[90]	16	25.774
1661	*Oporornis agilis*	Parulidae	Genetic-based	Absent	[90]	19	19.708
1662	*Geothlypis philadelphia*	Parulidae	Genetic-based	Absent	[90]	14	28.980
1663	*Geothlypis tolmiei*	Parulidae	Genetic-based	Absent	[90]	10.5	34.271
1664	*Protonotaria citrea*	Parulidae	Genetic-based	Absent	[90]	17	22.623
1665	*Limnothlypis swainsonii*	Parulidae	Genetic-based	Absent	[90]	17.5	27.588
1666	*Vermivora bachmanii*	Parulidae	Genetic-based	Absent	[90]	9	29.659
1667	*Vermivora cyanoptera*	Parulidae	Genetic-based	Absent	[90]	9	26.525
1668	*Vermivora chrysoptera*	Parulidae	Genetic-based	Absent	[90]	9.5	24.343
1669	*Leiothlypis peregrina*	Parulidae	Genetic-based	Absent	[90]	12.5	33.485
1670	*Oreothlypis superciliosa*	Parulidae	Genetic-based	Absent	[37]	9	20.482
1671	*Leiothlypis celata*	Parulidae	Genetic-based	Absent	[90]	9.5	41.627
1672	*Leiothlypis crissalis*	Parulidae	Genetic-based	Absent	[90]	10	23.590
1673	*Leiothlypis ruficapilla*	Parulidae	Genetic-based	Absent	[90]	10.5	35.226
1674	*Leiothlypis virginiae*	Parulidae	Genetic-based	Absent	[90]	8.5	30.088
1675	*Leiothlypis luciae*	Parulidae	Genetic-based	Absent	[90]	6.5	27.579
1676	*Myiothlypis rivularis*	Parulidae	Genetic-based	Absent	[56]	11	20.750
1677	*Basileuterus lachrymosus*	Parulidae	Genetic-based	Absent	[38]	11	21.107
1678	*Basileuterus rufifrons*	Parulidae	Genetic-based	Absent	[90]	11.5	16.388
1679	*Basileuterus culicivorus*	Parulidae	Genetic-based	Absent	[38]	8	16.226
1680	*Myioborus miniatus*	Parulidae	Genetic-based	Absent	[133]	10.5	4.463
1681	*Myioborus pictus*	Parulidae	Genetic-based	Absent	[90]	9	24.542
1682	*Cardellina canadensis*	Parulidae	Genetic-based	Absent	[90]	11	23.667
1683	*Cardellina rubrifrons*	Parulidae	Genetic-based	Absent	[90]	10	24.831
1684	*Cardellina pusilla*	Parulidae	Genetic-based	Absent	[90]	8	39.002
1685	*Setophaga citrina*	Parulidae	Genetic-based	Absent	[90]	11	25.353
1686	*Setophaga magnolia*	Parulidae	Genetic-based	Absent	[90]	9.5	35.619
1687	*Setophaga tigrina*	Parulidae	Genetic-based	Absent	[90]	13	35.184
1688	*Setophaga pitiayumi*	Parulidae	Genetic-based	Absent	[90]	6.5	3.912
1689	*Setophaga americana*	Parulidae	Genetic-based	Absent	[90]	8.5	32.441
1690	*Setophaga cerulea*	Parulidae	Genetic-based	Absent	[90]	9.5	15.704
1691	*Setophaga kirtlandii*	Parulidae	Genetic-based	Absent	[90]	14	33.933

1692	*Setophaga fusca*	Parulidae	Genetic-based	Absent	90	9.5	19.289
1693	*Setophaga petechia*	Parulidae	Genetic-based	Absent	90	12	33.349
1694	*Setophaga pensylvanica*	Parulidae	Genetic-based	Absent	90	10.5	28.773
1695	*Setophaga striata*	Parulidae	Genetic-based	Absent	90	15	30.178
1696	*Setophaga castanea*	Parulidae	Genetic-based	Absent	90	12.5	32.786
1697	*Setophaga caerulescens*	Parulidae	Genetic-based	Absent	90	10.5	29.160
1698	*Setophaga ruticilla*	Parulidae	Genetic-based	Absent	90	9.5	28.642
1699	*Setophaga palmarum*	Parulidae	Genetic-based	Absent	90	10	36.733
1700	*Setophaga coronata*	Parulidae	Genetic-based	Absent	90	13.5	38.211
1701	*Setophaga pinus*	Parulidae	Genetic-based	Absent	90	12	34.864
1702	*Setophaga dominica*	Parulidae	Genetic-based	Absent	90	10	24.967
1703	*Setophaga discolor*	Parulidae	Genetic-based	Absent	90	8.5	29.500
1704	*Setophaga nigrescens*	Parulidae	Genetic-based	Absent	90	8	34.410
1705	*Setophaga graciae*	Parulidae	Genetic-based	Absent	90	8.5	25.328
1706	*Setophaga occidentalis*	Parulidae	Genetic-based	Absent	90	10.5	28.708
1707	*Setophaga townsendi*	Parulidae	Genetic-based	Absent	90	9	37.595
1708	*Setophaga virens*	Parulidae	Genetic-based	Absent	90	9.5	31.570
1709	*Setophaga chrysoparia*	Parulidae	Genetic-based	Absent	90	11	23.024
1710	*Xanthocephalus xanthocephalus*	Icteridae	Genetic-based	Absent	90	78	34.105
1711	*Dolichonyx oryzivorus*	Icteridae	Genetic-based	Complete	89	42	9.863
1712	*Leistes loyca*	Icteridae	Genetic-based	Complete	97	105	38.675
1713	*Sturnella neglecta*	Icteridae	Genetic-based	Complete	89	98	38.104
1714	*Sturnella magna*	Icteridae	Genetic-based	Complete	89	85	23.899
1715	*Icterus cucullatus*	Icteridae	Genetic-based	Absent	37	24	28.486
1716	*Icterus dominicensis*	Icteridae	Genetic-based	Absent	92	37	18.745
1717	*Icterus spurius*	Icteridae	Genetic-based	Partial	90	21.5	27.096
1718	*Icterus graduacauda*	Icteridae	Genetic-based	Absent	90	46.5	22.978
1719	*Icterus parisorum*	Icteridae	Genetic-based	Absent	90	33	28.629
1720	*Icterus gularis*	Icteridae	Genetic-based	Absent	90	78	19.607
1721	*Icterus galbula*	Icteridae	Genetic-based	Absent	90	33.5	28.896
1722	*Icterus bullockii*	Icteridae	Genetic-based	Absent	90	38	32.823
1723	*Icterus pustulatus*	Icteridae	Genetic-based	Absent	90	39	20.252
1724	*Curaeus curaeus*	Icteridae	Genetic-based	Partial	97	83	40.617
1725	*Euphagus cyanocephalus*	Icteridae	Genetic-based	Complete	89	63	38.638
1726	*Euphagus carolinus*	Icteridae	Genetic-based	Complete	89	63	48.078
1727	*Quiscalus quiscula*	Icteridae	Genetic-based	Complete	89	106	42.949
1728	*Quiscalus major*	Icteridae	Genetic-based	Complete	89	160	33.068
1729	*Quiscalus mexicanus*	Icteridae	Genetic-based	Complete	89	178	20.100
1730	*Agelaius tricolor*	Icteridae	Genetic-based	Complete	89	60	38.678
1731	*Agelaius phoeniceus*	Icteridae	Genetic-based	Complete	89	52.5	38.536
1732	*Molothrus rufoaxillaris*	Icteridae	Genetic-based	Complete	126	56	27.653
1733	*Molothrus aeneus*	Icteridae	Genetic-based	Complete	89	63	21.529
1734	*Molothrus ater*	Icteridae	Genetic-based	Complete	89	42	41.654
1735	*Molothrus bonariensis*	Icteridae	Genetic-based	Partial	89	48	8.018
1736	*Emberiza impetuani*	Emberizidae	Genetic-based	Complete	124	16	21.869
1737	*Emberiza tahapisi*	Emberizidae	Genetic-based	Partial	111	15	6.204
1738	*Emberiza striolata*	Emberizidae	Genetic-based	Absent	141	14	18.709
1739	*Emberiza cineracea*	Emberizidae	Birth-death polytomy resolution	Absent	121	25	26.036
1740	*Emberiza bruniceps*	Emberizidae	Genetic-based	Complete	121	26	33.181
1741	*Emberiza melanocephala*	Emberizidae	Genetic-based	Complete	121	28	32.221
1742	*Emberiza calandra*	Emberizidae	Genetic-based	Complete	121	50	37.295
1743	*Emberiza cioides*	Emberizidae	Genetic-based	Complete	61	21.5	43.212
1744	*Emberiza cia*	Emberizidae	Genetic-based	Absent	54	23	39.768
1745	*Emberiza buchanani*	Emberizidae	Genetic-based	Absent	121	21.5	32.329
1746	*Emberiza cirlus*	Emberizidae	Genetic-based	Absent	121	24.5	39.780
1747	*Emberiza citrinella*	Emberizidae	Genetic-based	Absent	54	28.5	54.518
1748	*Emberiza leucocephalos*	Emberizidae	Genetic-based	Absent	121	28	48.869
1749	*Emberiza yessoensis*	Emberizidae	Genetic-based	Complete	136	13.5	36.967
1750	*Emberiza pallasi*	Emberizidae	Genetic-based	Absent	111	15	50.940
1751	*Emberiza schoeniclus*	Emberizidae	Genetic-based	Absent	54	19	46.572
1752	*Emberiza rustica*	Emberizidae	Genetic-based	Absent	121	20	46.819
1753	*Emberiza chrysophrys*	Emberizidae	Genetic-based	Absent	111	20	45.211
1754	*Emberiza aureola*	Emberizidae	Genetic-based	Partial	81	23.5	37.159
1755	*Emberiza spodocephala*	Emberizidae	Genetic-based	Absent	77	20	41.402
1756	*Emberiza rutila*	Emberizidae	Genetic-based	Absent	111	18	39.260
1757	*Emberiza fucata*	Emberizidae	Genetic-based	Complete	136	17.5	34.879
1758	*Emberiza pusilla*	Emberizidae	Genetic-based	Absent	77	15.5	46.264
1759	*Chondestes grammacus*	Passerellidae	Genetic-based	Partial	90	29	33.872

1760	*Calamospiza melanocorys*	Passerellidae	Genetic-based	Partial	90	40.5	36.320
1761	*Chlorospingus flavopectus*	Passerellidae	Genetic-based	Absent	38	19	2.640
1762	*Arremonops conirostris*	Passerellidae	Genetic-based	Complete	134	34	5.719
1763	*Arremonops rufivirgatus*	Passerellidae	Genetic-based	Absent	90	22.5	19.813
1764	*Ammodramus aurifrons*	Passerellidae	Genetic-based	Partial	56	17	3.869
1765	*Ammodramus savannarum*	Passerellidae	Genetic-based	Complete	89	21	27.446
1766	*Peucaea carpalis*	Passerellidae	Genetic-based	Absent	89	15	28.217
1767	*Peucaea humeralis*	Passerellidae	Genetic-based	Complete	39	23	18.281
1768	*Peucaea ruficauda*	Passerellidae	Genetic-based	Complete	37	33	16.355
1769	*Peucaea botterii*	Passerellidae	Genetic-based	Complete	89	20.5	21.455
1770	*Peucaea cassinii*	Passerellidae	Genetic-based	Complete	89	19	31.845
1771	*Peucaea aestivalis*	Passerellidae	Genetic-based	Complete	89	20.5	32.128
1772	*Amphispiza bilineata*	Passerellidae	Genetic-based	Partial	90	13.5	33.621
1773	*Amphispiza quinquestriata*	Passerellidae	Genetic-based	Absent	90	19.5	26.610
1774	*Spizella atrogularis*	Passerellidae	Genetic-based	Absent	90	12	29.520
1775	*Spizella passerina*	Passerellidae	Genetic-based	Absent	90	13	39.588
1776	*Spizella pallida*	Passerellidae	Genetic-based	Absent	90	12	39.456
1777	*Spizella pusilla*	Passerellidae	Genetic-based	Partial	90	13.5	37.031
1778	*Spizella breweri*	Passerellidae	Genetic-based	Absent	90	11.5	41.965
1779	*Arremon taciturnus*	Passerellidae	Birth-death polytomy resolution	Absent	56	25	5.720
1780	*Arremon brunneinucha*	Passerellidae	Genetic-based	Absent	133	39.5	3.580
1781	*Passerella iliaca*	Passerellidae	Genetic-based	Absent	90	40	48.124
1782	*Spizelloides arborea*	Passerellidae	Genetic-based	Absent	90	20	51.414
1783	*Junco phaeonotus*	Passerellidae	Genetic-based	Absent	90	20	23.538
1784	*Junco hyemalis*	Passerellidae	Genetic-based	Absent	90	20	43.981
1785	*Zonotrichia capensis*	Passerellidae	Genetic-based	Absent	19	24	18.264
1786	*Zonotrichia querula*	Passerellidae	Genetic-based	Absent	90	38.5	48.499
1787	*Zonotrichia albicollis*	Passerellidae	Genetic-based	Absent	90	27	39.161
1788	*Zonotrichia atricapilla*	Passerellidae	Genetic-based	Absent	90	28.5	49.023
1789	*Zonotrichia leucophrys*	Passerellidae	Genetic-based	Absent	90	30	44.899
1790	*Pipilo chlorurus*	Passerellidae	Genetic-based	Absent	90	30.5	33.232
1791	*Pipilo erythrophthalmus*	Passerellidae	Genetic-based	Absent	90	42	38.018
1792	*Pipilo maculatus*	Passerellidae	Genetic-based	Absent	90	40	34.226
1793	*Aimophila ruficeps*	Passerellidae	Genetic-based	Absent	90	19	28.298
1794	*Melozone fusca*	Passerellidae	Genetic-based	Absent	90	44.5	28.514
1795	*Melozone aberti*	Passerellidae	Genetic-based	Absent	90	47	34.424
1796	*Melozone crissalis*	Passerellidae	Genetic-based	Absent	90	53	33.021
1797	*Artemisiospiza belli*	Passerellidae	Genetic-based	Absent	90	15	33.790
1798	*Pooecetes gramineus*	Passerellidae	Genetic-based	Absent	90	24	38.797
1799	*Ammospiza leconteii*	Passerellidae	Genetic-based	Absent	90	13	44.420
1800	*Ammospiza maritima*	Passerellidae	Genetic-based	Partial	90	24	34.135
1801	*Ammospiza nelsoni*	Passerellidae	Genetic-based	Partial	90	16.5	44.187
1802	*Ammospiza caudacuta*	Passerellidae	Genetic-based	Absent	90	19	35.560
1803	*Passerculus sandwichensis*	Passerellidae	Genetic-based	Absent	90	23	43.519
1804	*Centronyx bairdii*	Passerellidae	Genetic-based	Absent	90	18	39.073
1805	*Centronyx henslowii*	Passerellidae	Genetic-based	Absent	90	13	35.961
1806	*Melospiza melodia*	Passerellidae	Genetic-based	Absent	90	32.5	40.778
1807	*Melospiza lincolnii*	Passerellidae	Genetic-based	Absent	90	19	40.032
1808	*Melospiza georgiana*	Passerellidae	Genetic-based	Absent	90	18.5	42.323

Supplementary Table 2. Examples of the mid-latitude index in species of different distribution patterns and life histories. The index is nagatively related to the duration of the time available for molting.

Description of the distribution pattern			Example	
**Main breeding area**	**Main wintering area**	**Migration distance**	**Species**	**Mid-distribution latitude**
N polar	S polar	long-distance	*Sterna paradisaea*	1.236
tropic	tropic	resident	*Hirundo aethiopica*	4.080
N temperate	tropic	long-distance	*Lanius senator*	24.732
N temperate	N temperate	resident	*Carduelis carduelis*	43.152
N temperate	N temperate	short-distacne	*Turdus merula*	48.252
S temperate	S temperate	resident	*Phalacrocorax atriceps*	52.274
N polar	N polar	short-distacne	*Pagophila eburnea*	67.422

Supplementary Table 3. List of the continuous-time Markov chain models. This list includes information about the log-likelihood and Akaike Information Criterion (AICc).

**Continuous-time Markov chain models**	**log-likelihood**	**AICc**	**ΔAICc**
Genetic-based and birth-death polytomy resolution (n = 1,808 species)			
All rates different model	-1000.66	2013.37	0.00
Equal transition rates model	-1027.44	2056.89	43.52
Symmetrical transition rates model	-1026.61	2059.24	45.87
Only genetic-based (n = 1,629 species)			
All rates different model	-919.09	1850.23	0.00
Equal transition rates model	-948.02	1898.04	47.81
Symmetrical transition rates model	-947.14	1900.29	50.06

Supplementary Table 4. List of the 27 bird orders that were included in the sample, the distribution of the molt strategy (complete, partial or absent) in each order and the result of the phylogenetic logistic regression (complete *versus* absent) for the three largest and most diverse orders.

	Nunber of species				Phylogenetic logistic regression			
					Body mass		Latitude	
	Complete molt	Partial molt	Absent molt	Total	***Z***	***P***	***Z***	***P***
Accipitriformes	0	7	49	56				
Anseriformes	0	0	72	72				
Bucerotiformes	1	0	0	1				
Caprimulgiformes	41	1	21	63	-1.06	0.289	1.30	0.193
Cathartiformes	0	0	3	3				
Charadriiformes	34	44	102	180	-0.80	0.425	-2.76	0.006
Ciconiiformes	0	0	1	1				
Columbiformes	15	5	0	20				
Coraciiformes	14	2	7	23				
Cuculiformes	5	6	2	13				
Falconiformes	1	0	17	18				
Galbuliformes	0	3	5	8				
Galliformes	2	23	0	25				
Gaviiformes	0	0	5	5				
Gruiformes	0	0	27	27				
Passeriformes	414	92	624	1130	-0.49	0.627	-2.94	0.003
Pelecaniformes	0	1	24	25				
Phaethontiformes	0	0	3	3				
Phoenicopteriformes	0	0	1	1				
Piciformes	0	31	10	41				
Podicipediformes	0	0	8	8				
Procellariiformes	2	0	30	32				
Psittaciformes	2	0	6	8				
Pterocliformes	0	0	1	1				
Strigiformes	0	1	22	23				
Suliformes	0	1	14	15				
Trogoniformes	1	0	5	6				

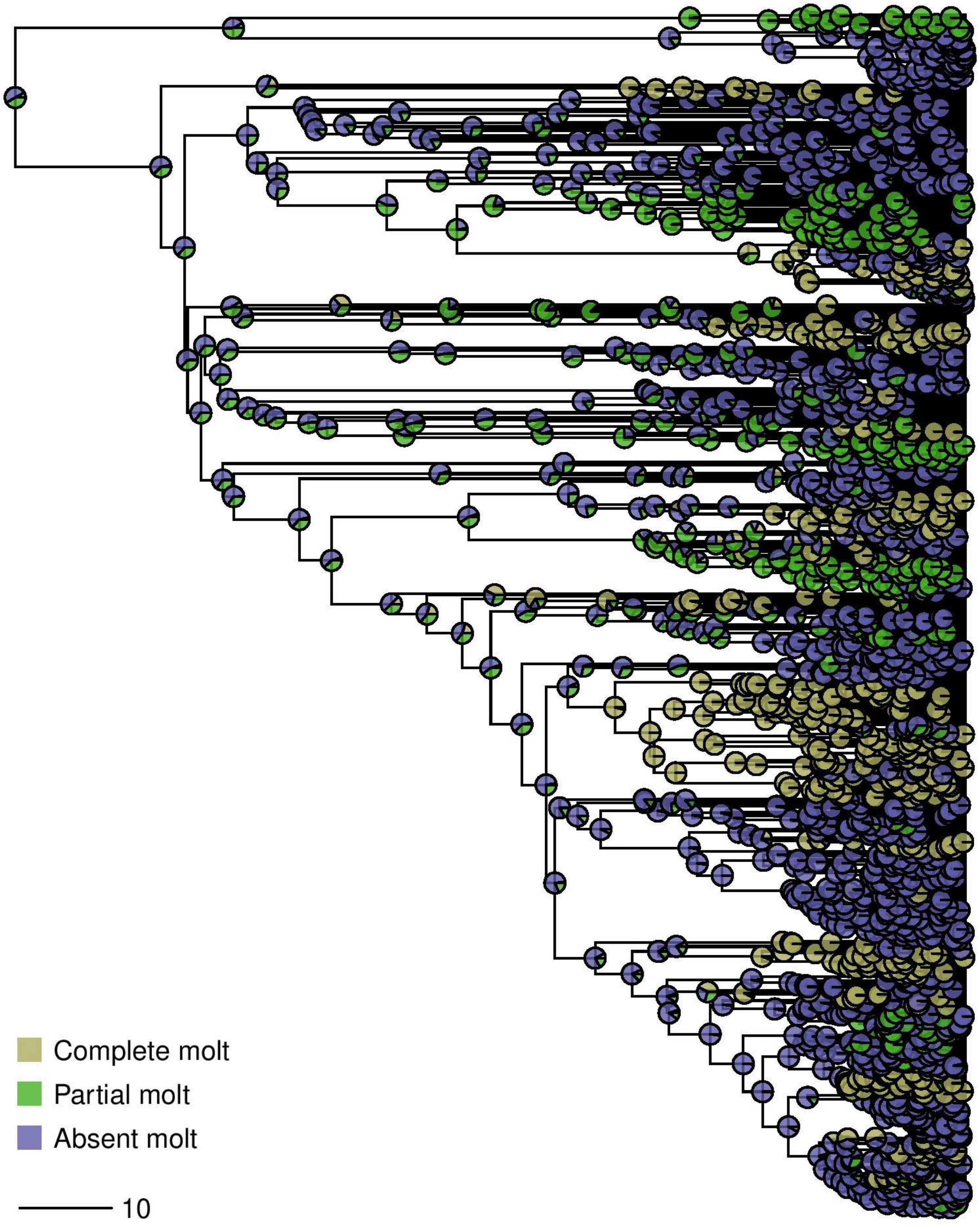

Supplementary Figure 1. The evolutionary history of molt strategies during the first year of life among modern birds (Neornithes). Ancestral trait reconstruction analysis (continuous-time Markov chain, all rates different model) including only the 1,629 species for which genetic data were available, after the exclusion of 179 species whose data were based on birth-death polytomy resolution. Pie charts at the nodes denote the posterior probabilities for each of three molt strategies. The scale (bottom left) represents 10 Myr.

Supplementary References

List of references for the molt strategy information in Supplementary Table 1.

1. Abe N, Higuchi H, Saito T, Matsuyama S, Sasagawa A. 1974. Moult of some birds captured at Subashiri banding area, Mt. Fuji. *Journal of the Yamashina Institute for Ornithology* **7**:404–426.
2. Alström P, Mild K. 2010. Pipits and Wagtails of Europe, Asia and North America. A&C Black.
3. Aourir M, Znari M, Radi M. 2018. Plumage development and moult in captive Black-bellied Sandgrouse *Pterocles orientalis*: age and sex variations. *Ostrich* **89**:329–337.
4. Baker K. 2010. Warblers of Europe, Asia and North Africa. A&C Black.
5. Baker K. 2016. Identification guide to European non-passerines. British Trust for Ornithology.
6. Balachandran S, Hussain SA. 1998. Moult, age structure, biometrics and subspecies of Lesser Sand Plover *Charadrius mongolus* wintering along the south-east coast of India. *Stilt* **33**:3–9.
7. Barshep Y, Manu SA. 2013. Breeding phenology and moult of the endemic Bannerman's Weaver *Ploceus bannermani*. *Ostrich* **84**:231–233.
8. Bendell JF. 1955. Age, molt and weight characteristics of blue grouse. *Condor* **57**:354–361.
9. Billerman SM, Keeney BK, Rodewald PG, Schulenberg TS. 2020. Birds of the World. Ithaca, New-York, USA: Cornell Laboratory of Ornithology.
10. Bonnevie BT. 2014. Variations in moult, morphology, movement and survival rates of the Sombre Greenbul Andropadus importunus within southern Africa. *Ostrich* **85**:161–170.
11. Braun CE, Tomlinson RE, Wann GT. 2015. Seasonal dynamics of mourning dove (*Zenaida Macroura*) body mass and primary molt. *Wilson Journal of Ornithology* **127**:630–638.
12. Britton PL, Sugg MSJ. 1973. Birds recorded on the Kimilili track, Mt. Elgon, Kenya. *Journal of East African Natural History* **143**:1–7.
13. Burger J, Howe M. 1975. Notes on winter feeding behavior and molt in Wilson's Phalaropes. *Auk* **92**:442–451.
14. Burton J, McNeil R. 1976. Age determination of six species of North American shorebirds. *Bird-banding* 201–209.
15. Craig A. 1983. Moult in southern African passerine birds: a review. *Ostrich* **54**:220–237.
16. Craig A. 1996. The annual cycle of wing-moult and breeding in the Wattled Starling *Creatophora cinerea*. *Ibis* **138**:448–454.
17. Cramp S, Simmons KEL. 1977. Handbook of the birds of Europe, the Middle East and North

Africa: The birds of the Western Palearctic. Oxford University Press.
18. Crawford RJM, Underhill LG. 2003. Aspects of breeding, molt, measurements and population trend of Hartlaub's Gull in Western Cape, South Africa. *Waterbirds* 139–149.
19. Cueva D. 2018. Molt strategy and aging of Rufous-collared Sparrow (*Zonotrichia capensis*) in Bogotá, Colombia. *Caldasia* **40**:18–26.
20. De La Cruz Solis C, de Lope Rebollo F, da Silva Rubio E. 1991. Sexual dimorphism in the post-juvenile moult in the Azure-winged Magpie *Cyanopica cyanea cooki*. *Ringing & Migration* **12**:86–91.
21. Debus SJS. 1989. Plumages and moult of the Little Eagle *Hieraaetus morphnoides*. *Australian Bird Watcher* **13**:103–113.
22. Delhey K, Guallar S, Rueda-Hernández R, Valcu M, Wang D, Kempenaers B. 2020. Partial or complete? The evolution of post-juvenile moult strategies in passerine birds. *Journal of Animal Ecology* **89**:2896–2908.
23. Diaz OA, Hernández F, Alza L, Chumpitaz K, Salvador J, Berrocal E, Tenorio Y, Poma T, Santos C, Iannacone J. 2018. Age Determination Based On Molt Patterns And Skull Ossification In The Blue-And-Yellow Tanager (*Pipraeidea bonariensis*). *Ornitologia Neotropical* **29**:51–61.
24. Dowsett RJ, Dowsett-Lemaire F. 1984. Breeding and moult cycles of somes montane forest birds in south-central Africa. *Revue d'Ecologie* **39**:89–111.
25. DuVal EH. 2005. Age-based plumage changes in the Lance-tailed Manakin: A two-year delay in plumage maturation. *Condor* **107**:915–920.
26. Edelstam C. 1984. Patterns of Moult in Large Birds of Prey. In Annales Zoologici Fennici (pp. 271–276). Finnish Academy of Sciences.
27. Filardi CE, Rohwer S. 2001. Life history implications of complete and incomplete primary molts in Pelagic Cormorants. *Condor* **103**:555–569.
28. Fogden MPL. 1972. The seasonality and population dynamics of equatorial forest birds in Sarawak. *Ibis* **114**:307–343.
29. Forsman D. 1981. Molt of the Spotted owl. *Auk* **98**:735–742.
30. Forsman D. 2016. Flight identification of raptors of Europe, North Africa and the Middle East. Bloomsbury Publishing.
31. Franklin DC, Legge S, Skroblin A, Heathcote J, Maute K, Schaefer DJ, Garnett ST. 2017. Wings of tropical finches: interspecific differences in shape are consistent with levels of mobility,

but moult and feather fault patterns are more complex. *Emu* **117**:370–381.
32. Freed LA, Cann RL. 2012. Changes in timing, duration, and symmetry of molt of Hawaiian forest birds. *PLoS One* **7**:e29834.
33. Fry CH. 2010. The Bee-Eaters. A&C Black.
34. Fullagar PJ, Dawkins MJ, Minton CDT. 2013. Biometrics and wing molt in White-winged Black Tern (*Chlidonias leucopterus*) in north-west Australia. *Chinese Birds* **4**:306–313.
35. Gardner JL, Marsack PR, Blackmore CJ. 2008. Timing and sequence of moult, and the trade-off with breeding, in the Speckled Warbler (*Chthonicola sagittata*). *Emu* **108**:90–96.
36. Gaston AJ. 1981. Seasonal breeding, moulting and weight changes among birds of dry deciduous forest in North India. *Journal of Zoology* **194**:219–243.
37. Ginn HB, Melville DS. 1983. Moult in birds (BTO guide). Tring, UK: British Trust for Ornithology.
38. Guallar S, Santana E, Martinez SC, Mungria HV, Galles A, de Ciencias Naturales M. 2009. Paseriformes del Occidente de México: morfometría, datación y sexado. Ajuntament de Barcelona, Institut de Cultura.
39. Guallar S, Ruiz-Sánchez A, Rueda-Hernández R, Pyle P. 2016. Molt strategies of ten neotropical forest passerine species. *Wilson Journal of Ornithology* **128**:543–555.
40. Guallar S, Ruiz-Sánchez A, Rueda-Hernández R, Pyle P. 2018. Preformative wing molt in 23 Neotropical resident passerine species. *Ornitologia Neotropical* **29**:3–10.
41. Guallar S, Rueda-Hernández R, Pyle P. 2021. Evolution of the preformative molt in Cardinalidae correlates with transitions from forest to open habitats. *Auk* **138**:ukaa070.
42. Hanmer DB. 1978. Measurements and moult of five species of bulbul from Mozambique and Malawi. *Ostrich* **49**:116–131.
43. Hanmer DB. 1980. Mensural and moult data on six species of bee-eater in Mozambique and Malawi. *Ostrich* **51**: 25–38.
44. Haran R. 2011. A Guide for Bird-Ringing in Israel. Jerusalem.
45. Hernández A. 2012. Molt patterns and sex and age criteria for selected landbirds of southwest Colombia. *Ornitologia Neotropical* **23**:215–223.
46. Herremans M. 1995. Descendant eccentric partial post-juvenile primary moult in the Black-cheeked Waxbill *Estrilda erythronotos*. *Safring News* **24**:13–14.
47. Herremans M. 1995. The use of plumage features resulting from a partial post-juvenile moult

in age determination of southern African Passerines. *Safring News* **24**:19–22.

48. Herremans M. 1999. Biannual complete moult in the Black-chested Prinia *Prinia flavicans*. *Ibis* **141**:115–124.

49. Herremans M. 2000. Cases of serial descendant primary moult (Staffelmauser) in the Black-shouldered Kite *Elanus caeruleus*. *Ringing & Migration* **20**:15–18.

50. Hörnfeldt B, Carlsson B-G, Nordström Å. 1988. Molt of primaries and age determination in Tengmalm's owl (*Aegolius funereus*). *Auk* 783–789.

51. Houston DC. 1975. The moult of the White-backed and Rüppell's Griffon Vultures *Gyps africanus* and *G. Rueppellii*. *Ibis* **117**:474–488.

52. Howell SNG. 2001. Molt of the Ivory gull. *Waterbirds* 438–442.

53. Insley H, Young L, Dudley B. 1980. Primary moult in the Collared Dove. *Bird Study* **27**:101–107.

54. Jackson HD. 2008. Moult and ectoparasites of nightjars collected during two January expeditions across Zimbabwe. *Ostrich* **79**:91–100.

55. Jenni L, Winkler R. 2020. Moult and ageing of European passerines. Bloomsbury Publishing.

56. Johnsgard PA. 1983. Cranes of the world: Eurasian Crane (*Grus grus*).

57. Johnson EI, Wolfe JD. 2017. Molt in Neotropical birds: Life history and aging criteria. CRC Press.

58. Johnson OW, Johnson PM. 1983. Plumage-molt-age relationships in 'over-summering' and migratory Lesser Golden-Plovers. *Condor* **85**:406–419.

59. Jones PJ. 1980. The timing of wing moult in the greyhooded kingfisher in Nigeria. *Ostrich* **51**:99–106.

60. Kamtaeja S, Suwannapoom C, Sitasuwan N, Chomdej S. 2015. Moult in the Stripe-throated Bulbul, *Pycnonotus finlaysoni*: Sexual Differences and Timing. *Chiang Mai Journal of Science* **42**:339–348.

61. Kang S-G, Hur W-H. 2017. New moult pattern in diurnal raptors: primary moult pattern of the Japanese Sparrowhawk *Accipiter gularis*. *Ringing & Migration* **32**:28–36.

62. Kawaji N, Hirokawa J. 1998. Complete post-juvenile moult of the Asian Stubtail Urosphena squameiceps. *Bulletin of the Japanese Bird Banding Association* **13**:1–7.

63. Kennedy JJ, Heavyside J, Jankowski JE, Scholer MN. 2018. Molt patterns and sexing and aging criteria for ten species of high elevation landbirds from southeastern Peru. *Ornitologia*

Neotropical **29**:63–73.

64. Kennerley P, Pearson D. 2010. Reed and bush warblers. A&C Black.

65. Kiat Y, Izhaki I. 2015. Post-juvenile moult in Graceful Prinia *Prinia gracilis*. *Ringing & Migration* **30**:7–11.

66. Kiat Y, Perlman Y. 2016. Moult and ageing of Isabelline Shrikes. *British Birds* **109**:157–168.

67. Langston NE, Rohwer S. 1995. Unusual patterns of incomplete primary molt in Laysan and Black-footed Albatrosses. *Condor* **97**:1–19.

68. Laycock HT. 1982. Moulting and plumage changes in the Thick-billed Weaver. *Ostrich* **53**:91–101.

69. Lewis J. 2010. otes on the moult and biology of the Red-headed Honeyeater (*Myzomela erythrocephala*) in the west Kimberley, Western Australia. *Amytornis* **2**:15–24.

70. Louette M. 2003. Size, plumage, moult and supposed hybrids of African Goshawks (*Accipiter tachiro*/*toussenelii* group) in DR Congo. *Ostrich* **74**:18–29.

71. Louette M. 2006. Moult, pied plumage and relationships within the genus of the Black Sparrowhawk *Accipiter melanoleucus*. *Ostrich* **77**:73–83.

72. Marchant, S., & Higgins, P. J. 1990. Handbook of Australian, New Zealand and Antarctic Birds. Oxford University Press.

73. Marks JS. 1993. Molt of Bristle-thighed Curlews in the northwestern Hawaiian Islands. *Auk* **110**:573–587.

74. McInnes AM, Allan DG, Underhill LG. 2014. Moult of the Grey-headed Gull *Larus cirrocephalus* in South Africa. *Durban Natural Science Museum Novitates* 40.

75. Mullié WC, Buij R, Cavaillès S. 2014. Chick development, growth and post-juvenile moult of the African Swallow-tailed Kite *Chelictinia riocourii* in Senegal and Cameroon. *Malimbus* **36**:1–12.

76. Nakamura H. 1979. Summer Concentration and Moult in the Oriental Greenfinch *Carduelis sinica*. *Japanese Journal of Ornithology* **28**:1–27.

77. Niles DM. 1972. Molt cycles of purple martins (*Progne subis*). *Condor* **74**:61–71.

78. Norevik G, Hellström M, Liu D, Petersson B. 2020. Ageing & Sexing of Migratory East Asian Passerines. Avium förlag AB. Sweden.

79. Olsen KM. 2010. Gulls of Europe, Asia and North America. Bloomsbury Publishing.

80. Owen Jr RB, Krohn WB. 1973. Molt patterns and weight changes of the American Woodcock.

Wilson Bulletin 31–41.
81. Page G. 1974. Molt of wintering least sandpipers. *Bird-Banding* 93–105.
82. Park JG, Park CU, Jin KS, Kim YM, Kim HY, Jeong SY, Nam DH. 2020. Moult and plumage patterns of the critically endangered Yellow-breasted Bunting (*Emberiza aureola*) at a stopover site in Korea. *Journal of Ornithology* **161**:257–266.
83. Pezzo F, Gosler AG. 2005. Evidence of prenuptial moult in the Little Bittern *Ixobrychus minutus*. *Ringing & Migration* **22**:129–132.
84. Pierce AJ. 2009. Observations on breeding and moulting of the Grey-eyed Bulbul, *Iole propinqua*, in Thailand. *Raffles Bulletin of Zoology* **57**:207–211.
85. Pople RG. 2003. The ecology and conservation of the White-winged Nightjar *Caprimulgus candicans*. Ph.D. thesis. Cambridge, UK.
86. Prince PA, Rodwell S, Jones M, Rothery P. 1993. Moult in Black-browed and Grey-headed Albatrosses *Diomedea melanophris* and *D. chrysostoma*. *Ibis* **135**:121–131.
87. Prout-Jones DV, Milstein PLS. 1986. Sequential moult with age-class establishment in the African Fish Eagle *Haliaeetus vocifer*. *African Journal of Wildlife Research* **16**:17–26.
88. Pyle P. 1995. Incomplete flight feather molt and age in certain North American non-passerines. *North American Bird Bander* **20**.
89. Pyle P, Howell SNG. 1995. Flight-Feather Molt Patterns and Age in North American Woodpeckers. *Journal of Field Ornithology* **66**:564–581.
90. Pyle P. 1997. Identification guide to North American birds: a compendium of information on identifying, ageing, and sexing 'near-passerines' and passerines in the hand. Slate Creek Press.
91. Pyle P. 1997. Molt limits in North American passerines. *North American Bird Bander* **22**:49–89.
92. Pyle P, Howell SNG, Yanega GM. 1997. Molt, retained flight feathers, and age in North American hummingbirds. *Era Allan R Phillips A Festschrift RW Dickerman, Albuquerque* 155–166.
93. Pyle P, McAndrews A, Veléz P, Wilkerson RL, Siegel RB, DeSante DF. 2004. Molt patterns and age and sex determination of selected southeastern Cuban landbirds. *Journal of Field Ornithology* **75**:136–145.
94. Pyle P. 2005. Molts and plumages of ducks (Anatinae). *Waterbirds* **28**:208–219.
95. Pyle P. 2005. First-cycle molts in North American Falconiformes. *Journal of Raptor Research*

39:378.

96. Pyle P, Howell SNG, Sesante DF, Ruck S. 2008. Identification guide to North American birds: Anatidae to Alcidae. Slate Creek Press.

97. Pyle P. 2009. Age determination and molt strategies in North American alcids. *Marine Ornithology* **37**:219–226.

98. Pyle P, Engilis Jr A, Kelt DA. 2015. Manual for ageing and sexing the landbirds of Bosque Fray Jorge National Park and north-central Chile, with notes on range and breeding seasonality. Museum of Natural Science, Louisiana State University.

99. Pyle P, Tranquillo K, Kayano K, Arcilla N. 2016. Molt patterns, age criteria, and molt-breeding dynamics in American Samoan landbirds. *Wilson Journal of Ornithology* **128**:56–69.

100. Pyle P, Kayano K, Tranquillo K, Murphy K, Wilcox B, Arcilla N. 2017. Manual for ageing and sexing landbirds of American Samoa, with notes on molt and breeding seasonality. Institute for Bird Populations, Point Reyes Station.

101. Radley P, Crary AL, Bradley J, Carter C, Pyle P. 2011. Molt patterns, biometrics, and age and gender classification of landbirds on Saipan, Northern Mariana Islands. *Wilson Journal of Ornithology* **123**:588–594.

102. Rasmussen PC. 1988. Stepwise molt of remiges in Blue-eyed and King Shags. *Condor* **90**:220–227.

103. Rohwer S, Rohwer VG. 2018. Breeding and multiple waves of primary molt in common ground doves of coastal Sinaloa. *PeerJ* **6**:e4243.

104. Round PD, Gale GA, Nimnuan S. 2012. Moult of primaries in Long-toed Stints (*Calidris subminuta*) at a non-breeding area in Thailand. *Ringing & Migration* **27**:32–37.

105. Round PD, Nimnuan S, Phothieng D, Chunkao K. 2014. Moult in the Asian Pied Starling *Sturnus contra floweri* population of Thailand. *Forktail* **30**:28–33.

106. Ruiz-Sanchez A, Rueda-Hernández R, Guallar S, Pyle P. 2012. Age determination of the Spot-breasted Wren and the White-breasted Wood-Wren using molt limits. *North American Bird Bander* **37**:93–100.

107. Ryder TB, Durães R. 2005. It’s not easy being green: using molt and morphological criteria to age and sex green-plumage manakins (Aves: Pipridae). *Ornitologia Neotropical* **16**:481–491.

108. Ryder TB, Wolfe J. 2009. The current state of knowledge on molt and plumage sequences in selected tropical families: a review. *Ornitologia Neotropical* **20**:1–18.

109. Schondube JE, Santana C E, Ruán-Tejeda I. 2003. Biannual Cycles of the Cinnamon-bellied Flowerpiercer. *Biotropica* **35**:250–261.

110. Serra L, Clark NA, Clark JA. 2006. Primary moult, body mass and migration of Grey Plovers *Pluvialis squatarola* in Britain. *Ibis* **148**:292–301.

111. Shirihai H, Gargallo G, Helbig AJ. 2001. Sylvia Warblers: Identification, Taxonomy and Phylogeny of the Genus Sylvia. A&C Black.

112. Shirihai H, Svensson L. 2018. Handbook of Western Palearctic Birds. Bloomsbury Publishing.

113. Shugart GW, Rohwer S. 1996. Serial descendant primary molt or Staffelmauser in Black-crowned Night-Herons. *Condor* **98**:222–233.

114. Siegfried WR. 1971. Moult of the primary remiges in three species of *Streptopelia* doves. *Ostrich* **42**:161–165.

115. Slack RC. 1992. Primary molt patterns of northern saw-whet owls (*Aegolius acadicus*) captured during spring migration. *North American Bird Bander* **17**:97–101.

116. Smith C, Walker LK, Ewen JG. 2015. Age and sex criteria for the hihi (*Notiomystis cincta*) with additional details on moult patterns. *Notornis* **62**:135–142.

117. Smith GT. 1985. Natal downs and plumage changes in the Noisy Scrub-bird, *Atrichornis clamosus* (Passeriformes: Atrichornithidae). *Records of the Australian Museum* **37**:157.

118. Snyder NFR, Johnson E V, Clendenen DA. 1987. Primary molt of California Condors. *Condor* **89**:468–485.

119. Solheim R. 2012. Wing feather moult and age determination of Snowy Owls *Bubo scandiacus*. *Ornis Norvegica* **35**:48–67

120. Stiles FG. 1980. The annual cycle in a tropical wet forest hummingbird community. *Ibis* **122**:322–343.

121. Stutchbury BJ, Rohwer S. 1990. Molt patterns in the Tree Swallow (*Tachycineta bicolor*). *Canadian Journal of Zoology* **68**:1468–1472.

122. Svensson L. 1992. Identification Guide to European Passerines. Stockholm: Ugga.

123. Trefry HE, Holroyd GL. 2012. Molt in Burrowing Owls (*Athene cunicularia*). *North American Bird Bander* **37**:4–10.

124. Turner A. Rose C. 2010. A Handbook to the Swallows and Martins of the World. A&C Black.

125. Tyler SJ, Tyler L. 2001. Biometrics and moult data for nomadic Lark-like Buntings, *Emberiza*

impetuani, in southeastern Botswana. *Ostrich* **72**:118–120.

126. Tyler SJ. 2002. Ringing in Acacia savanna at Ruretse, southeast Botswana, 1996–2000. *Afring News* **31**:19–23.

127. Ursino C, Facchinetti C, Reboreda JC. 2012. Preformative molt in brood parasitic screaming (*Molothrus rufoaxillaris*) and shiny (*M. bonariensis*) cowbirds. *Ornitologia Neotropical* **23**:163–171.

128. Vellenga RE. 1980. Moults of the Satin Bowerbird *Ptilonorhynchus violaceus*. *Emu* **80**:49–54.

129. Ward P. 1969. The annual cycle of the Yellow-vented bulbul *Pycnonotus goiavier* in a humid equatorial environment. *Journal of Zoology* **157**:25–45.

130. Weller MW. 1965. Bursa regression, gonad cycle and Molt of the Great-horned Owl. *Bird-Banding* **36**:102–112.

131. Wilkinson R. 1983. Biannual breeding and moult-breeding overlap of the Chestnut-bellied Starling *Spreo pulcher*. *Ibis* **125**:353–361.

132. Williams Jr LE, Austin DH. 1970. Complete post-juvenal (pre-basic) primary molt in Florida Turkeys. *Journal of Wildlife Management* 231–233.

133. Willoughby EJ. 1971. Biology of larks (Aves: Alaudidae) in the central Namib Desert. *African Zoology* **6**:133–176.

134. Wolfe JD, Chandler RB, King DI. 2009. Molt patterns, age, and sex criteria for selected highland Costa Rican resident landbirds. *Ornitologia Neotropical* **20**:451–459.

135. Wolfe JD, Pyle P, Ralph CJ. 2009. Breeding seasons, molt patterns, and gender and age criteria for selected northeastern Costa Rican resident landbirds. *Wilson Journal of Ornithology* **121**:556–567.

136. Wyndham E, Brereton JLG, Beeton RJS. 1983. Moult and plumages of Eastern rosellas *Platycercus eximius*. *Emu* **83**:242–246.

137. Yamashina Y. 1980. A natural history of Japanese birds. Shuppan Kagaku.

138. Yuri T, Rohwer S. 1997. Molt and migration in the Northern Rough-winged Swallow. *Auk* **114**:249–262.

139. Zann R. 1985. Slow continuous wing-moult of Zebra Finches *Poephila guttata* from southeast Australia. *Ibis* **127**:184–196.

140. Zuberogoitia I, de La Puente J, Elorriaga J, Alonso R, Palomares LE, Martínez JE. 2013. The

flight feather molt of Griffon Vultures (*Gyps fulvus*) and associated biological consequences. *Journal of Raptor Research* **47**:292–303.

141. Zuberogoitia I, Gil JA, Martínez JE, Erni B, Aniz B, López-López P. 2016. The flight feather moult pattern of the bearded vulture (*Gypaetus barbatus*). *Journal of Ornithology* **157**:209–217.

142. Authors Data.
